# Supplementary material for: Polyphenols with Anti-Inflammatory Properties: Synthesis and Biological Activity of Novel Curcumin Derivatives
Source: Int J Mol Sci. 2023 Feb 12;24(4):3691. doi: 10.3390/ijms24043691 (PMC9966181; doi:10.3390/ijms24043691)
Supplement: Supplementary file 1 [file ijms-24-03691-s001.zip › ijms-2122582-supplementary.pdf]

Polyphenols with Anti-Inflammatory Properties: Synthesis and Biological Activity of Novel Curcumin Derivatives.

Yisett González [1,2], Randy Mojica-Flores [3], Dilan Moreno-Labrador [1], Luis Cubilla-Rios [2,4], K.S. Jagannatha Rao [5,6], Patricia L. Fernández [1,2], Oleg V. Larionov [7,*] and Johant Lakey-Beitia [2,3,*]

[1] Center for Molecular and Cellular Biology of Diseases, Instituto de Investigaciones Científicas y Servicios de Alta Tecnología (INDICASAT AIP), Clayton, City of Knowledge, Panama City 0843-01103, Panama
[2] Sistema Nacional de Investigación (SNI), SENACYT, Panama City 0816-02852, Panama
[3] Center for Biodiversity and Drug Discovery, Instituto de Investigaciones Científicas y Servicios de Alta Tecnología (INDICASAT AIP), Clayton, City of Knowledge, Panama City 0843-01103, Panama
[4] Laboratory of Tropical Bioorganic Chemistry, Faculty of Natural, Exact Sciences and Technology, University of Panama, Panama City 0824-03366, Panama
[5] Center for Neuroscience, Instituto de Investigaciones Científicas y Servicios de Alta Tecnología (INDICASAT AIP), Clayton, City of Knowledge, Panama City 0843-01103, Panama
[6] Department of Biotechnology, Koneru Lakshmaiah Education Foundation (KLEF) Deemed to be University, Vaddeswaram 522302, India
[7] Department of Chemistry, The University of Texas at San Antonio, San Antonio, TX 78249, USA
* Correspondence: oleg.larionov@utsa.edu (O.V.L.); jlakey@indicasat.org.pa (J.L.-B.); Tel.: +1-210-458-6050 (O.V.L.); +507-517-0700 (J.L.-B.)

General Procedures

Materials: Chemical reagents were used as commercially available. Chemical reagents were used as commercially available (Tedia, Applichem, Sigma Aldrich). Curcumin was obtained comercially in Alfa Aesar with 95% (total curcuminoid content) from Turmeric rhizome.

Experimental equipment: All reactions were conducted in borosilicate glass tube (20 mL or 16 mL) fitted with screw-cap and magnetic stirring under the atmosphere of argon.

HPLC Analysis: The reaction mixture was evaluated in an Agilent 1260 Infinity II HPLC system equipped with a quaternary pump, an Agilent diode array detector 1260 Series and normal phase silica gel column (Phenomenex® Luna Silica (2), 250 × 10 mm, 5 µm) with gradient system *n*-hexane to ethyl acetate in 20 min at 2 mL/min (Agilent Technologies, Santa Clara, CA, USA).

Purification: The reaction mixture was purified using a Buchi C-815 Flash HPLC system equipped with a binary pump, a UV scan and an Evaporative Light Scattering (ELSD) detector, a closed fraction collector bay and normal phase silica gel cartridge (Buchi® FlashPure EcoFlex Silica 12 g, 40-63 µm) in an isocratic system *n*-hexane/ethyl acetate 7:3 BÜCHI Labortechnik AG, Meierseggstrasse 40 postfach, *Switzerland*).

Characterization: ^1H, ^{13}C NMR spectra were recorded at 500 (^1H), 125 MHz (^{13}C) on a Jeol JNM-ECZ500R/S1 500 MHz spectrometer in $CDCl_3$ and DMSO-d_6 (JEOL Ltd. 3-1-2 Musashino, Akishima, Tokyo 196-8558, Japan). Chemical shifts (δ) are reported in parts per million (ppm) from the residual solvent peak and coupling constant (*J*) in Hz. Proton multiplicity is reported in: singlet (s), doublet (d), triplet (t), quartet (quart.), quintet (quint.), septet (sept), multiplet (m), broad (br). Infrared measurements were carried out on a Bruker Platinum ATR Alpha instrument (Bruker, Billerica, MA, USA). The MS analyses were carried out on a Waters Xevo TQD spectrometer with Electrospray Ionization (ESI) as an ion source (Waters Corporation, Milford, MA, USA). Melting point determinations were measured by triplicate on an Automatic Melting Point apparatus Stuart SMP50 (Cole-Palmer, Staffordshire, UK).

General procedure 1 (GP1) for the synthesis of Alkyl succinate monoesters (S1-S9):

An oven dried vial (20 mL) fitted with screw-cap with magnetic stirrer was flushed with argon and charged with adamantan-2-ol (500.0 mg, 3.28 mmol), and *N,N*-diisopropylethylamine (858 μL, 4.92 mmol, 1.5 equiv.) were stirred in pyridine (4 mL) at room temperature (RT). After 2 h, succinic anhydride (492.9 mg, 4.92 mmol, 1.5 equiv.) and 4-dimethylaminopyridine (601.8 mg, 4.92 mmol, 1.5 equiv.) were added, and the reaction stirred at RT. After 48 h, the reaction mixture was diluted with brine/1M HCl (3:1, 10 mL). The aqueous layer was extracted with dichloromethane (3 × 20 mL), and the combined organic phases dried over anhydrous sodium sulfate (Na_2SO_4) and concentrated under reduced pressure to obtain the desired product (Figure supplementary 1).

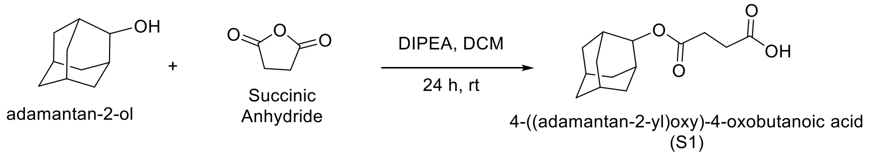

Figure Supplementary 1. Methodology of synthesis of Alkyl succinate monoester (S1-S9).

4-((adamantan-2-yl)oxy)-4-oxobutanoic acid (S1)

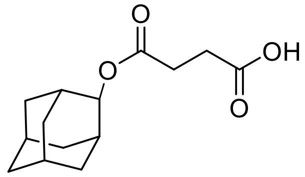

According to GP1, adamantan-2-ol (500.0 mg, 3.28 mmol), and *N,N*-diisopropylethylamine (858 µL, 4.92 mmol, 1.5 equiv.) were stirred in pyridine (4 mL) at RT. After 2 h, succinic anhydride (492.9 mg, 4.92 mmol, 1.5 equiv.) and 4-dimethylaminopyridine (601.8 mg, 4.92 mmol, 1.5 equiv.) were added, and the reaction mixture stirred for 48 h at RT to obtain the monoester S1 (713.3 mg, 86%). ^{1}H NMR (500 MHz, DMSO-D_6): δ 4.76 (t, *J* = 3.2 Hz, 1H), 2.49 – 2.44 (m, 5H), 1.95 – 1.84 (m, 4H), 1.79 – 1.63 (m, 8H), 1.47 (d, *J* = 11.5 Hz, 2H) ppm. ^{13}C NMR (125 MHz, DMSO-*D6*): 26.4, 26.6, 28.9, 29.3, 31.2, 35.7, 36.8, 76.2, 171.3, 173.5. IR: 2901.31, 1702.61, 1699.69, 1322.74, 1162.63. mp: 87.8 ± 1.5 °C. MS (m/z) calcd for $C_{14}H_{20}NaO_4$: 275.30; found: 274.99 [M+Na$^+$].

4-((9,10-dioxo-9,10-dihydroanthracen-2-yl)methoxy)-4-oxobutanoic acid (S2)

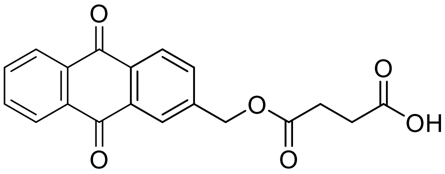

According to GP1, 2-(hydroxymethyl)anthracene-9,10-dione (200.9 mg, 0.84 mmol), and *N,N*-diisopropylethylamine (500 µL, 2.87 mmol, 3.4 equiv.) were stirred in CH_2Cl_2 (5 mL) at RT. After 2 h, succinic anhydride (251.9 mg, 2.52 mmol, 3.0 equiv.) and 4-dimethylaminopyridine (307.6 mg, 2.52 mmol, 3.0 equiv.) were added, and the reaction mixture stirred for 48 h at RT to yield monoester S2 (255.2 mg, 89%). ^{1}H NMR (500 MHz, DMSO-D_6): δ 8.14 – 8.05 (m, 4H), 7.87 – 7.82 (m, 2H), 7.79 (d, *J* = 9.7 Hz, 1H), 5.23 (s, 2H), 2.61 – 2.56 (m, 2H), 2.49 – 2.44 (m, 2H) ppm. ^{13}C NMR (125 MHz): 28.72, 28.74, 64.5, 125.4,

126.7, 126.8, 127.1, 132.5, 133.01, 133.04, 133.0, 133.1, 134.6, 134.7, 143.2, 172.1, 173.5, 182.2, 182.3 ppm. IR: 2932.30, 1684.14, 1671.36, 1652.92, 1591.01, 1175.53, 705.69. mp: 152.1 ± 1.1 ^{o}C.

4-(benzhydryloxy)-4-oxobutanoic acid (S3)

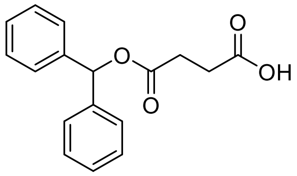

According to GP1, diphenylmethanol (1380.6 mg, 7.49 mmol), and *N,N*-diisopropylethylamine (870 μL, 5.00 mmol, 0.67 equiv.) were stirred in CH_2Cl_2 (4 mL) at RT. After 2 h, succinic anhydride (500 mg, 5.00 mmol, 0.67 equiv.) and 4-dimethylaminopyridine (610.4 mg, 5.00 mmol, 0.67 equiv.) were added, and the reaction mixture stirred for 48 h at RT to yield monoester S3 (1865.3 mg, 88%). ^{1}H NMR (500 MHz, DMSO-*D*$_6$): δ 7.35 – 7.28 (m, 10H), 6.75 (s, 1H), 2.64 – 2.60 (m, 2H), 2.50 – 2.47 (m, 2H). ^{13}C NMR (125 MHz, DMSO-*D*$_6$): δ 173.39, 171.19, 140.57, 128.48, 127.70, 126.48, 76.45, 29.02, 28.67 ppm. IR: 3059.77, 3028.62, 1733.35, 1240.98, 1154.35, 694.86. MS (m/z) calcd for $C_{17}H_{16}NaO_4$: 307.3; found: 306.9 [M+Na$^+$].

4-(cyclohexyl(phenyl)methoxy)-4-oxobutanoic acid (S4)

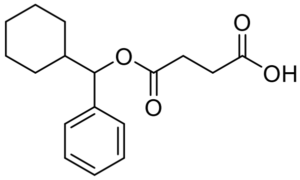

According to GP1, cyclohexyl(phenyl)methanol (50.0 mg, 0.26 mmol), and *N,N*-diisopropylethylamine (100 μL, 0.57 mmol, 2.1 equiv.) were stirred in CH_2Cl_2 (2 mL) at RT. After 2 h, succinic anhydride (80.0 mg, 0.80 mmol, 3.0 equiv.) and 4-dimethylaminopyridine (100 mg, 0.82 mmol, 3.0 equiv.) were added, and the reaction mixture stirred for 48 h at RT to yield monoester S4 (74.8 mg, 98%). ^{1}H NMR (500 MHz, DMSO-*D*$_6$): δ 7.35 – 7.24 (m, 5H), 5.45 (d, *J* = 7.1 Hz, 1H), 2.58 – 2.52 (m, 2H), 2.49 – 2.45 (m, 2H), 1.75 – 1.55 (m, 5H), 1.31 – 0.93 (m, 6H) ppm.^{13}C NMR (125 MHz, DMSO-*D*$_6$): 25.3, 25.4, 25.8, 28.0, 28.6, 28.7, 28.9, 29.0, 42.5, 79.3, 126.6, 127.5, 128.1, 139.5, 171.3, 173.3, 173.6 ppm. IR: 2916.61, 2846.67, 1721.37, 1225.98, 1164.43, 695.52 cm^{-1}. mp: 156.1 ± 3.5 ^{o}C. MS (m/z) calcd for $C_{17}H_{22}NaO_4$: 313.3; found: 313.0 [M+Na$^+$].

4-((9H-fluoren-9-yl)oxy)-4-oxobutanoic acid (S5)

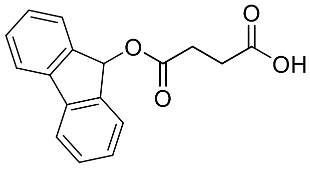

According to GP1, 9H-fluoren-9-ol (500 mg, 2.74 mmol), and *N,N*-diisopropylethylamine (717 µL, 4.12 mmol, 1.5 equiv.) were stirred in pyridine (4 mL) at RT. After 2 h, succinic anhydride (411.9 mg, 4.12 mmol, 1.5 equiv.) and 4-dimethylaminopyridine (502.8 mg, 4.12 mmol, 1.5 equiv.) were added, and the reaction mixture stirred for 48 h at RT to yield monoester S5 (600 mg, 77%). ^{1}H NMR (500 MHz, DMSO-D_6): δ 7.84 (d, *J* = 7.5 Hz, 2H), 7.52 (dd, *J* = 7.5, 1.0 Hz, 2H), 7.50 – 7.41 (m, 2H), 7.33 (td, *J* = 7.4, 1.1 Hz, 2H), 6.75 (s, 1H), 2.66 – 2.62 (m, 2H), 2.58 – 2.55 (m, 2H) ppm. ^{13}C NMR (125 MHz, DMSO-D_6): δ 173.6, 173.4, 141.8, 140.4, 129.6, 127.9, 125.7, 120.4, 74.5, 28.9. mp: 130.5 ± 0.1 °C. MS (m/z) calcd for $C_{17}H_{14}NaO_4$: 305.2; found: 304.9 [M+Na$^+$].

4-((2,3-dihydro-1H-inden-2-yl)oxy)-4-oxobutanoic acid (S6)

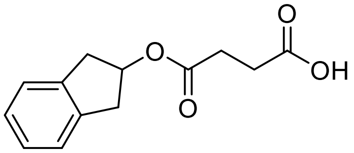

According to GP1, 2,3-dihydro-1H-inden-2-ol (500 mg, 3.73 mmol), and *N,N*-diisopropylethylamine (973 µL, 5.59 mmol, 1.5 equiv.) were stirred in CH_2Cl_2 (4 mL) at RT. After 2h, succinic anhydride (559.3 mg, 5.59 mmol, 1.5 equiv.) and 4-dimethylaminopyridine (682.8 mg, 5.59 mmol, 1.5 equiv.) were added, and the reaction mixture stirred for 48 h at RT to yield monoester S6 (817.8 mg, 94%). ^{1}H NMR (500 MHz, DMSO): δ 12.19 (s, 1H), 7.27 – 7.14 (m, 4H), 5.46 – 5.40 (m, 1H), 3.27 (dd, *J* = 17.1, 6.4 Hz, 2H), 2.89 (d, *J* = 17.0 Hz, 2H), 2.44 (s, 4H) ppm. ^{13}C NMR (125 MHz, DMSO): δ 173.4, 172.1, 140.4, 126.6, 124.5, 75.0, 28.8, 28.6 ppm. IR: 2943.05, 2910.11, 1716.85, 1652.88, 1398.03, 1165.52, 941.71, 748.10. mp: 112.8 ± 0.9 °C. MS (m/z) calcd for $C_{13}H_{14}NaO_4$: 257.2; found: 256.9 [M+Na$^+$].

4-(((1R,2S,5R)-2-isopropyl-5-methylcyclohexyl)oxy)-4-oxobutanoic acid (S7)

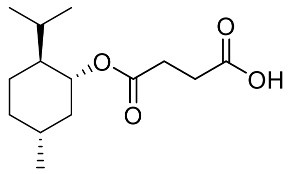

According to GP1, (1R,2S,5R)-(-)Menthol (500.0 mg, 3.20 mmol), and *N,N*-diisopropylethylamine (836 μL, 4.80 mmol, 1.5 equiv.) were stirred in CH_2Cl_2 (4 mL) at RT. After 2 h, succinic anhydride (480.2 mg, 4.80 mmol, 1.5 equiv.) and 4-dimethylaminopyridine (586.3 mg, 4.80 mmol, 1.5 equiv.) were added, and the reaction mixture stirred for 48 h at RT to yield monoester S7 (774.1 mg, 94 %). ^{1}H NMR (500 MHz, DMSO): δ 4.63 – 4.54 (m, 1H), 2.47 (s, 4H), 1.90 – 1.79 (m, 2H), 1.67 – 1.58 (m, 2H), 1.50 – 1.28 (m, 2H), 1.08 – 0.90 (m, 2H), 0.90 – 0.83 (m, 7H), 0.71 (d, *J* = 6.9 Hz, 3H). ^{13}C NMR (125 MHz, DMSO): δ 73.2, 46.4, 40.5, 33.7, 30.8, 29.0, 28.7, 25.7, 23.0, 21.9, 20.5, 16.3. IR: 2949.05, 2868.19, 1706.67, 1699.11, 1652.74, 1387.20, 1169.71, 952.45. mp: not determined. MS (m/z) calcd for $C_{14}H_{24}NaO_4$: 279.33; found: 279.03 [M+Na$^+$].

4-methoxy-4-oxobutanoic acid (S8)

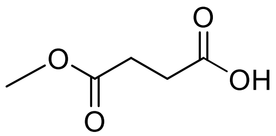

According to GP1, methanol (616.8 μL, 15.6 mmol), and *N,N*-diisopropylethylamine (2249 μL, 12.9 mmol, 0.8 equiv.) were stirred in CH_2Cl_2 (4 mL) at RT. After 2 h, succinic anhydride (1292.2 mg, 12.9 mmol, 0.8 equiv.) and 4-dimethylaminopyridine (1577.6 mg, 12.9 mmol, 0.8 equiv.) were added, and the reaction mixture stirred for 48 h at RT to yield monoester S8 (1381.0 mg, 67%). ^{1}H NMR (500 MHz, DMSO-*D*$_6$): δ 12.16 (s, 1H), 3.55 (s, 3H), 2.48 – 2.41 (m, 4H) ppm. ^{13}C NMR (125 MHz, DMSO-*D*$_6$): δ 173.4, 172.6, 51.4, 28.6, 28.5 ppm. IR: 2932.26, 1732.32, 1653.16, 1436.81, 1170.72, 942.87. mp: 56.8 ± 0.6 °C. MS (m/z) calcd for $C_5H_8NaO_4$: 155.11; found: 154.93 [M+Na$^+$].

4-oxo-4-((1,2,3,4-tetrahydronaphthalen-1-yl)oxy)butanoic acid (S9)

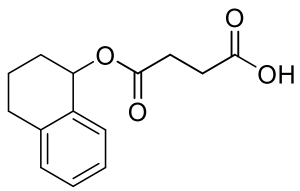

According to GP1, 1,2,3,4-tetrahydronaphthalen-1-ol (500 mg, 3.37 mmol), and *N,N*-diisopropylethylamine (881 µL, 5.06 mmol, 1.50 equiv.) were stirred in CH_2Cl_2 (4 mL) at RT. After 2 h, succinic anhydride (506.4 mg, 5.06 mmol, 1.5 equiv.) and 4-dimethylaminopyridine (618.3 mg, 5.06 mmol, 1.50 equiv.) were added, and the reaction mixture stirred for 48 h at RT to yield monoester S11 (700.6 mg, 84%). ^{1}H NMR (500 MHz, DMSO): δ12.23 (s, 1H), 7.26 – 7.09 (m, 4H), 5.90 – 5.84 (m, 1H), 2.84 – 2.61 (m, 2H), 2.55 – 2.43 (m, 5H), 1.97 – 1.71 (m, 4H).^{13}C NMR (125 MHz, DMSO): δ 173.4, 171.8, 137.6, 134.4, 129.0, 128.8, 127.9, 125.9, 69.4, 29.1, 28.8, 28.6, 28.3, 18.5. mp: 91.8 ± 0.2 °C. MS (m/z) calcd for $C_{14}H_{16}NaO_4$: 271.27; found: 270.99 [M+Na$^+$].

Purification of (1E,4Z,6E)-7-(4-(benzyloxy)-3-methoxyphenyl)-5-hydroxy-1-(4-hydroxy-3-methoxyphenyl)hepta-1,4,6-trien-3-one, (2)

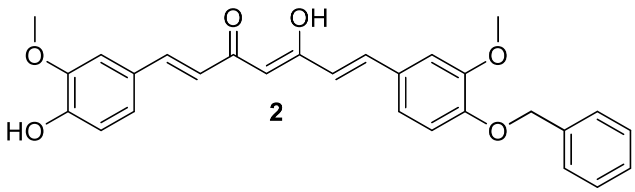

In our previous publication [1], the compound **2** was synthesized. In this investigation, the product was re-purified by column chromatography [hexane/EtOAc, silica gel] with the gradient system of *n*-hexane to ethyl acetate in 20 min at 2 mL/min to give the desired product (3.0 mg). LRMS (m/z) calcd for $C_{28}H_{26}O_6$: 458.17; found: 459.98 [$M+H^+$].

General procedure (GP2) for the synthesis of dialkylcurcumin and monoalkylcurcumin (3-15).

A borosilicate glass tube (16 mL) fitted with screw-caps equipped with magnetic stirrer was flushed with argon and charged with succinate S1 (31.3 mg, 0.12 mmol, 1.0 equiv.), 4-dimethylaminopyridine (16.1 mg, 0.13 mmol, 1.0 equiv.), curcumin (50.5 mg, 0.13 mmol), and dichloromethane (4 mL) were combined and stirred at 0 °C for 10 min. After, N-(3-Dimethylaminopropyl)-N′-ethylcarbodiimide hydrochloride (26.1 mg, 0.14 mmol, 1.0 equiv.) was added and stirred at RT for 24 h. The reaction was extracted with with EtOAc and water (3 x 20 mL). The organic phases were concentrated under reduced pressure, and the remaining material was purified by HPLC to obtain the curcumin derivative (Figure supplementary 2).

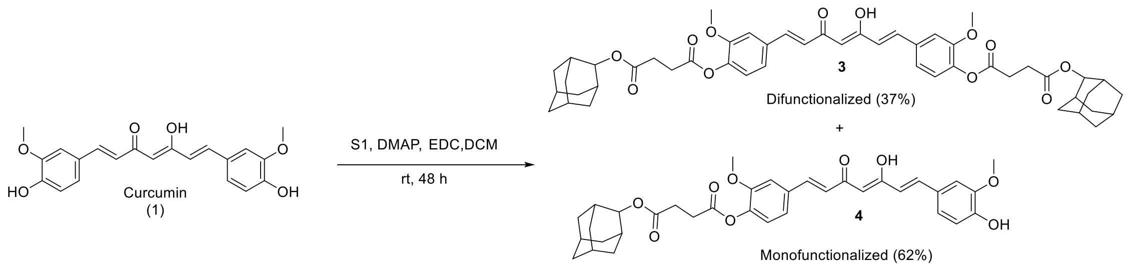

Figure Supplementary 2. Methodology of synthesis of Curcumin derivatives (3-15)

Di((1r,3r,5r,7r)-adamantan-2-yl) *O,O′*-(((1*E*,3*Z*,6*E*)-3-hydroxy-5-oxohepta-1,3,6-triene-1,7-diyl)bis(2-methoxy-4,1-phenylene)) disuccinate (3) and (1r,3r,5r,7r)-adamantan-2-yl (4-((1*E*,4*Z*,6*E*)-5-hydroxy-7-(4-hydroxy-3-methoxyphenyl)-3-oxohepta-1,4,6-trien-1-yl)-2-methoxyphenyl) succinate (4).

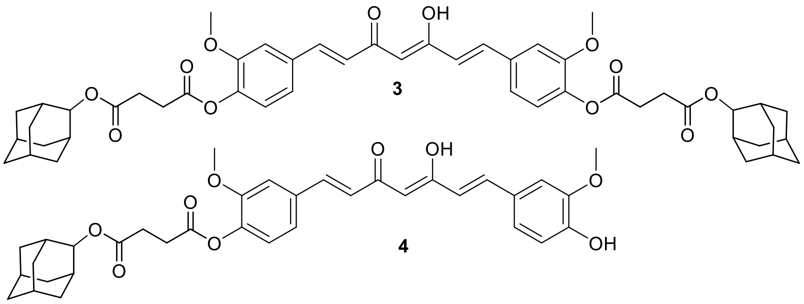

According to GP2, succinate S1 (31.3 mg, 0.12 mmol, 1.0 equiv.), 4-dimethylaminopyridine (16.1 mg, 0.13 mmol, 1.0 equiv.), curcumin (50.5 mg, 0.13 mmol), and dichloromethane (4 mL) were combined and stirred at 0 °C for 10 min. After, N-(3-Dimethylaminopropyl)-N′-ethylcarbodiimide hydrochloride (26.1 mg, 0.14 mmol, 1.0 equiv.) was added and stirred at RT for 24 h. The crude product was purified by HPLC to obtain diester 3 (21.0 mg, 37%) and monoester 4 (25.5 mg, 62%). Compound 3: ^{1}H NMR (500 MHz, $CDCl_3$): δ 7.62 (d, *J* = 15.9 Hz, 2H), 7.17 – 7.06 (m, 6H), 6.57 (d, *J* = 15.8 Hz, 2H), 5.89 – 5.83 (m, 1H), 4.97 (s, 2H), 3.87 (s, 6H), 2.96 (t, *J* = 7.4 Hz, 4H), 2.79 (t, *J* = 6.9 Hz, 4H), 2.01 (s, 8H), 1.90 – 1.66 (m, 20H). ^{13}C NMR (125 MHz, $CDCl_3$): δ 183.2, 171.5, 170.5, 151.5, 141.3, 140.1, 134.1, 124.4, 123.4, 121.2, 111.5, 102.0, 77.7, 56.1, 37.5, 36.4, 31.9, 31.9, 29.8, 29.2, 27.3, 27.1.ppm. IR: 2909.0, 2854.6, 1765.5, 1729.4, 1630.3, 1508.2, 1416.5, 1256.1, 1129.7 cm^{-1}.MS (m/z) calcd for $C_{49}H_{56}NaO_{12}$: 859.37; found: 859.6 $[M+Na^+]$. Compound 4: ^{1}H NMR (500 MHz, $CDCl_3$): δ 7.60 (dd, *J* = 15.8, 5.7 Hz, 2H), 7.19 – 7.00 (m, 6H), 6.94 (d, *J* = 8.2 Hz, 1H), 6.52 (dd, *J* = 27.3, 15.8 Hz, 2H), 5.83 (s, 1H), 5.01 – 4.93 (m, 1H), 3.95 (s, 3H), 3.87 (s, 3H), 2.96 (t, *J* = 7.0 Hz, 2H), 2.79 (t, *J* = 7.0 Hz, 2H), 2.00 (s, 4H), 1.87 – 1.71 (m, 10H). ^{13}C NMR (125 MHz, $CDCl_3$): δ 184.6, 181.9, 171.5, 170.6, 151.4, 148.1, 146.9, 141.3, 141.2, 139.6, 134.2, 127.7, 124.3, 123.4, 123.2, 121.9, 121.1, 115.0, 111.5, 109.7, 101.7, 77.7, 77.4, 56.1, 56.0, 37.5, 36.4, 31.9, 31.9, 29.8, 29.2, 27.3, 27.1 ppm. IR: 3403.0, 2908.8, 2854.7, 1726.1, 1626.5, 1587.5, 1509.6, 1267.0, 1129.0 cm^{-1}.MS (m/z) calcd for $C_{35}H_{38}NaO_9$: 625.24; found: 625.4 $[M+Na^+]$.

(9,10-dioxo-9,10-dihydroanthracen-2-yl)methyl (4-((1*E*,4*Z*,6*E*)-5-hydroxy-7-(4-hydroxy-3-methoxyphenyl)-3-oxohepta-1,4,6-trien-1-yl)-2-methoxyphenyl) succinate (5)

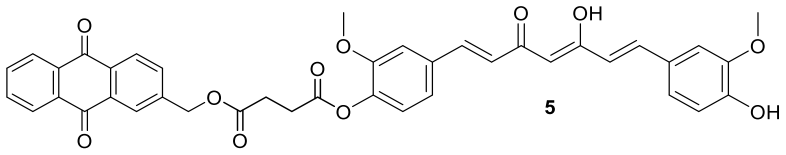

According to GP2, succinate S2 (41.4 mg, 0.12 mmol, 0.9 equiv), 4-dimethylaminopyridine (15.1 mg, 0.12 mmol, 1.0 equiv.), curcumin (50.1 mg, 0.13 mmol), and dichloromethane (4 mL) were combined and stirred at 0 °C for 10 min. After, N-(3-Dimethylaminopropyl)-N′-ethylcarbodiimide hydrochloride (24.9 mg, 0.13 mmol, 1.0 equiv.) was added and stirred at RT for 24 h. The crude product was purified by HPLC to obtain monoester 5 (11.3 mg, 13%). Compound 5: ^{1}H NMR (500 MHz, $CDCl_3$): δ 8.31 – 8.26 (m, 4H), 7.84 – 7.76 (m, 4H), 7.56 (dd, *J* = 50.9, 15.8 Hz, 2H), 7.14 – 7.04 (m, 4H), 6.96 (dd, *J* = 23.4, 8.2 Hz, 2H), 6.49 (dd, *J* = 15.8, 5.2 Hz, 2H), 5.81 (s, 1H), 5.32 (s, 2H), 3.95 (s, 3H), 3.84 (s, 3H), 3.00 – 2.96 (m, 2H), 2.89 – 2.85 (m, 2H) ppm. ^{13}C NMR (125 MHz, $CDCl_3$): 29.1, 29.3, 56.0, 56.1, 65.6, 101.7, 109.7, 111.5, 115.0, 126.4, 127.40, 127.43, 127.9, 133.3, 134.4, 139.4, 141.0, 141.3, 142.5, 146.9, 148.1, 151.3, 170.2, 171.9, 181.8, 182.9, 183.0, 184.7 ppm. IR: 3046.5, 2925.0, 2849.9, 1757.0, 1731.0, 1671.7, 1588.7, 1511.3, 1444.4, 1294.6, 1203.3, 1136.8 cm^{-1} MS (m/z) calcd for $C_{40}H_{32}O_{11}$: 688.6; found: 689.4 [$M+H^{+}$].

Benzhydryl (4-((1*E*,4*Z*,6*E*)-5-hydroxy-7-(4-hydroxy-3-methoxyphenyl)-3-oxohepta-1,4,6-trien-1-yl)-2-methoxyphenyl) succinate (6).

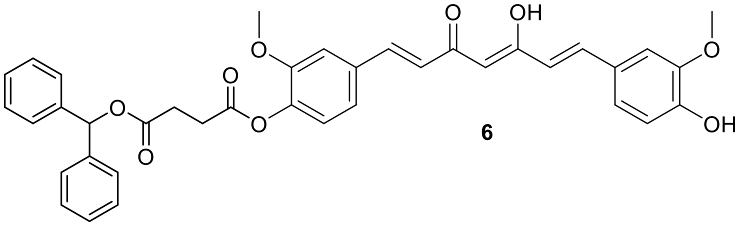

According to GP2, succinate S3 (135.88 mg, 0.48 mmol, 1.2 equiv), 4-dimethylaminopyridine (65.4 mg, 0.54 mmol, 1.3 equiv.), curcumin (152.4 mg, 0.41 mmol), and dichloromethane (4 mL) were combined and stirred at 0 °C for 10 min. After, N-(3-Dimethylaminopropyl)-N′-ethylcarbodiimide hydrochloride (84.87 mg, 0.44 mmol, 1.1 equiv.) was added and stirred at RT for 24 h. The crude product was purified by column chromatography on silica gel to obtain monoester 6 (37.7 mg, 14%). Compound 6: ^{1}H NMR (500 MHz, $CDCl_3$): δ 7.59 (dd, *J* = 15.8, 9.1 Hz, 2H), 7.35 – 7.30 (m, 10H), 7.13 – 7.03 (m, 4H), 6.93 (dd, *J* = 8.2, 4.6 Hz, 2H), 6.91 (s, 1H), 6.51 (dd, *J* = 24.4, 15.8 Hz, 2H), 5.82 (s, 1H), 3.94 (s, 3H), 3.79 (s, 3H), 2.97 – 2.92 (m, 2H), 2.90 – 2.86 (m, 2H) ppm. ^{13}C NMR (125 MHz, $CDCl_3$): δ 184.7, 181.9, 171.2, 170.3, 151.4, 148.1, 146.9, 141.3, 141.1, 140.1, 139.6, 134.2, 128.7, 128.1, 127.2, 124.3, 123.4, 123.2, 121.9, 121.1, 115.0, 111.4, 109.7, 101.7, 77.6, 77.4, 56.1, 56.0, 29.6, 29.1 ppm. IR: 3511.7, 2922.4, 2850.8, 1740.0, 1624.7, 1586.0, 1510.1, 1302.8, 1261.5, 1121.9 cm^{-1} MS (m/z) calcd for $C_{38}H_{34}NaO_9$: 657.21; found: 657.4 [M+Na^{+}].

Bis(cyclohexyl(phenyl)methyl) *O,O′*-(((1*E*,3*Z*,6*E*)-3-hydroxy-5-oxohepta-1,3,6-triene-1,7-diyl)bis(2-methoxy-4,1-phenylene)) disuccinate (7) and cyclohexyl(phenyl)methyl (4-((1*E*,4*Z*,6*E*)-5-hydroxy-7-(4-hydroxy-3-methoxyphenyl)-3-oxohepta-1,4,6-trien-1-yl)-2-methoxyphenyl) succinate (8).

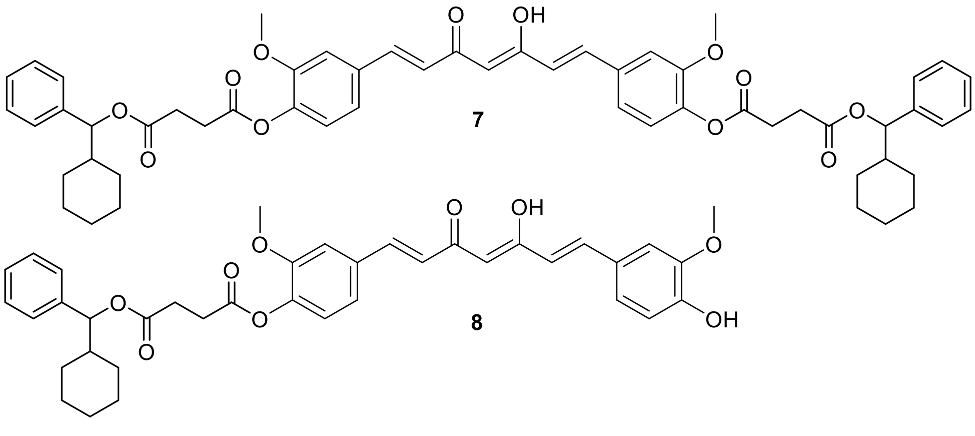

According to GP2, succinate S4 (114.8 mg, 0.40 mmol, 1.0 equiv), 4-dimethylaminopyridine (89.7 mg, 0.73 mmol, 1.8 equiv.), curcumin (150.3 mg, 0.40 mmol), and dichloromethane (4 mL) were combined and stirred at 0 °C for 10 min. After, N-(3-Dimethylaminopropyl)-N′-ethylcarbodiimide hydrochloride (92.8 mg, 0.48 mmol, 1.2 equiv.) was added and stirred at RT for 24 h. The crude product was purified by column chromatography on silica gel to obtain diester 7 (29.6 mg, 16%) and monoester 8 (59.2 mg, 45%). Compound 7: ^{1}H NMR (500 MHz, $CDCl_3$): δ 7.61 (d, *J* = 15.8 Hz, 2H), 7.33 – 7.27 (m, 10H), 7.17 – 7.09 (m, 4H), 6.99 (d, *J* = 8.1 Hz, 2H), 6.56 (d, *J* = 15.8 Hz, 2H), 5.52 (d, *J* = 8.0 Hz, 2H), 3.83 (s, 6H), 3.00 – 2.66 (m, 10H), 1.88 – 1.70 (m, 8H), 1.24 – 0.90 (m, 12H) ppm. ^{13}C NMR (125 MHz, $CDCl_3$): δ 183.2, 171.4, 170.3, 151.4, 141.3, 140.1, 139.6, 134.1, 128.3, 128.3, 127.9, 127.2, 127.2, 124.4, 123.4, 121.2, 111.5, 102.0, 81.0, 56.0, 43.1, 29.5, 29.1, 29.1, 26.3, 26.0, 25.9 ppm. IR: 2927.4, 2852.0, 1763.4, 1732.1, 1629.0, 1506.8, 1415.7, 1253.7, 1122.0 cm^{-1}. MS (m/z) calcd for $C_{55}H_{60}NaO_{12}$: 935.40; found: 935.8 [M+Na$^+$]. Compound 8: ^{1}H NMR (500 MHz, $CDCl_3$): δ 7.59 (dd, *J* = 15.8, 12.5 Hz, 2H), 7.35 – 7.26 (m, 5H), 7.14 – 7.02 (m, 4H), 6.95 (dd, *J* = 26.7, 8.1 Hz, 2H), 6.51 (dd, *J* = 22.6, 15.8 Hz, 2H), 5.82 (s, 1H), 5.52 (d, *J* = 8.0 Hz, 1H), 3.92 (d, *J* = 0.9 Hz, 3H), 3.82 (s, 3H), 3.01 – 2.63 (m, 5H), 1.93 – 1.66 (m, 4H), 1.22 – 0.86 (m, 6H) ppm. ^{13}C NMR (125 MHz, $CDCl_3$): δ 184.6, 181.9, 171.4, 170.4, 151.4, 148.1, 146.9, 141.3, 141.1, 139.6, 139.5, 134.2, 128.3, 127.9, 127.2, 115.0, 111.4, 109.7, 101.7, 81.0, 56.0, 56.0, 43.0, 29.5, 29.1, 29.1, 29.0, 26.3, 25.9, 25.9 ppm. IR: 3430.5, 2930.5, 2851.9, 1763.9, 1733.4, 1627.0, 1587.9, 1510.0, 1267.5, 1130.9 cm^{-1}. MS (m/z) calcd for $C_{38}H_{40}NaO_9$: 663.26; found: 663.5 [M+Na$^+$].

Di(9*H*-fluoren-9-yl) *O,O′*-(((1*E*,3*Z*,6*E*)-3-hydroxy-5-oxohepta-1,3,6-triene-1,7-diyl)bis(2-methoxy-4,1-phenylene)) disuccinate (9) and 9*H*-fluoren-9-yl (4-((1*E*,4*Z*,6*E*)-5-hydroxy-7-(4-hydroxy-3-methoxyphenyl)-3-oxohepta-1,4,6-trien-1-yl)-2-methoxyphenyl) succinate (10).

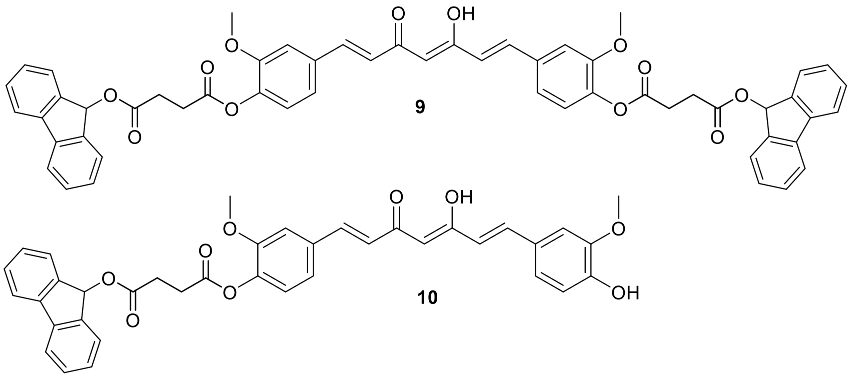

According to GP2, succinate S5 (34.5 mg, 0.12 mmol, 0.9 equiv), 4-dimethylaminopyridine (15.7 mg, 0.13 mmol, 1.0 equiv.), curcumin (50.0 mg, 0.13 mmol), and dichloromethane (4 mL) were combined and stirred at 0 °C for 10 min. After, N-(3-Dimethylaminopropyl)-N′-ethylcarbodiimide hydrochloride (24.2 mg, 0.13 mmol, 1.0 equiv.) was added and stirred at RT for 24 h. The crude product was purified by HPLC to obtain diester 9 (4.4 mg, 4%) and monoester 10 (22.3 mg, 26%). Compound 9: ^{13}C NMR (125 MHz, $CDCl_3$): δ 183.2, 172.9, 170.4, 151.4, 148.0, 146.9, 141.9, 141.1, 134.1, 129.7, 129.7, 128.0, 128.0, 126.1, 126.0, 120.2, 120.2, 114.9, 111.5, 109.7, 75.6, 56.1, 56.0, 52.1, 29.6, 29.5, 29.2, 29.1 ppm. IR: 2958.3, 2923.4, 2852.1, 1760.7, 1730.9, 1627.7, 1587.8, 1505.3, 1451.3, 1249.6, 1117.3 cm^{-1}. MS (m/z) calcd for $C_{55}H_{44}NaO_{12}$: 919.27; found: 919.30 [M+Na$^+$]. Compound 10: ^{13}C NMR (125 MHz, $CDCl_3$): δ 184.7, 181.9, 172.9, 170.4, 151.4, 148.1, 147.9, 146.9, 146.9, 141.9, 141.1, 129.7, 128.0, 126.1, 123.0, 121.9, 121.8, 120.2, 115.0, 114.9, 109.7, 101.8, 75.6, 56.1, 56.0, 52.1, 29.6, 29.2 ppm. IR: 3426.6, 2959.8, 2926.3, 2850.6, 1760.2, 1730.6, 1625.1, 1585.5, 1507.1, 1450.4, 1250.8, 1117.8 cm^{-1}. MS (m/z) calcd for $C_{38}H_{32}NaO_9$: 655.19; found: 655.05 [M+Na$^+$].

Bis(2,3-dihydro-1H-inden-2-yl) *O,O′*-(((1*E*,3*Z*,6*E*)-3-hydroxy-5-oxohepta-1,3,6-triene-1,7-diyl)bis(2-methoxy-4,1-phenylene)) disuccinate (11).

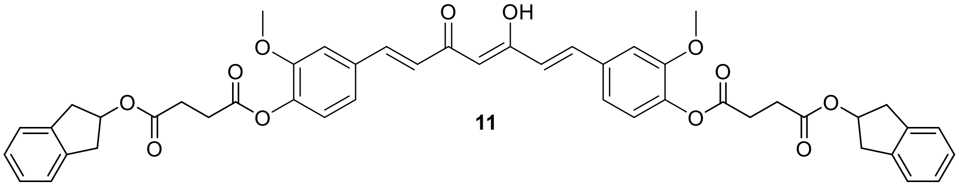

According to GP2, succinate S6 (100.2 mg, 0.43 mmol, 3.1 equiv), 4-dimethylaminopyridine (23.9 mg, 0.20 mmol, 1.2 equiv.), curcumin (50.1 mg, 0.14 mmol), and pyridine (2.5 mL) were combined and stirred at 0 °C for 10 min. After, N-(3-Dimethylaminopropyl)-N′-ethylcarbodiimide hydrochloride (152.1 mg, 0.79 mmol, 5.8 equiv.) was added and stirred at RT for 24 h. The crude product was purified by HPLC to obtain diester 11 (17.6 mg, 16 %). Compound 11: ^{1}H NMR (500 MHz, $CDCl_3$): δ 7.62 (d, *J* = 15.8 Hz, 2H), 7.24 – 7.19 (m, 8H), 7.16 – 7.10 (m, 4H), 7.01 (d, *J* = 8.2 Hz, 2H), 6.57 (d, *J* = 15.8 Hz, 2H), 5.87 (s, 1H), 5.61 – 5.57 (m, 2H), 3.85 (s, 6H), 3.33 (dd, *J* = 16.9, 6.4 Hz, 4H), 3.03 (dd, *J* = 17.0, 2.9 Hz, 4H), 2.92 (t, *J* = 6.8 Hz, 4H), 2.71 (t, *J* = 6.8 Hz, 4H). ^{13}C NMR (125 MHz, $CDCl_3$): δ183.2, 172.1, 170.5, 151.4, 141.2, 140.5, 140.1, 134.1, 126.9, 124.8, 124.8, 124.8, 124.3, 123.4, 121.2, 111.5, 102.0, 75.9, 56.0, 39.7, 29.5, 29.0 ppm. IR: 2922.7, 2851.5, 1760.9, 1727.1, 1505.8, 1415.1, 1252.5, 1117.6 cm^{-1}. MS (m/z) calcd for $C_{47}H_{44}NaO_{12}$: 823.27; found: 823.5 [$M+Na^{+}$].

O,O′-(((1E,3Z,6E)-3-hydroxy-5-oxohepta-1,3,6-triene-1,7-diyl)bis(2-methoxy-4,1-phenylene)) bis((1R,2S,5R)-2-isopropyl-5-methylcyclohexyl) disuccinate (12) and 4-((1E,4Z,6E)-5-hydroxy-7-(4-hydroxy-3-methoxyphenyl)-3-oxohepta-1,4,6-trien-1-yl)-2-methoxyphenyl((1R,2S,5R)-2-isopropyl-5-methylcyclohexyl) succinate (13).

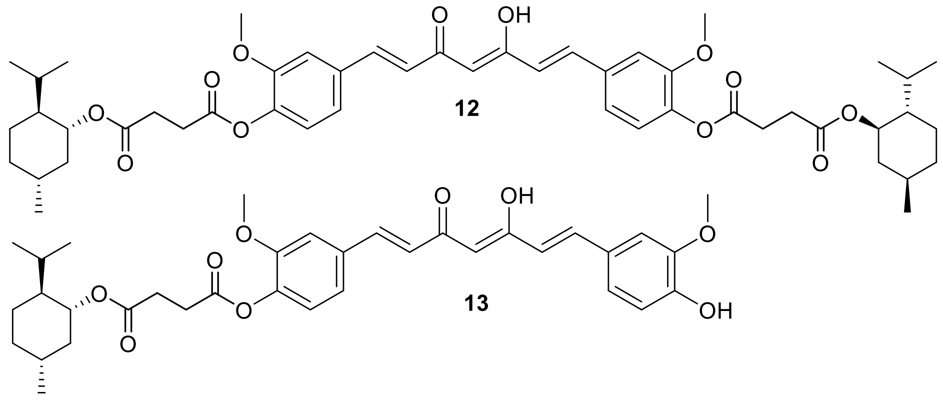

According to GP2, succinate S7 (33.1 mg, 0.13 mmol, 1.0 equiv), 4-dimethylaminopyridine (17.5 mg, 0.14 mmol, 1.1 equiv.), curcumin (50.3 mg, 0.13 mmol), and dichloromethane (4 mL) were combined and stirred at 0 °C for 10 min. After, N-(3-Dimethylaminopropyl)-N′-ethylcarbodiimide hydrochloride (23.7 mg, 0.12 mmol, 0.9 equiv.) was added and stirred at RT for 24 h. The crude product was purified by HPLC to obtain diester 12 (5.3 mg, 9%) and monoester 13 (23.6 mg, 57%). Compound 12: ^{1}H NMR (500 MHz, $CDCl_3$): δ 7.62 (d, *J* = 15.8 Hz, 2H), 7.17 – 7.06 (m, 6H), 6.57 (d, *J* = 15.8 Hz, 2H), 5.86 (s, 1H), 4.75 – 4.70 (m, 2H), 3.87 (s, 6H), 2.93 (t, *J* = 7.2 Hz, 4H), 2.75 – 2.72 (m, 4H), 1.99 (d, *J* = 11.5 Hz, 2H), 1.88 – 1.84 (m, 2H), 1.68 (dd, *J* = 8.9, 5.4 Hz, 4H), 1.46 – 1.35 (m, 4H), 1.03 – 0.96 (m, 4H), 0.89 – 0.87 (m, 12H), 0.74 (d, *J* = 6.9 Hz, 6H). ^{13}C NMR (125 MHz, $CDCl_3$): δ 183.2, 171.7, 170.5, 151.5, 141.3, 140.1, 134.1, 124.4, 123.4, 121.2, 111.6, 102.0, 74.9, 56.1, 47.1, 41.0, 34.3, 31.5, 29.6, 29.2, 26.4, 23.5, 22.2, 20.9, 16.4 ppm. IR: 2954.7, 2929.4, 2869.3, 1765.2, 1727.9, 1630.7, 1508.4, 1462.4, 1416.6, 1129.9 cm^{-1}. MS (m/z) calcd for $C_{49}H_{64}NaO_{12}$: 867.43; found: 867.46 [M+Na^{+}]. Compound 13: ^{1}H NMR (500 MHz, $CDCl_3$): δ 7.60 (dd, *J* = 15.8, 7.0 Hz, 2H), 7.18 – 7.01 (m, 6H), 6.93 (d, *J* = 8.2 Hz, 1H), 6.51 (dd, *J* = 26.9, 15.7 Hz, 2H), 5.82 (s, 1H), 4.79 – 4.65 (m, 1H), 3.94 (s, 3H), 3.86 (s, 3H), 2.96 – 2.90 (m, 2H), 2.78 – 2.69 (m, 2H), 2.02 – 1.96 (m, 1H), 1.86 (qd, *J* = 7.0, 2.7 Hz, 1H), 1.70 – 1.64 (m, 2H), 1.49 – 1.34 (m, 2H), 1.29 – 1.21 (m, 1H), 1.08 – 0.95 (m, 2H), 0.88 (t, *J* = 6.9 Hz, 6H), 0.74 (d, *J* = 7.0 Hz, 3H) ppm. ^{13}C NMR (125 MHz, $CDCl_3$): δ 184.7, 181.9, 171.7, 170.5, 151.4, 148.1, 146.9, 141.3, 141.2, 139.5, 134.2, 127.6, 124.3, 123.4, 123.2, 121.8, 121.1, 115.0, 111.5, 109.7, 101.7, 74.9, 56.1, 56.0, 47.1, 41.0, 34.3, 31.5, 29.6, 29.2, 26.3, 23.5, 22.1, 20.9, 16.4.ppm. IR: 2954.5, 2931.6, 2869.1, 1764.7, 1726.6, 1627.4, 1510.3, 1417.7, 1267.7, 1131.6 cm^{-1}. MS (m/z) calcd for $C_{35}H_{42}NaO_9$: 629.27; found: 629.17 [M+Na^{+}].

4-((1E,4Z,6E)-5-hydroxy-7-(4-hydroxy-3-methoxyphenyl)-3-oxohepta-1,4,6-trien-1-yl)-2-methoxyphenyl methyl succinate (14).

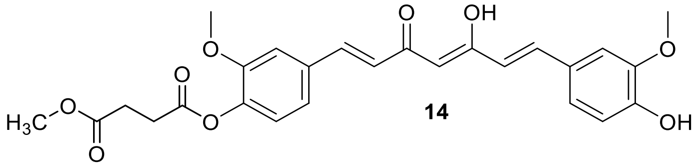

According to GP2, succinate S8 (60.4 mg, 0.46 mmol, 1.1 equiv), 4-dimethylaminopyridine (66.4 mg, 0.54 mmol, 1.3 equiv.), curcumin (151.6 mg, 0.41 mmol), and dichloromethane (4 mL) were combined and stirred at 0 °C for 10 min. After, N-(3-Dimethylaminopropyl)-N′-ethylcarbodiimide hydrochloride (87.9 mg, 0.46 mmol, 1.1 equiv.) was added and stirred at RT for 24 h. The crude product was purified by column chromatography on silica gel to obtain monoester 14 (22.3 mg, 11 %). ^{1}H NMR (500 MHz, $CDCl_3$): δ 7.65 – 7.52 (m, 2H), 7.16 – 7.00 (m, 5H), 6.92 (dd, *J* = 8.1, 1.8 Hz, 1H), 6.56 – 6.42 (m, 2H), 5.82 (s, 1H), 3.93 (s, 3H), 3.86 (s, 3H), 3.72 (s, 3H), 2.94 (t, *J* = 6.7 Hz, 2H), 2.76 (t, *J* = 6.9 Hz, 2H) ppm. ^{13}C NMR (125 MHz, $CDCl_3$): δ 184.7, 181.8, 172.6, 170.5, 151.4, 148.3, 148.1, 147.0, 147.0, 141.3, 141.1, 140.7, 139.5, 134.2, 127.6, 127.5, 124.3, 123.4, 123.2, 123.0, 121.7, 121.7, 121.1, 115.0, 115.0, 111.5, 109.8, 109.7, 106.5, 101.7, 56.0, 52.1, 29.0. IR: 3405.4, 2950.9, 2845.6, 1760.1, 1735.3, 1625.9, 1586.8, 1509.7, 1417.8, 1126.9 cm-1. MS (m/z) calcd for $C_{35}H_{34}NaO_9$: 621.21; found: 621.4 [$M+Na^+$].

4-((1E,4Z,6E)-5-hydroxy-7-(4-hydroxy-3-methoxyphenyl)-3-oxohepta-1,4,6-trien-1-yl)-2-methoxyphenyl (1,2,3,4-tetrahydronaphthalen-1-yl) succinate (15).

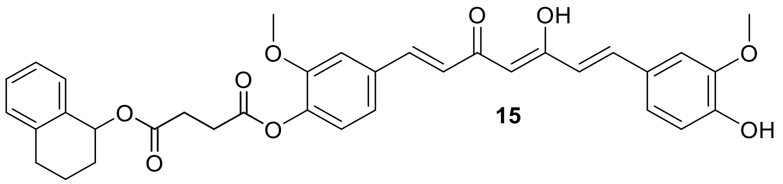

According to GP2, succinate S9 (95.91 mg, 0.39 mmol, 1.0 equiv), 4-dimethylaminopyridine (52.9 mg, 0.43 mmol, 1.1 equiv.), curcumin (150.47 mg, 0.41 mmol), and dichloromethane (4 mL) were combined and stirred at 0 °C for 10 min. After, N-(3-Dimethylaminopropyl)-N′-ethylcarbodiimide hydrochloride (76.9 mg, 0.40 mmol, 1.0 equiv.) was added and stirred at RT for 24 h. The crude product was purified by column chromatography on silica gel to obtain monoester 15 (10.6 mg, 4%). Compound 15: ^{1}H NMR (500 MHz, $CDCl_3$): δ 15.96 (s, 1H), 7.60 (dd, *J* = 15.8, 6.7 Hz, 2H), 7.24 – 7.09 (m, 7H), 7.05 (d, *J* = 1.9 Hz, 1H), 6.99 (d, *J* = 8.1 Hz, 1H), 6.94 (d, *J* = 8.2 Hz, 1H), 6.52 (dd, *J* = 26.8, 15.8 Hz, 2H), 6.05 (t, *J* = 4.5 Hz, 1H), 5.83 (s, 1H), 3.95 (s, 3H), 3.86 (s, 3H), 2.99 – 2.93 (m, 2H), 2.79 – 2.76 (m, 2H), 2.03 – 1.92 (m, 3H), 1.87 – 1.78 (m, 1H).^{13}C NMR (125 MHz, $CDCl_3$): δ 184.6, 181.9, 171.8, 170.5, 151.4, 148.1, 146.9, 141.3, 141.2, 139.5, 138.1, 134.4, 134.2, 129.7, 129.2, 128.3, 127.7, 126.3, 124.3, 123.4, 123.2, 121.9, 121.1, 115.0, 111.5, 109.7, 101.7, 70.7, 56.1, 56.0, 29.8, 29.2, 29.1, 18.9 ppm. IR: 3421.9, 2938.6, 2868.4, 2840.0, 1727.9, 1627.0, 1587.8, 1510.0, 1131.3 cm^{-1}. MS (m/z) calcd for $C_{35}H_{34}NaO_9$: 621.21; found: 621.4 [$M+Na^+$].

4-((adamantan-2-yl)oxy)-4-oxobutanoic acid (S1)

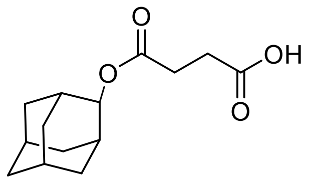

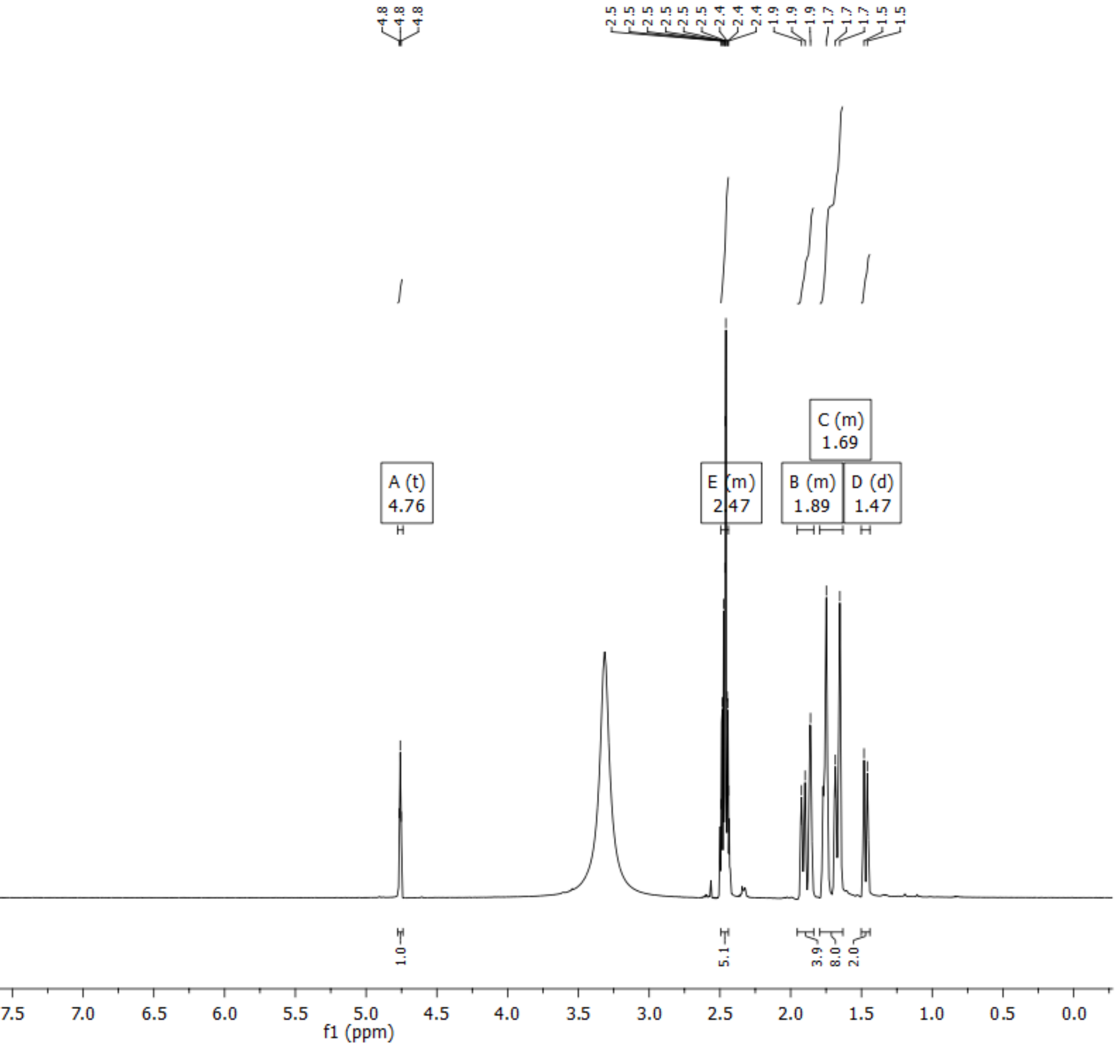

4-(((1r,3r,5r,7r)-adamantan-2-yl)oxy)-4-oxobutanoic acid (S1)

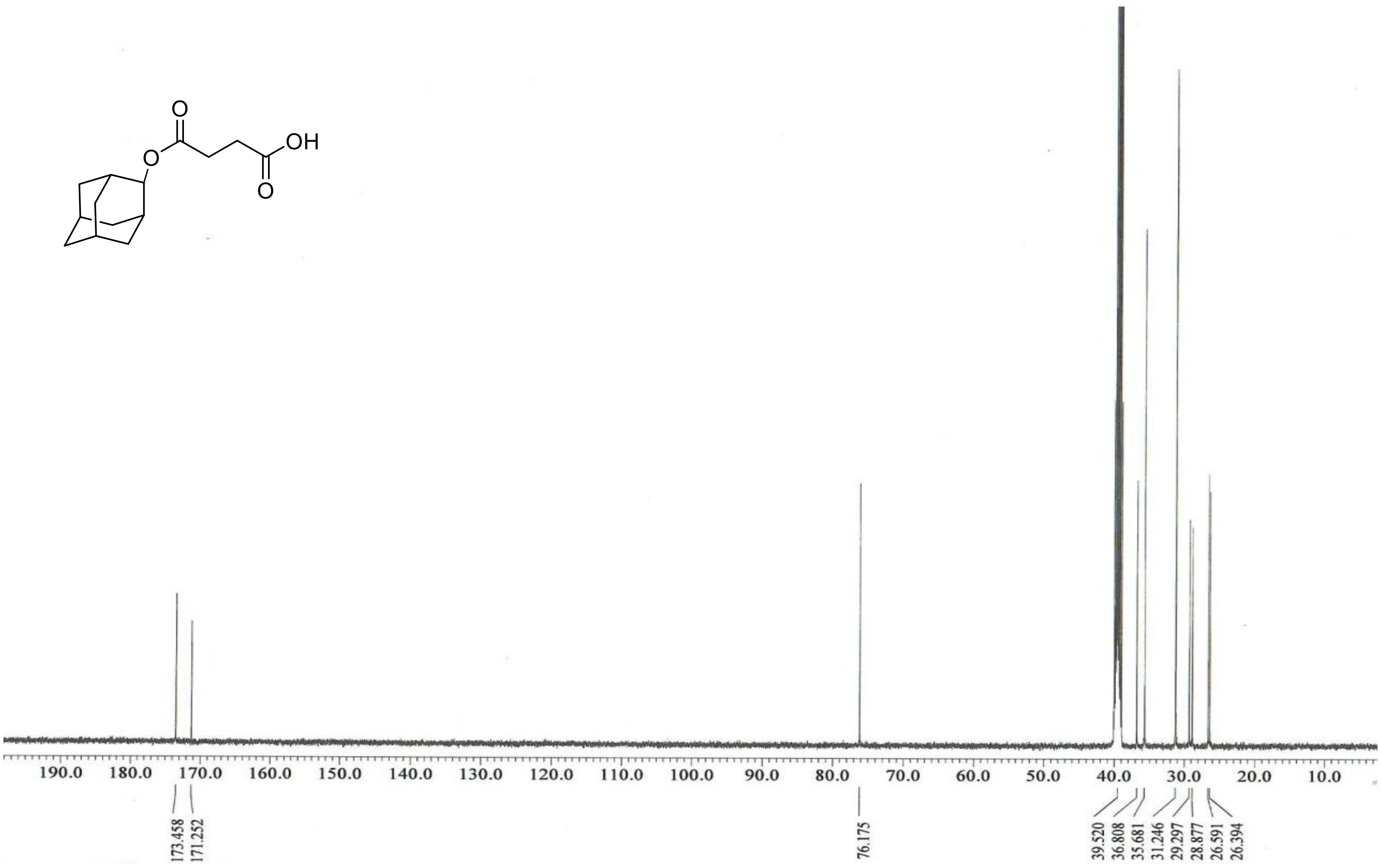

4-((9,10-dioxo-9,10-dihydroanthracen-2-yl)methoxy)-4-oxobutanoic acid (S2)

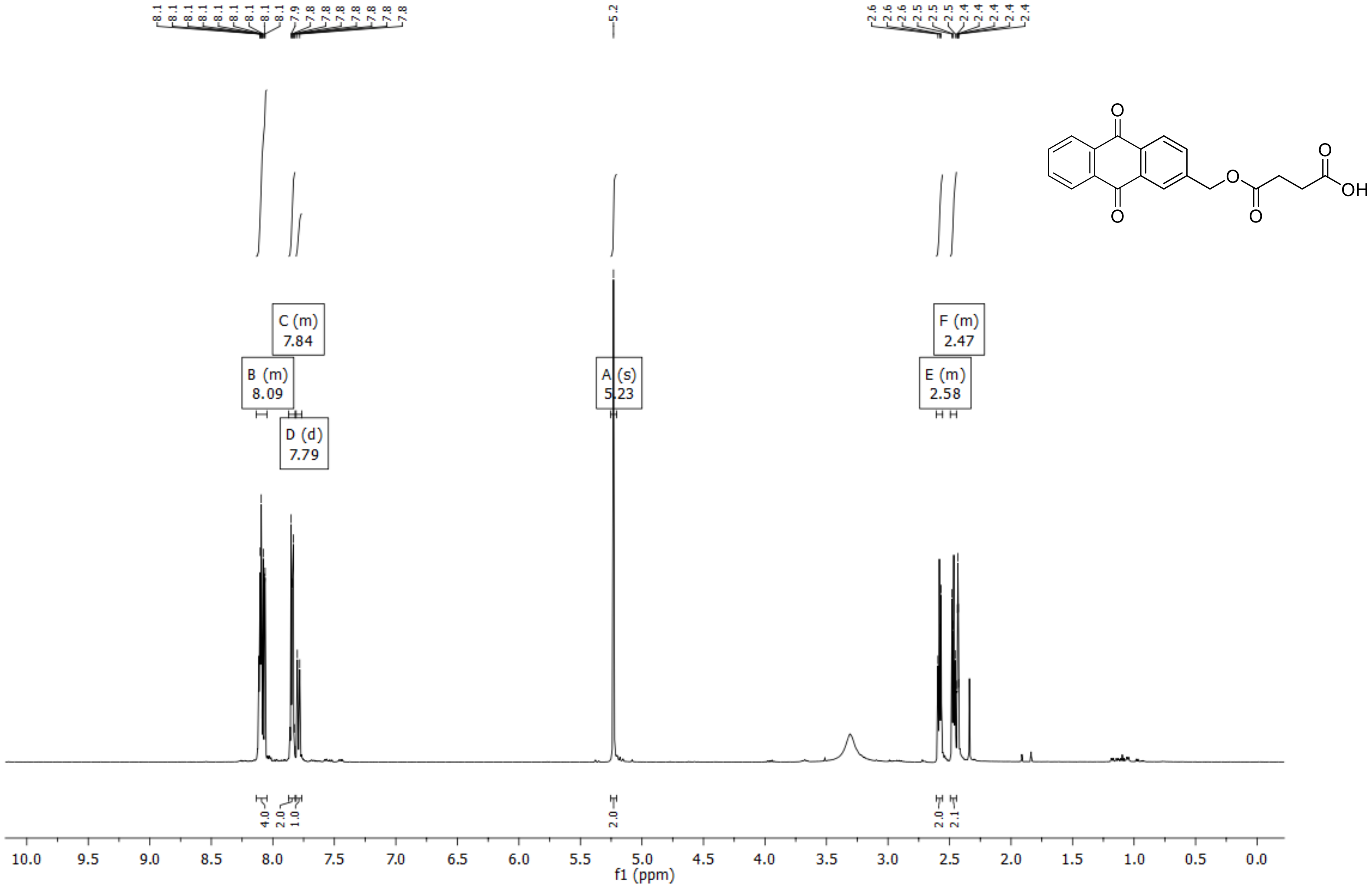

4-((9,10-dioxo-9,10-dihydroanthracen-2-yl)methoxy)-4-oxobutanoic acid (S2)

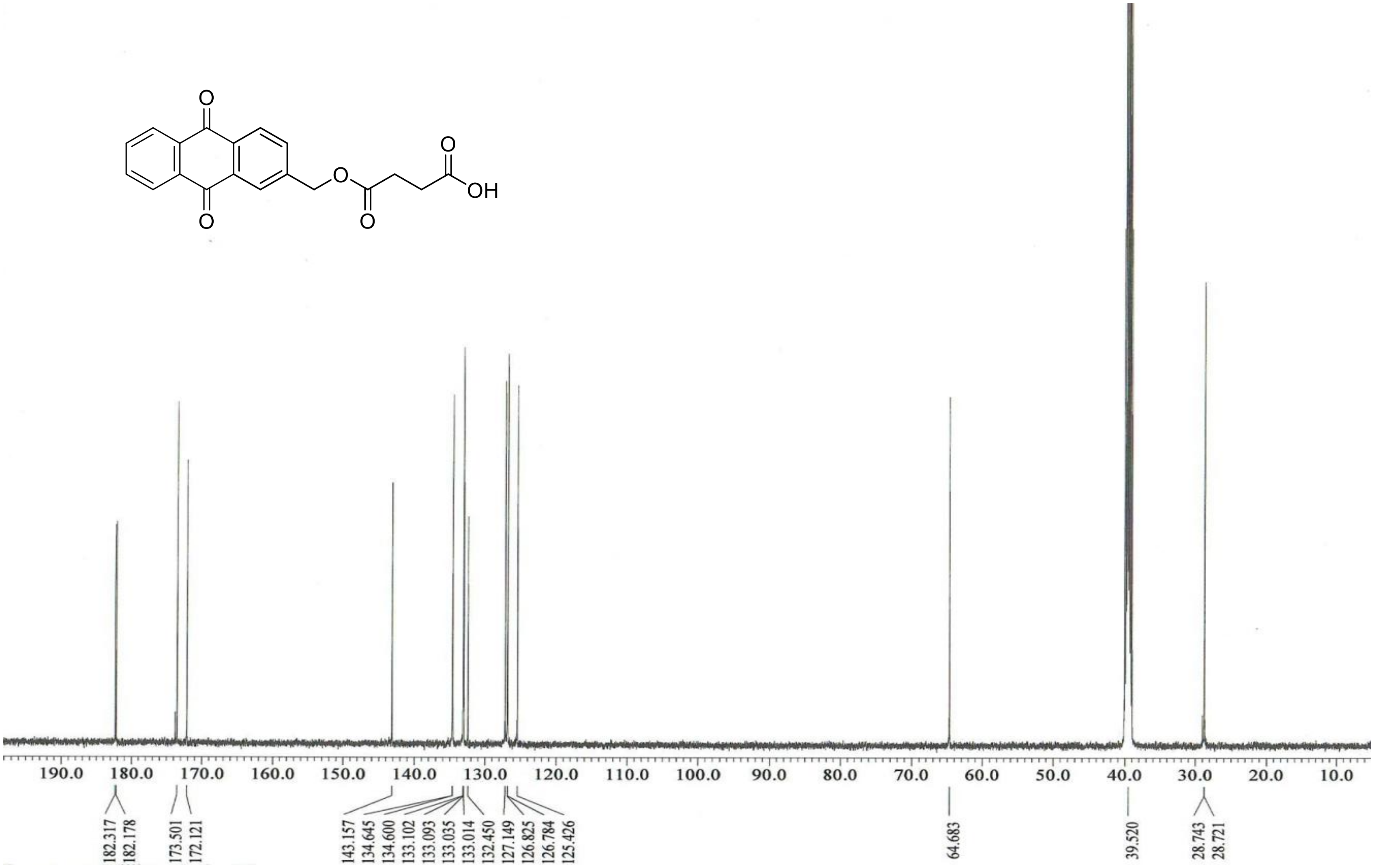

4-(benzhydryloxy)-4-oxobutanoic acid (S3)

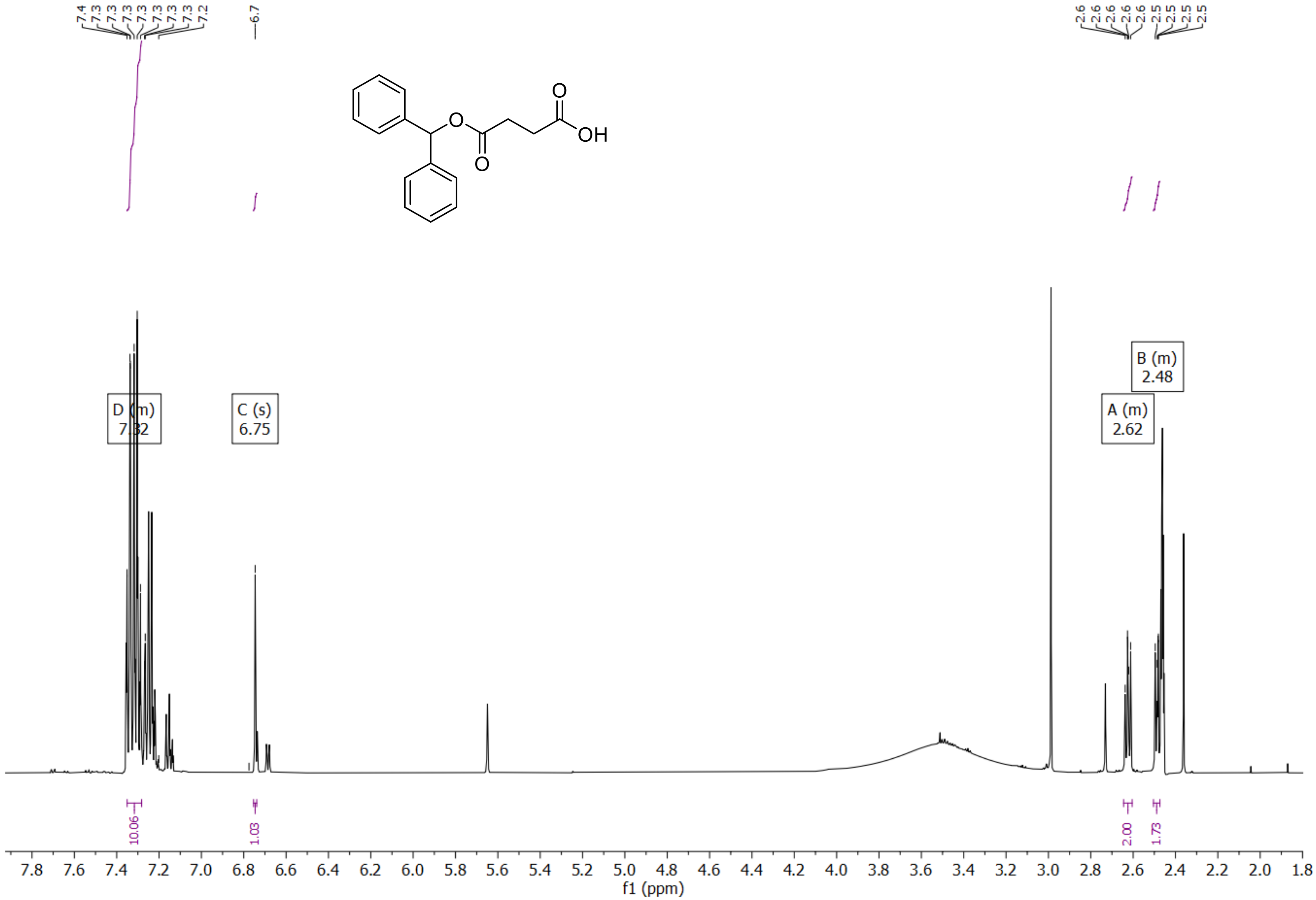

4-(benzhydryloxy)-4-oxobutanoic acid (S3)

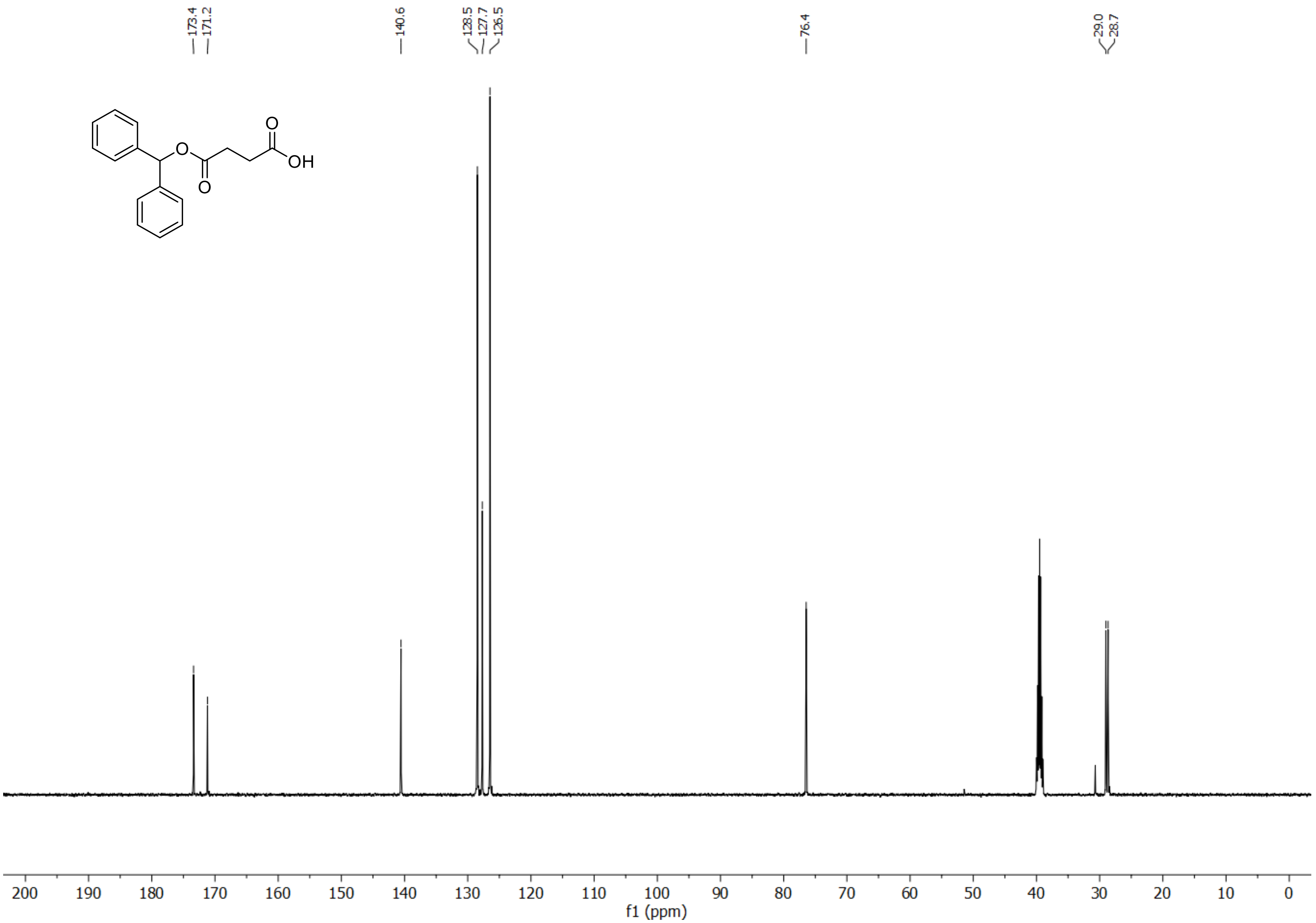

4-(cyclohexyl(phenyl)methoxy)-4-oxobutanoic acid (S4)

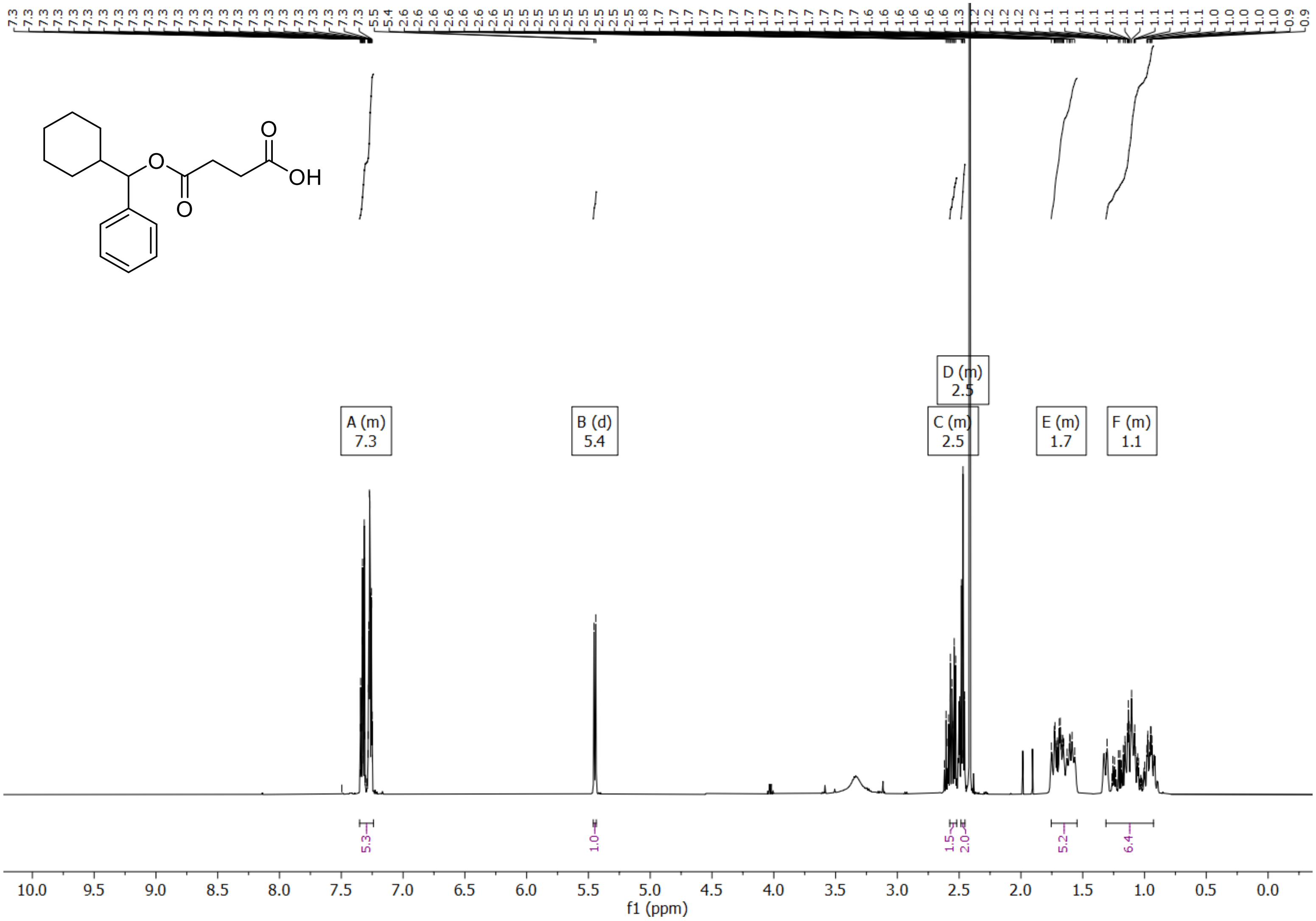

4-(cyclohexyl(phenyl)methoxy)-4-oxobutanoic acid (S4)

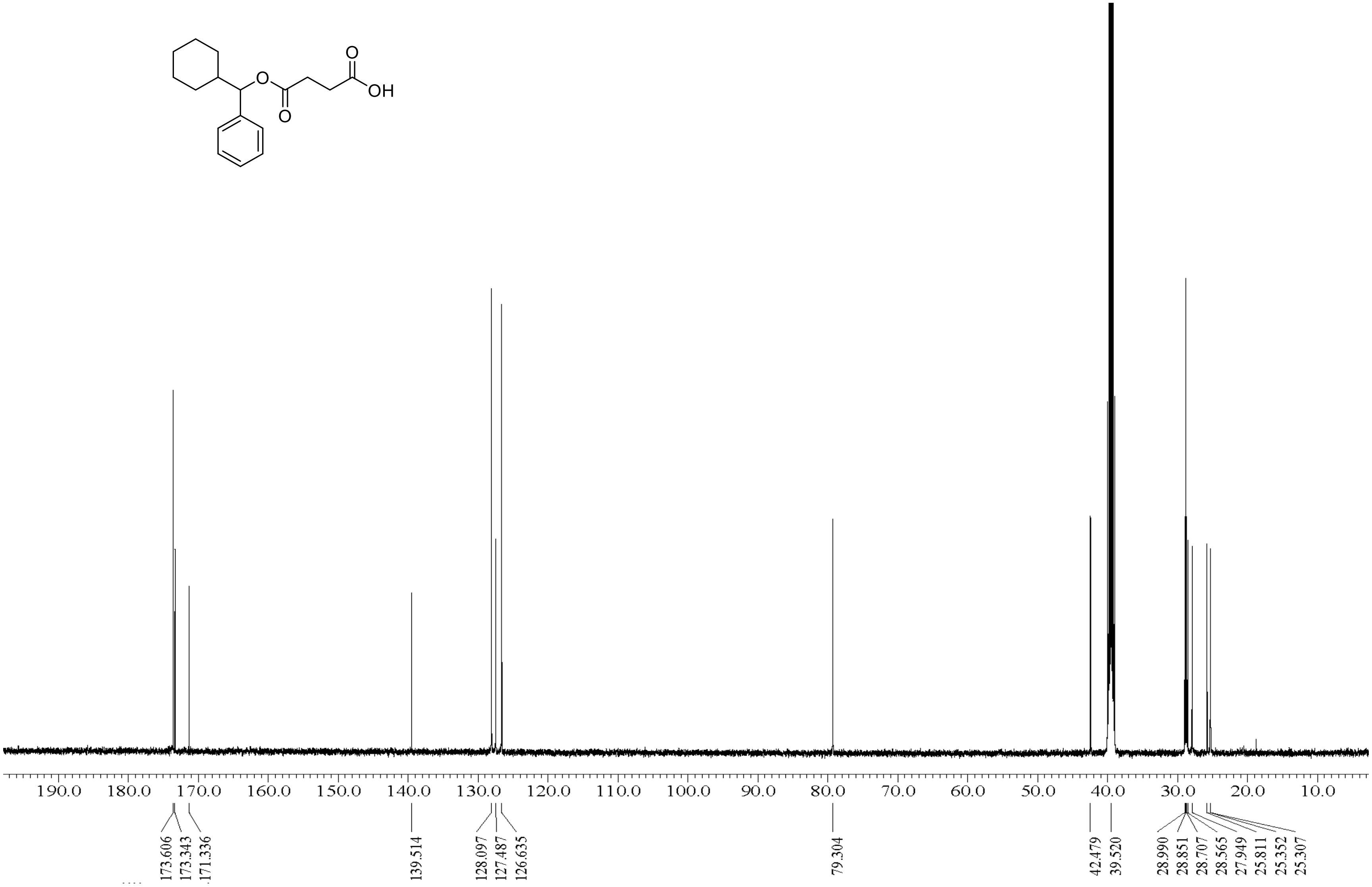

4-((9H-fluoren-9-yl)oxy)-4-oxobutanoic acid (S5)

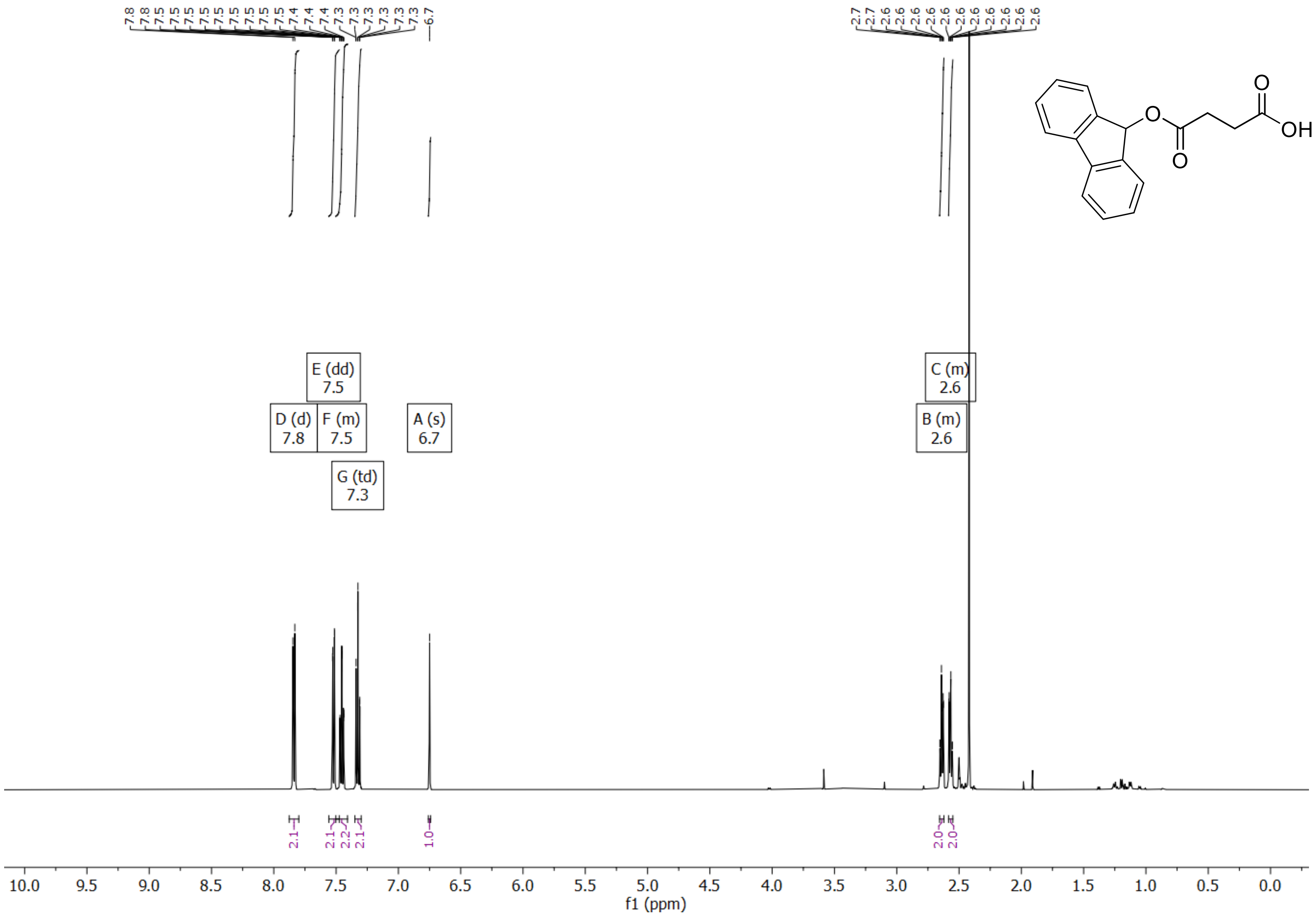

4-((9H-fluoren-9-yl)oxy)-4-oxobutanoic acid (S5)

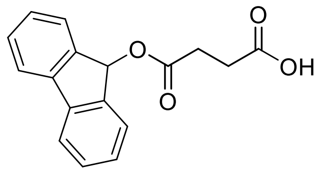

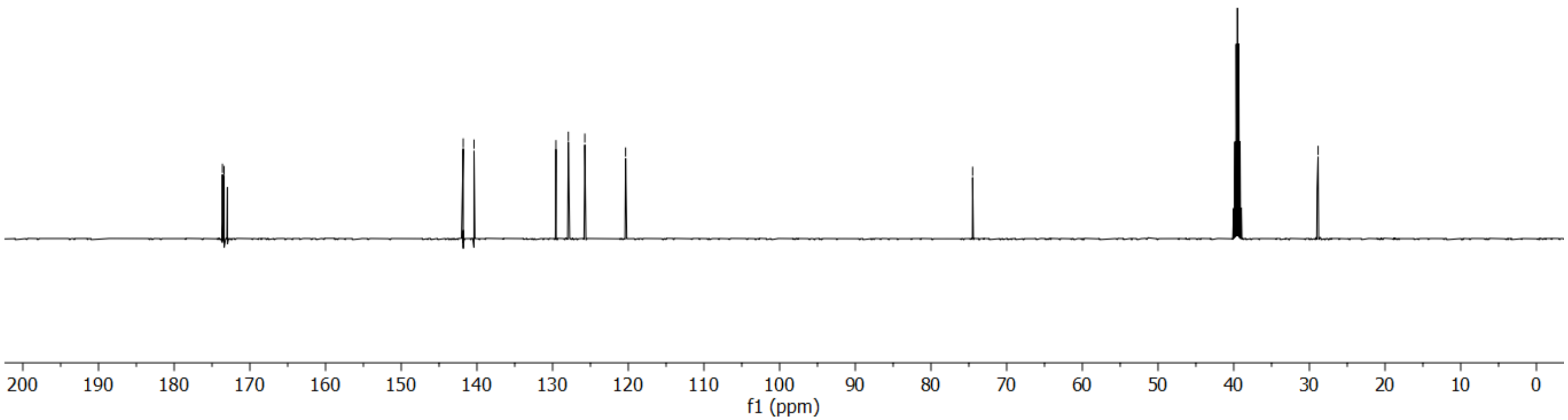

4-((2,3-dihydro-1H-inden-2-yl)oxy)-4-oxobutanoic acid (S6)

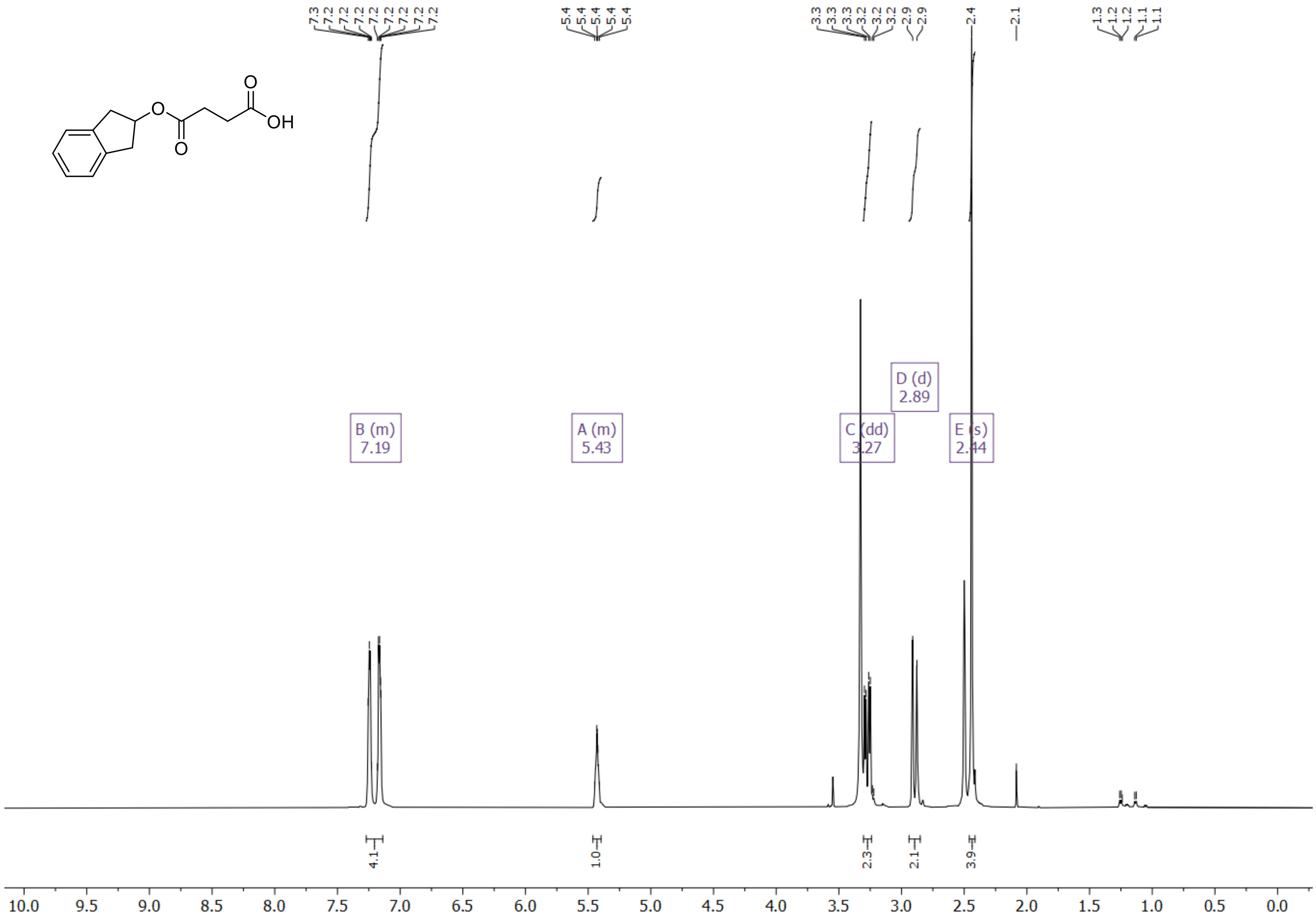

4-((2,3-dihydro-1H-inden-2-yl)oxy)-4-oxobutanoic acid (S6)

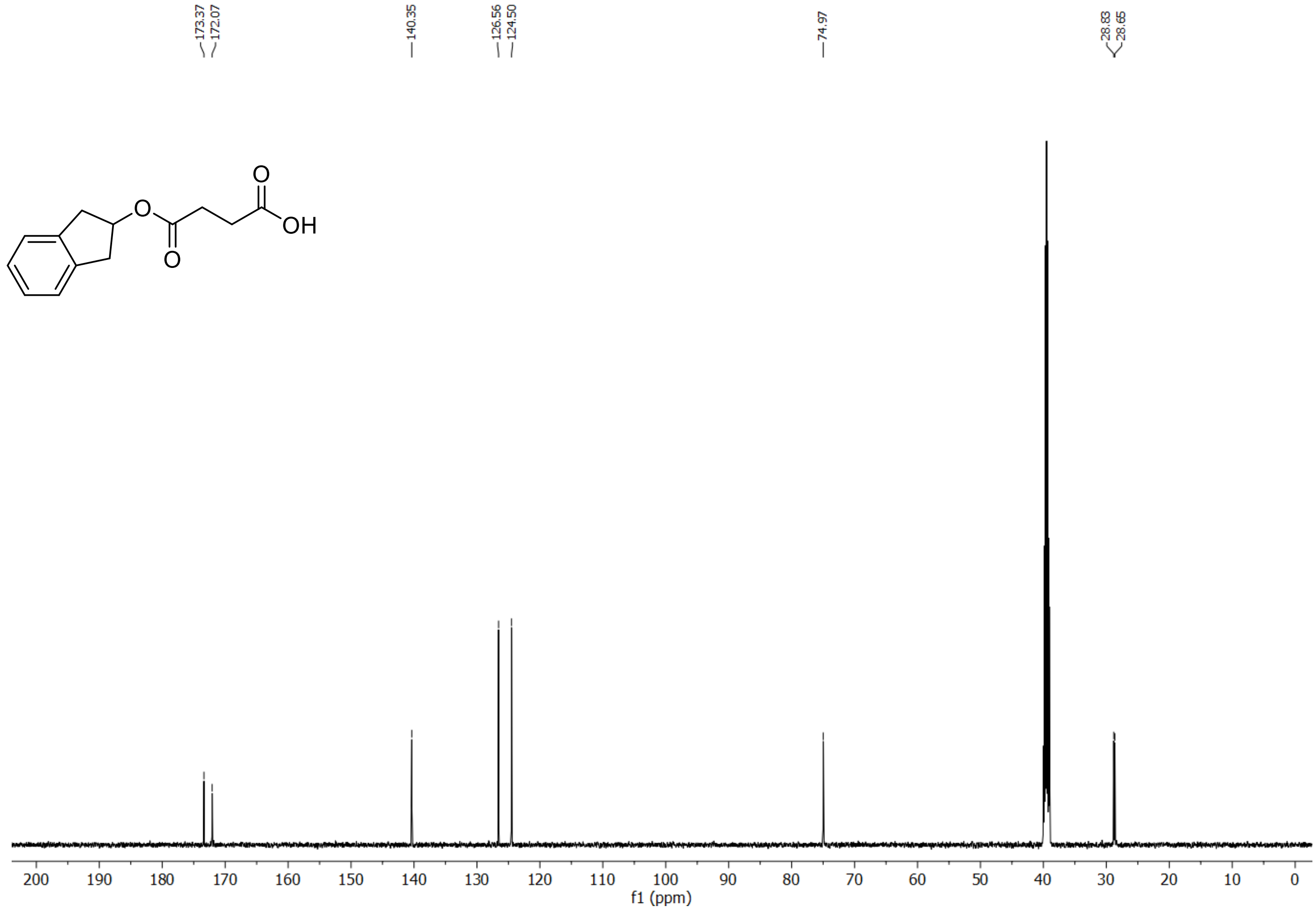

4-(((1S,2R,5S)-2-isopropyl-5-methylcyclohexyl)oxy)-4-oxobutanoic acid (S7)

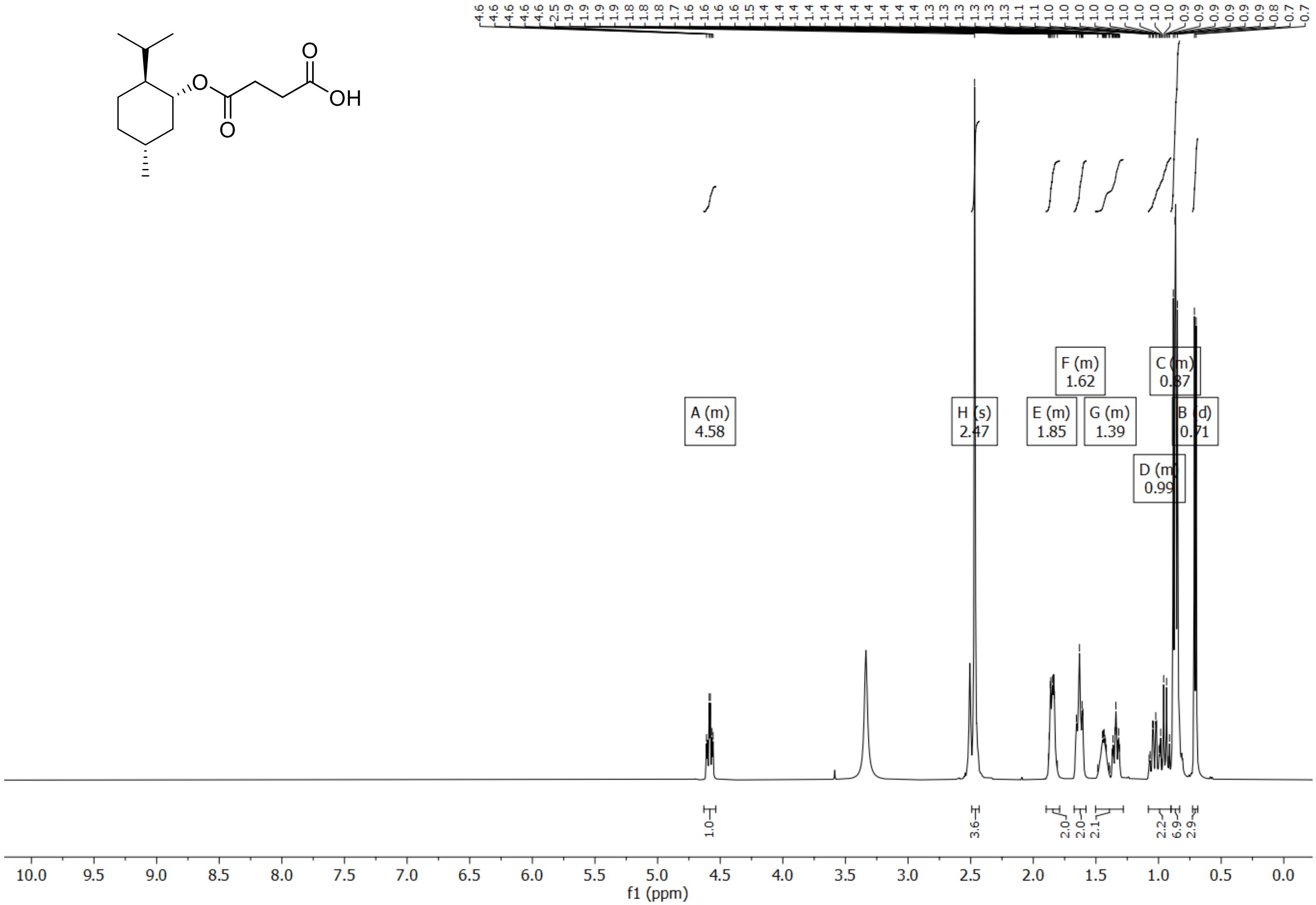

4-(((1S,2R,5S)-2-isopropyl-5-methylcyclohexyl)oxy)-4-oxobutanoic acid (S7)

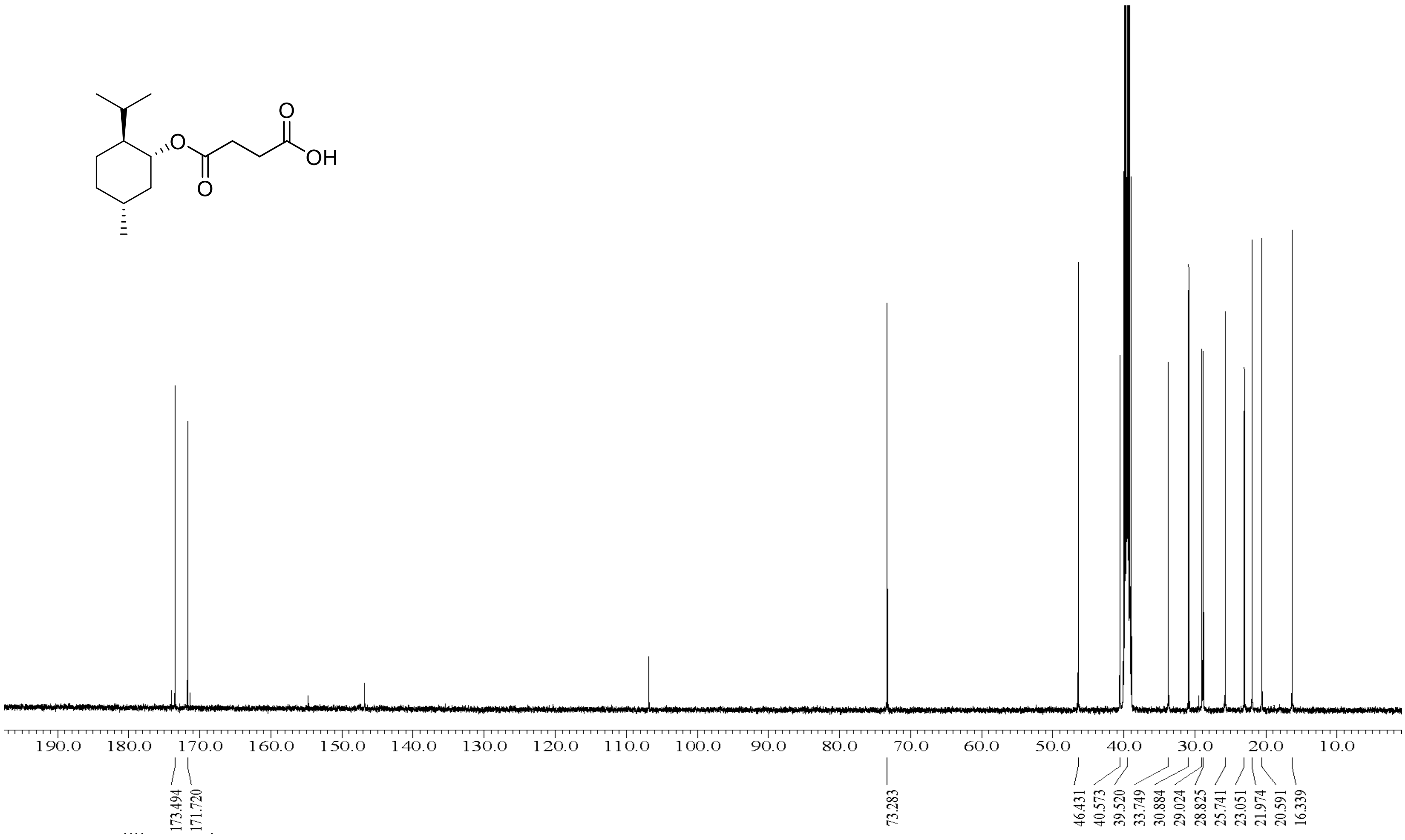

4-methoxy-4-oxobutanoic acid (S8)

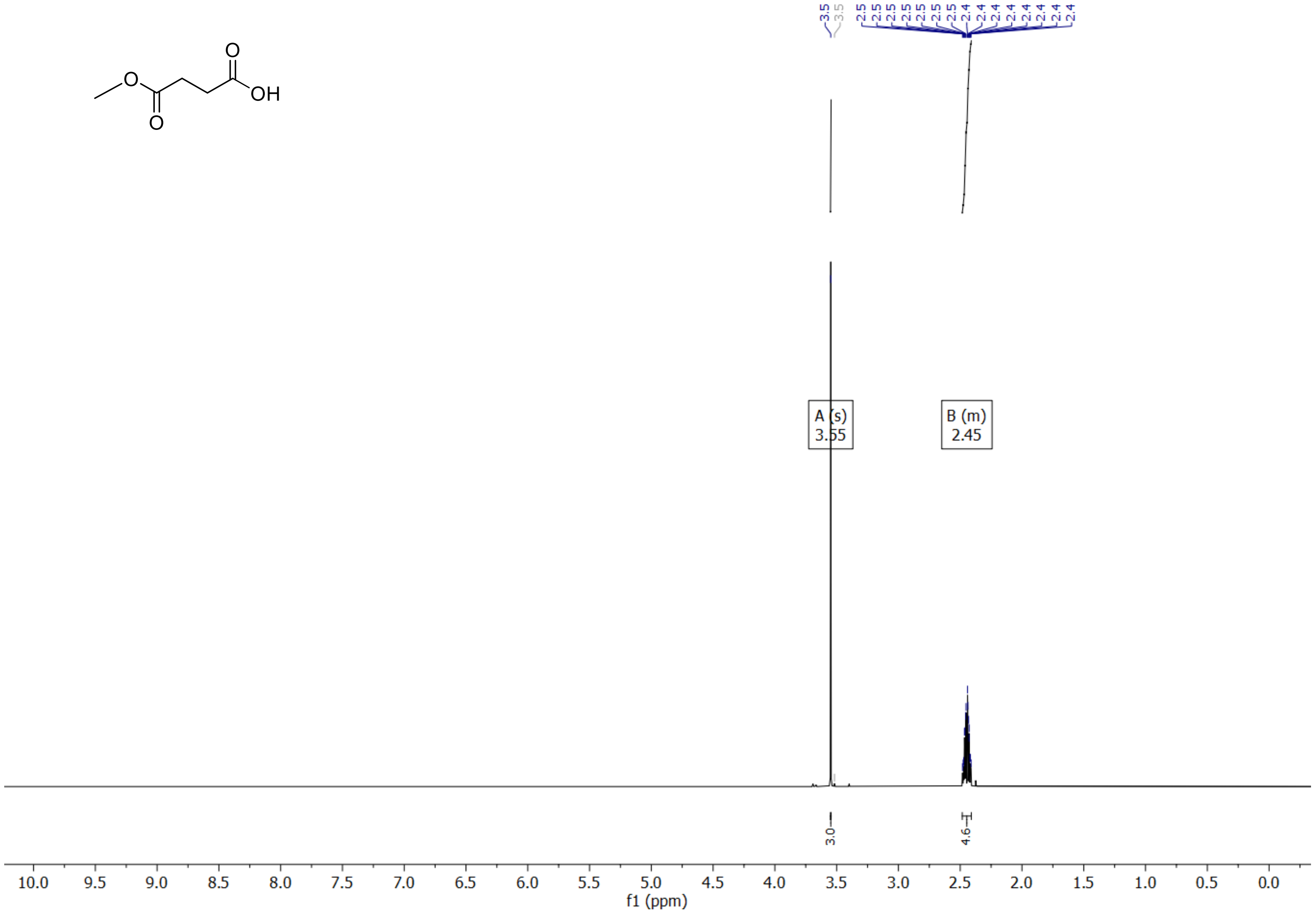

4-methoxy-4-oxobutanoic acid (S8)

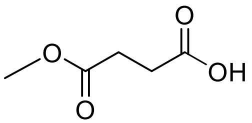

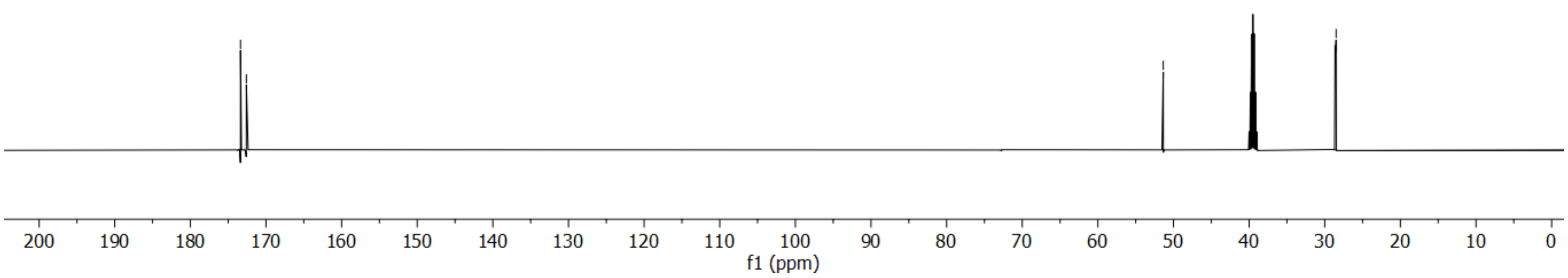

4-oxo-4-((1,2,3,4-tetrahydronaphthalen-1-yl)oxy)butanoic acid (S11)

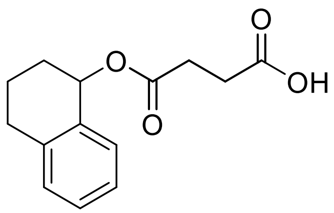

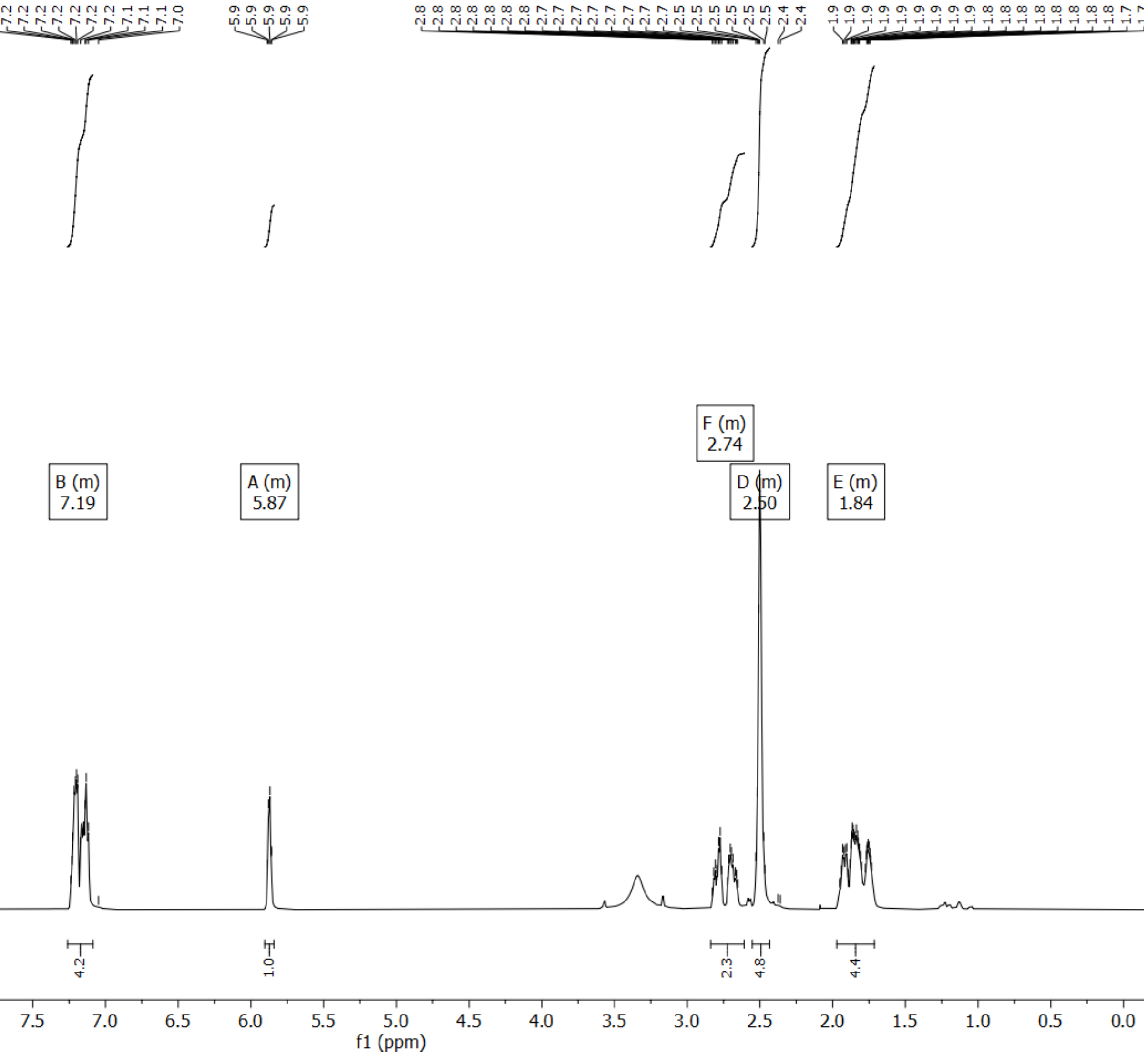

4-oxo-4-((1,2,3,4-tetrahydronaphthalen-1-yl)oxy)butanoic acid (S11)

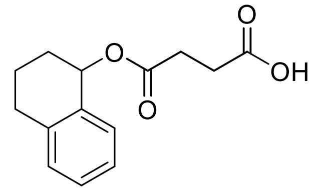

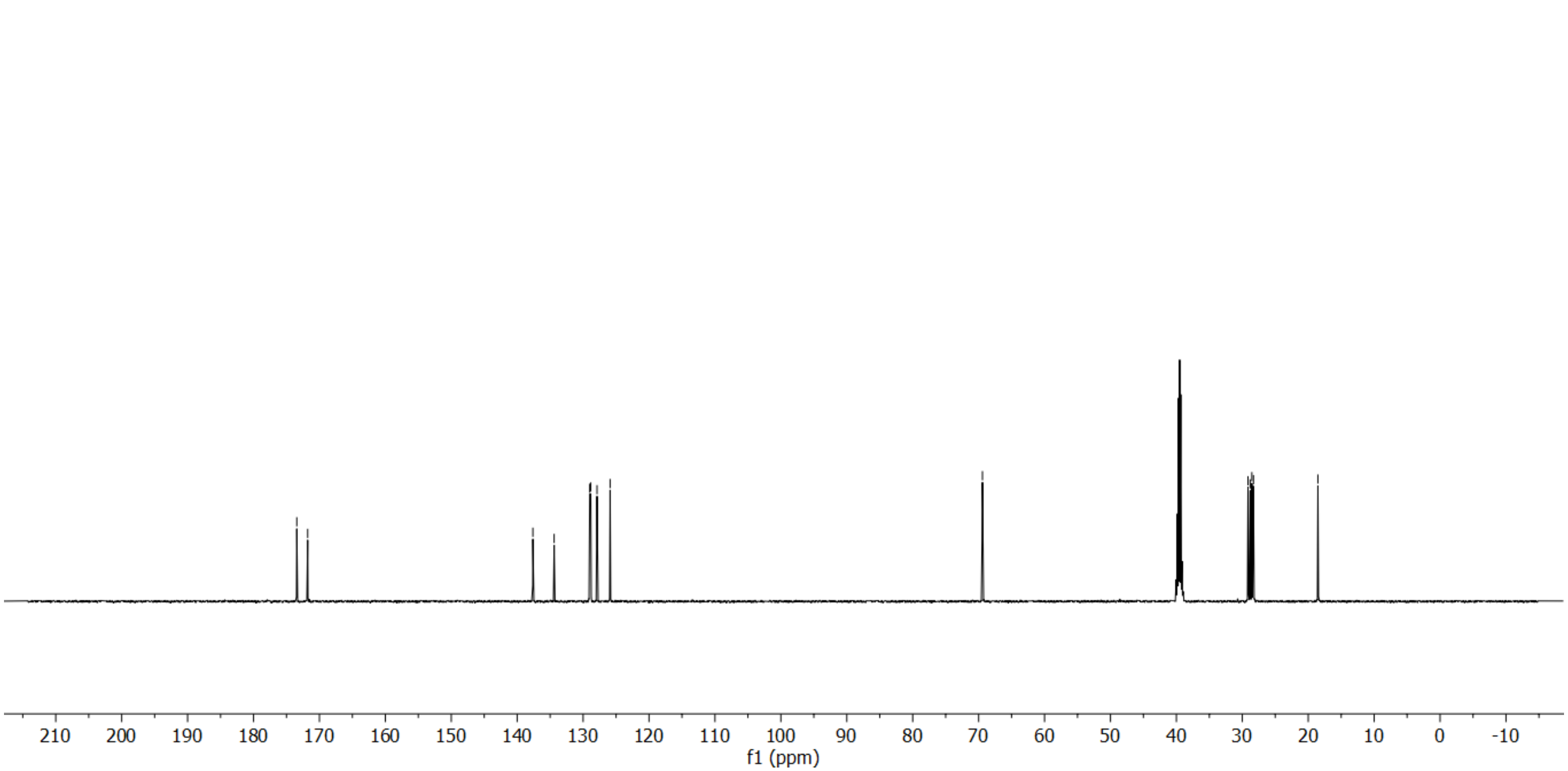

(1E,4Z,6E)-7-(4-(benzyloxy)-3-methoxyphenyl)-5-hydroxy-1-(4-hydroxy-3-methoxyphenyl)hepta-1,4,6-trien-3-one, (2)

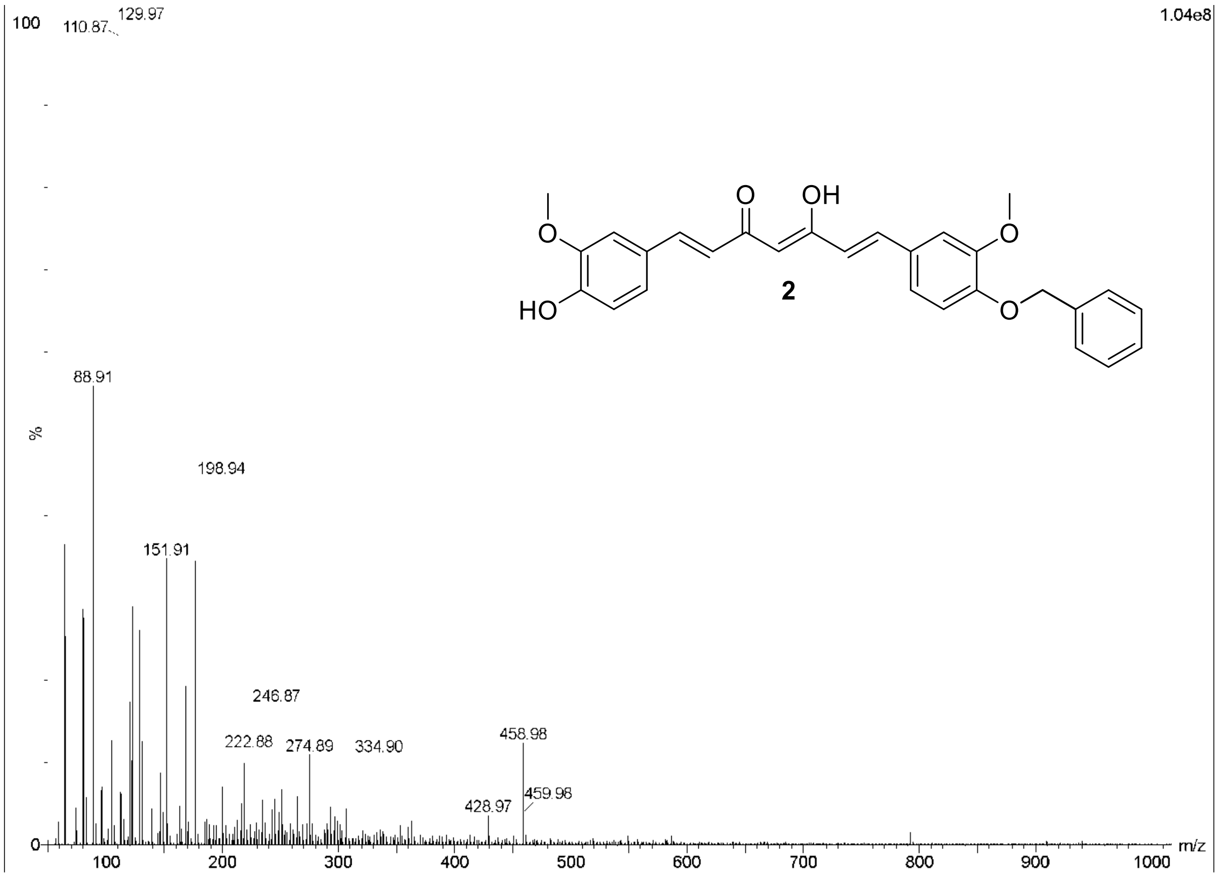

di((1r,3r,5r,7r)-adamantan-2-yl) *O,O′*-(((1*E*,3*Z*,6*E*)-3-hydroxy-5-oxohepta-1,3,6-triene-1,7-diyl)bis(2-methoxy-4,1-phenylene)) disuccinate (3).

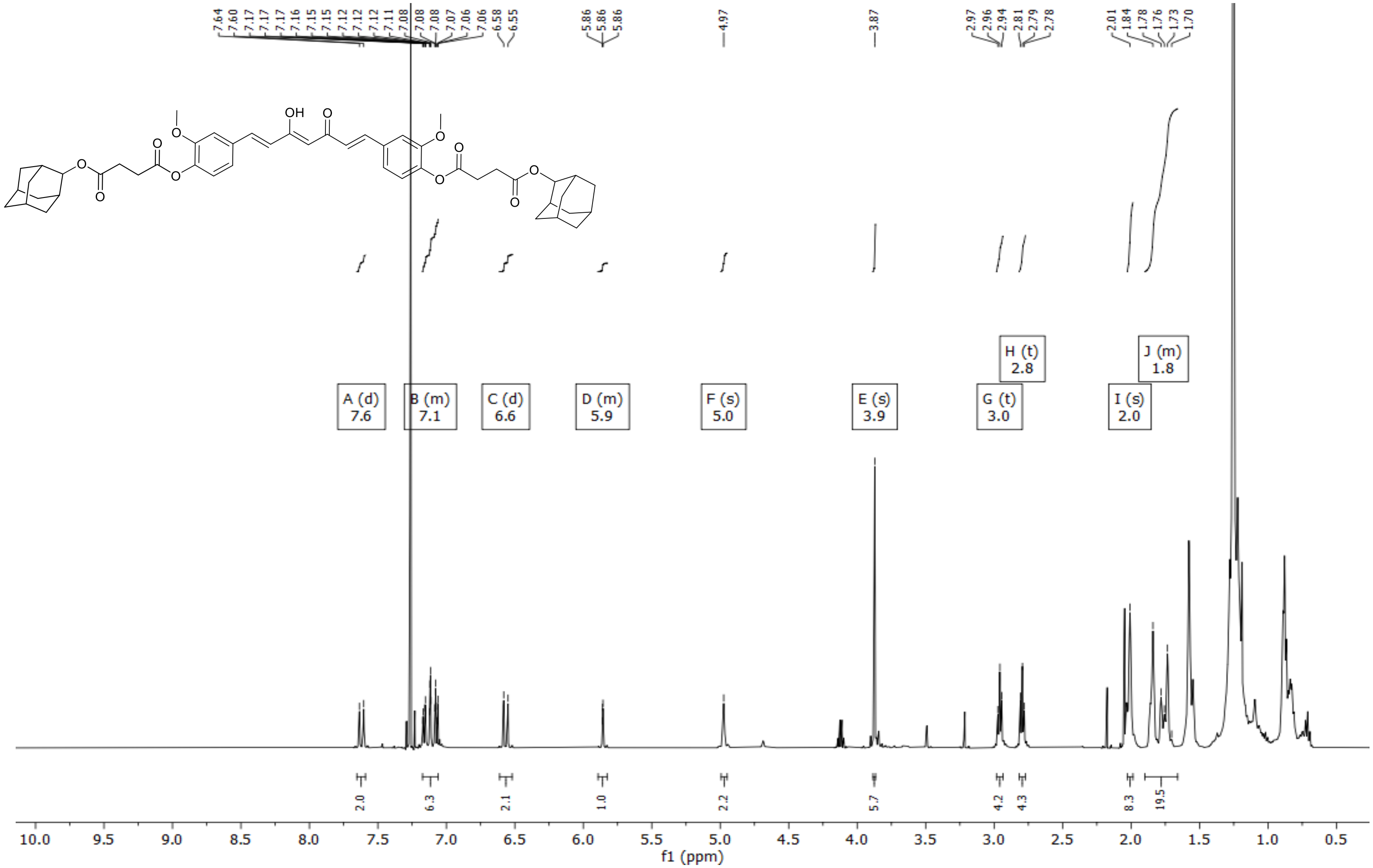

di((1r,3r,5r,7r)-adamantan-2-yl) *O,O′*-(((1*E*,3*Z*,6*E*)-3-hydroxy-5-oxohepta-1,3,6-triene-1,7-diyl)bis(2-methoxy-4,1-phenylene)) disuccinate (3).

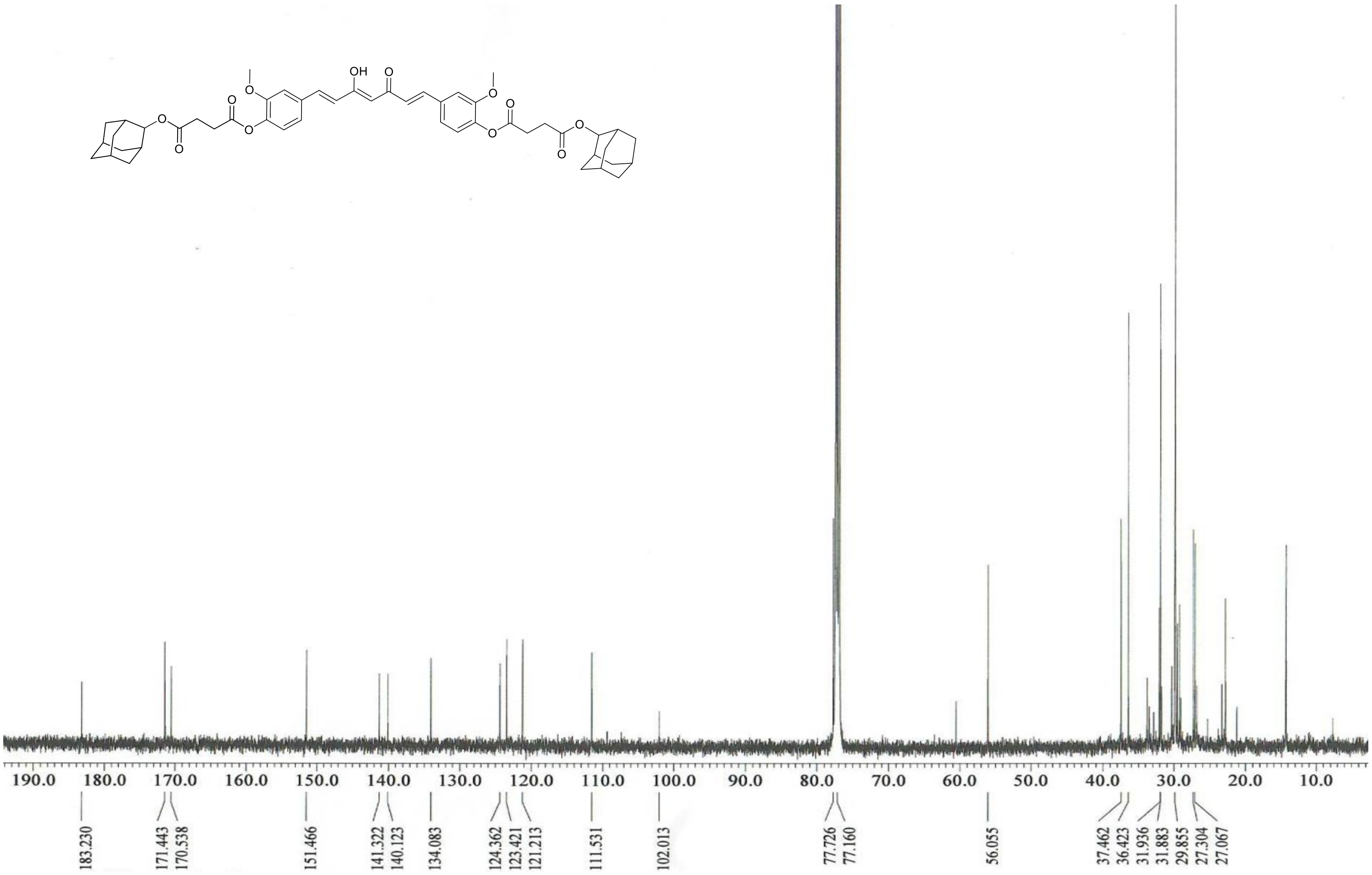

(1r,3r,5r,7r)-adamantan-2-yl(4-((1*E*,4*Z*,6*E*)-5-hydroxy-7-(4-hydroxy-3-methoxyphenyl)-3-oxohepta-1,4,6-trien-1-yl)-2-methoxyphenyl) succinate (4).

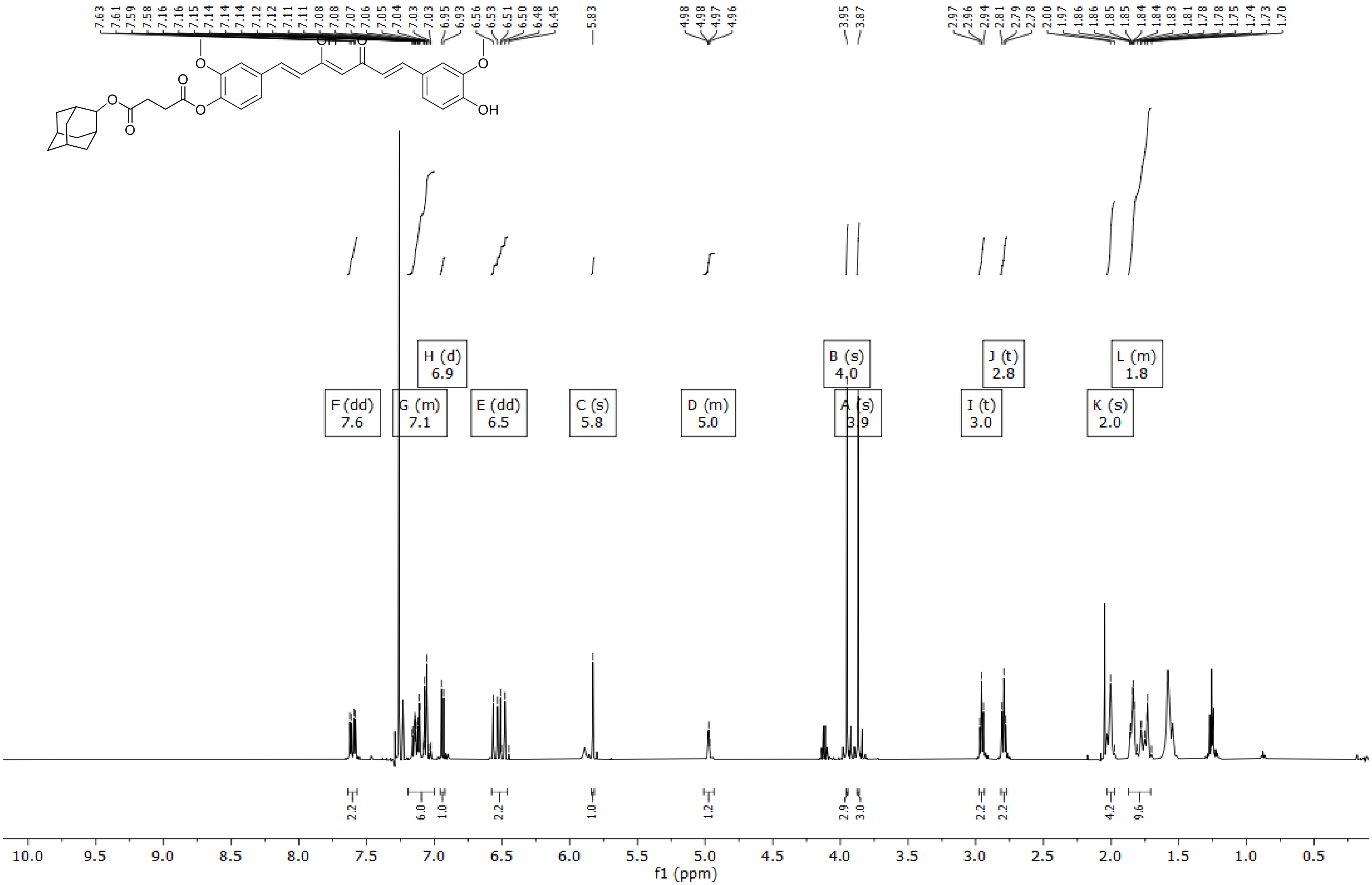

(1r,3r,5r,7r)-adamantan-2-yl (4-((1*E*,4*Z*,6*E*)-5-hydroxy-7-(4-hydroxy-3-methoxyphenyl)-3-oxohepta-1,4,6-trien-1-yl)-2-methoxyphenyl) succinate (4).

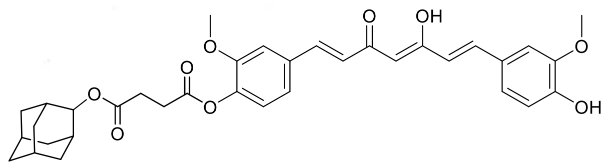

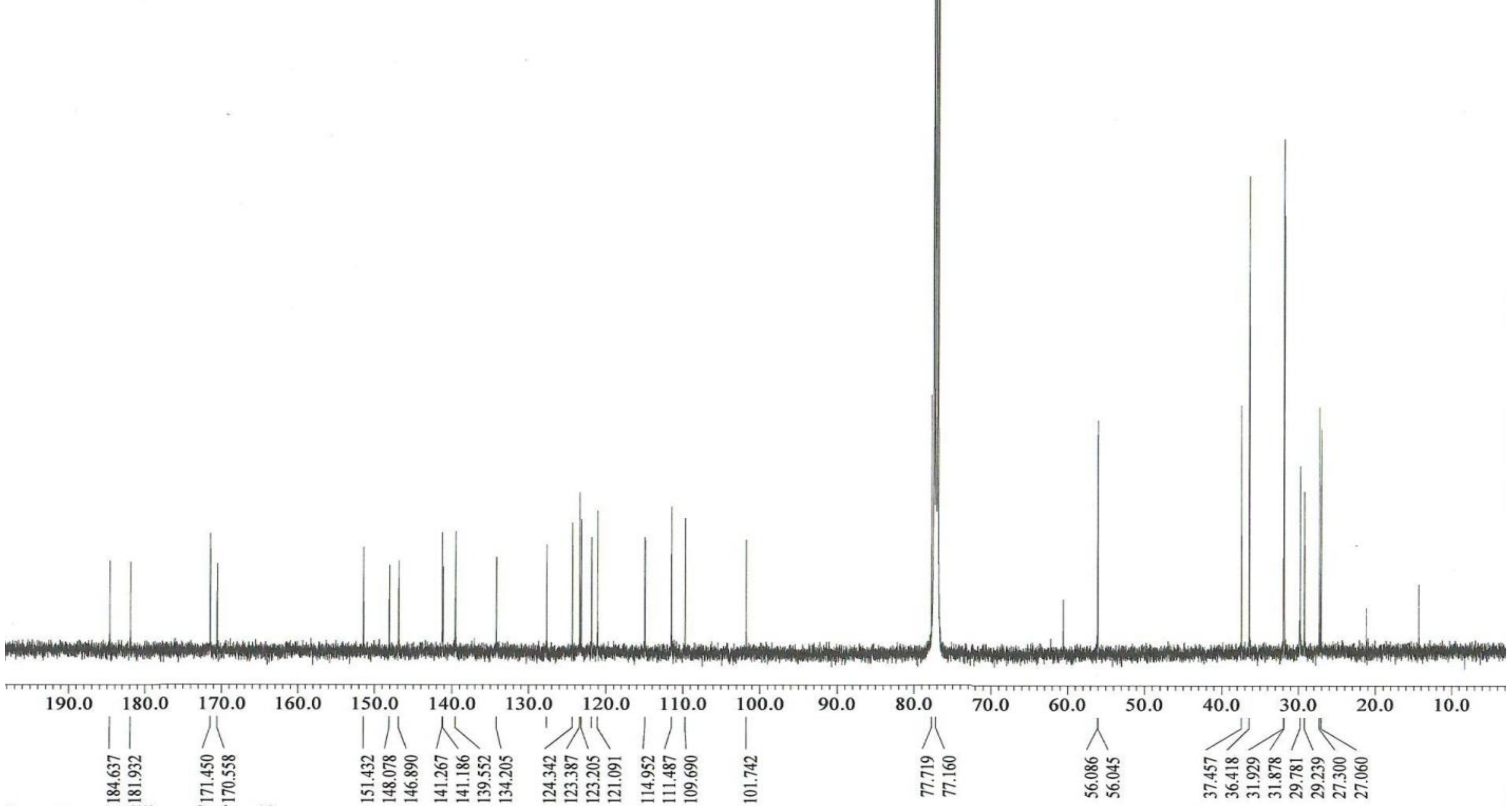

(4-((1*E*,4*Z*,6*E*)-5-hydroxy-7-(4-hydroxy-3-methoxyphenyl)-3-oxohepta-1,4,6-trien-1-yl)-2-methoxyphenyl) succinate (5)

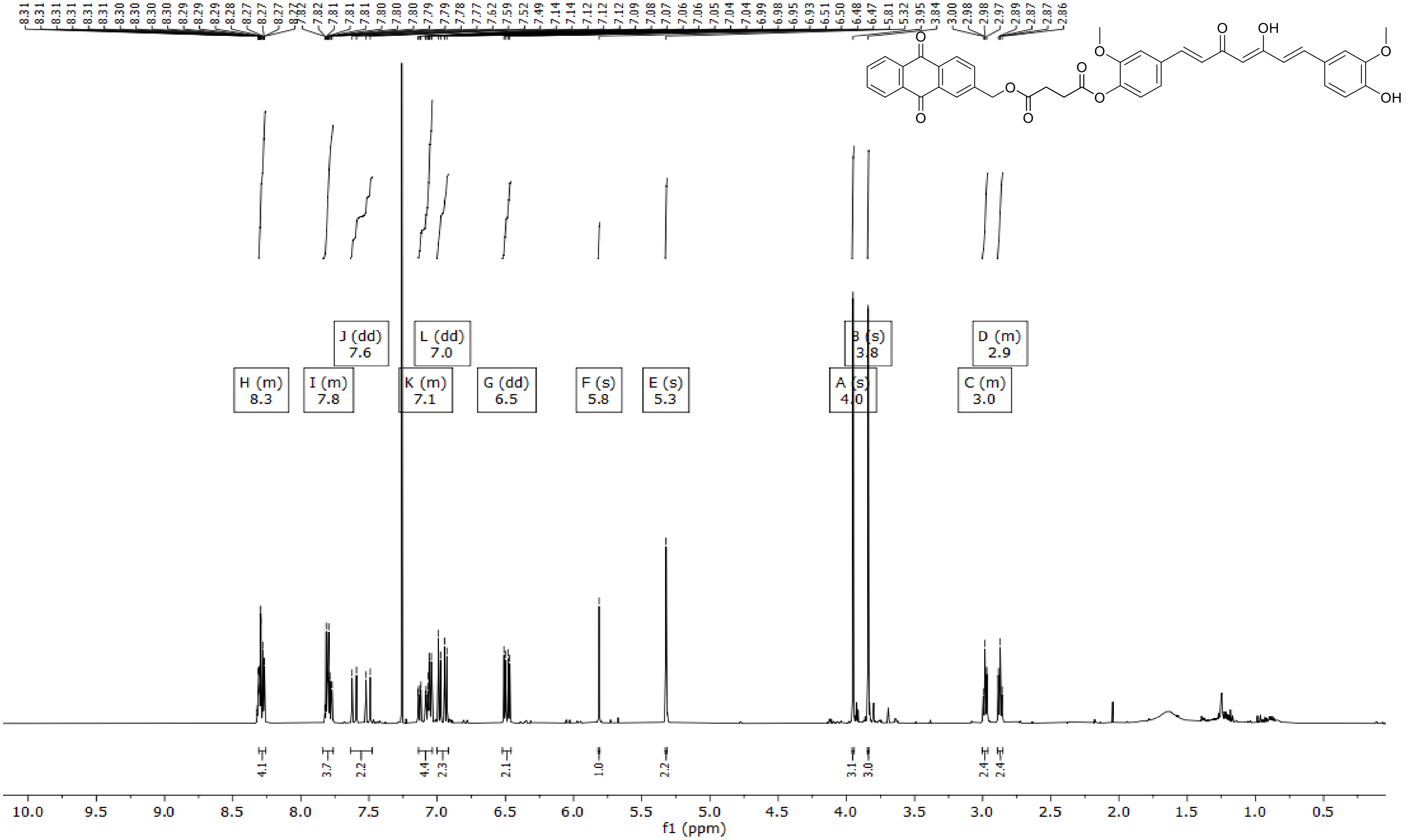

(9,10-dioxo-9,10-dihydroanthracen-2-yl)methyl (4-((1*E*,4*Z*,6*E*)-5-hydroxy-7-(4-hydroxy-3-methoxyphenyl)-3-oxohepta-1,4,6-trien-1-yl)-2-methoxyphenyl) succinate (5).

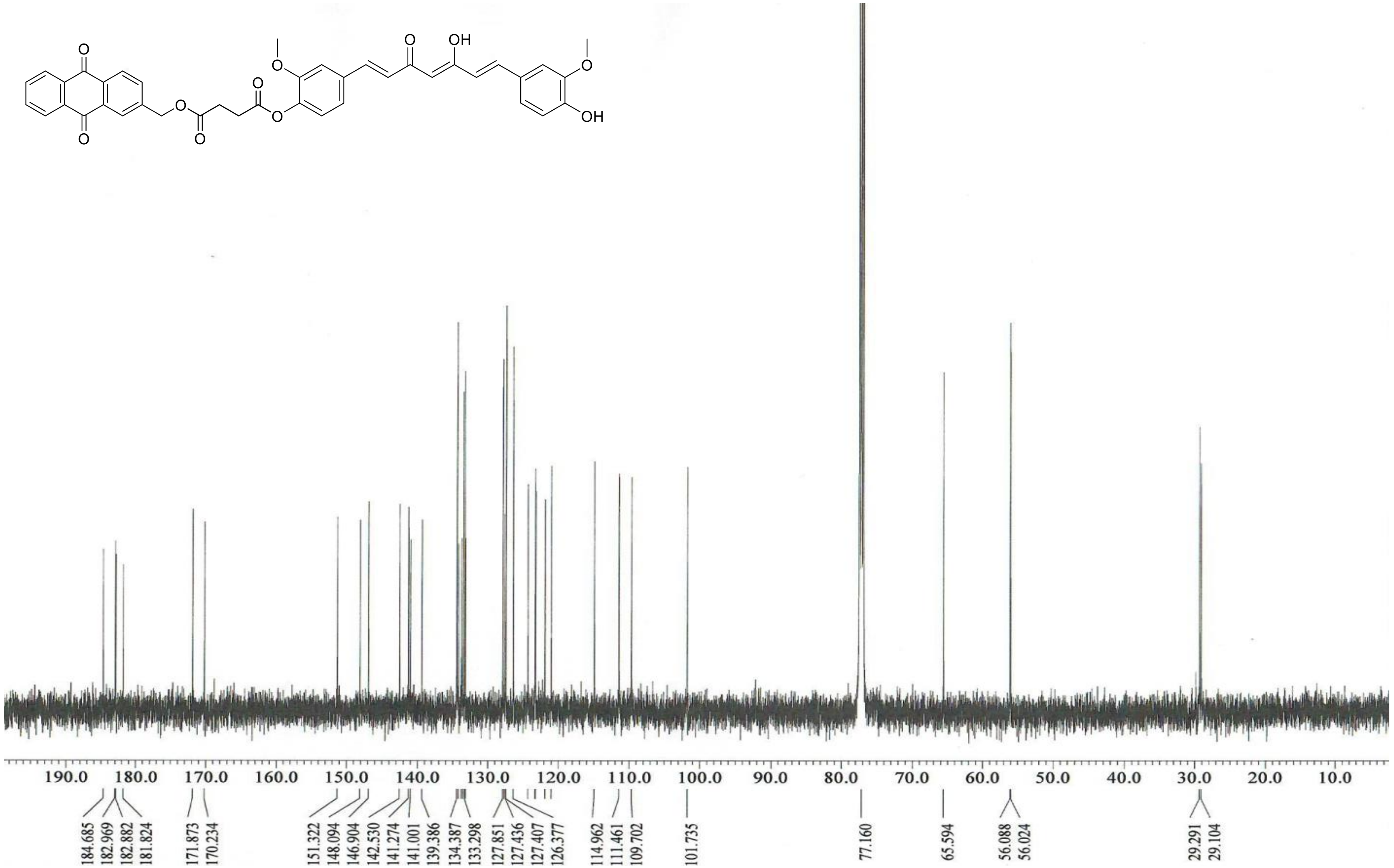

(4-((1*E*,4*Z*,6*E*)-5-hydroxy-7-(4-hydroxy-3-methoxyphenyl)-3-oxohepta-1,4,6-trien-1-yl)-2-methoxyphenyl) succinate (6).

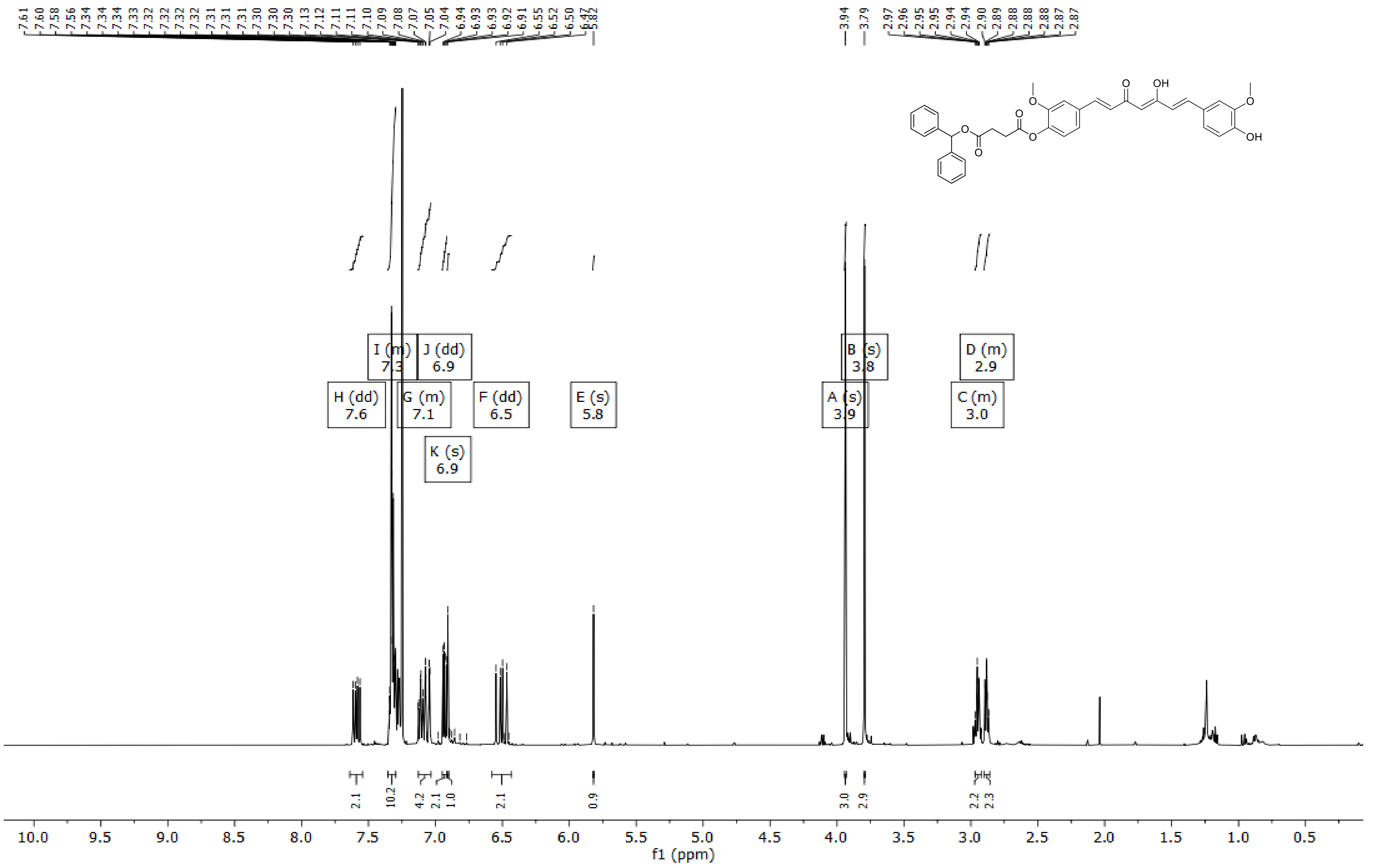

(4-((1*E*,4*Z*,6*E*)-5-hydroxy-7-(4-hydroxy-3-methoxyphenyl)-3-oxohepta-1,4,6-trien-1-yl)-2-methoxyphenyl) succinate (6).

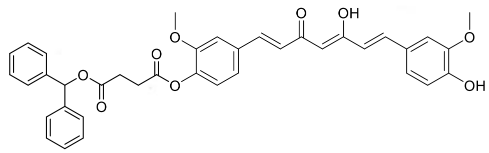

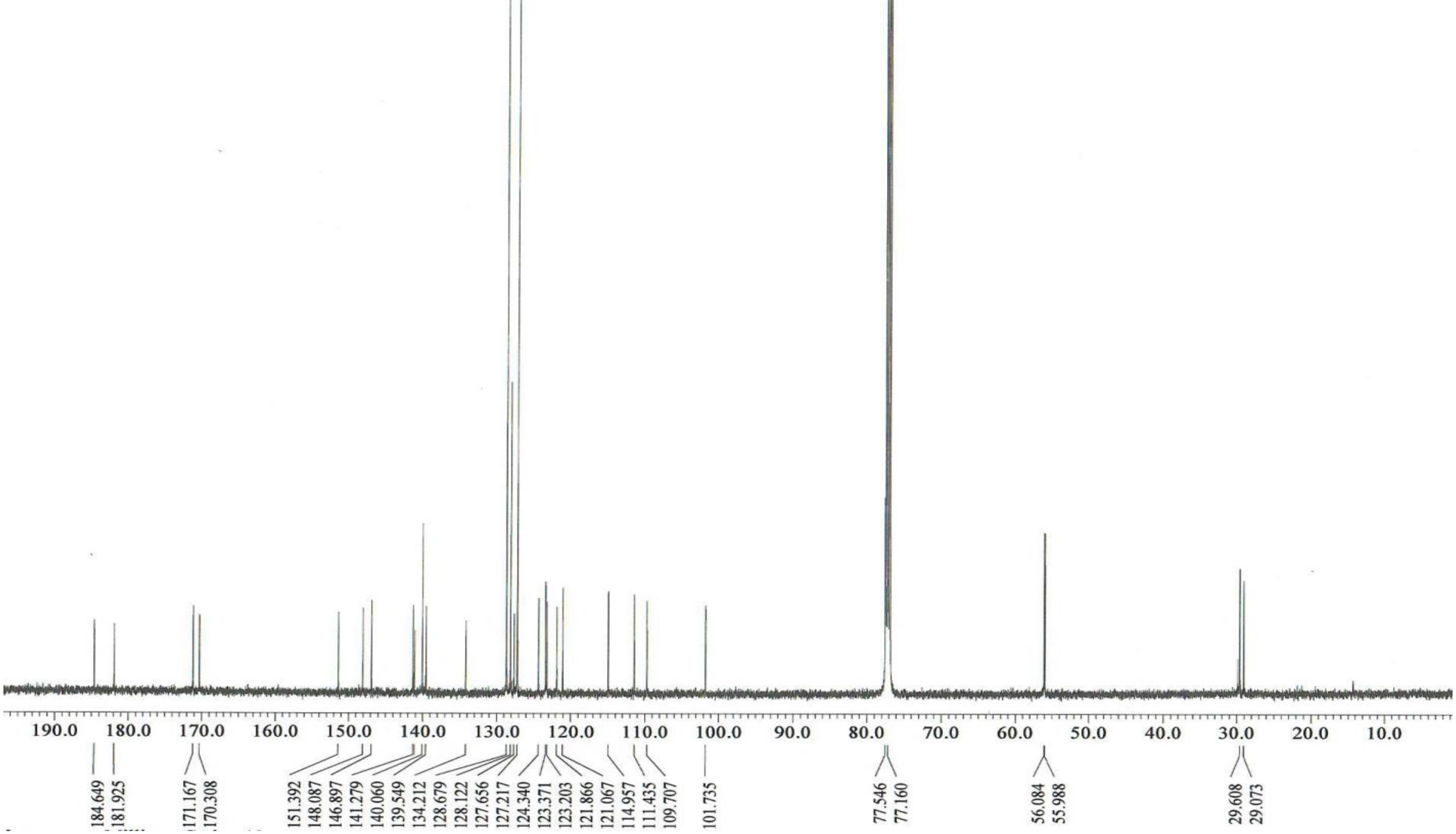

Bis(cyclohexyl(phenyl)methyl) *O,O′*-(((1*E*,3*Z*,6*E*)-3-hydroxy-5-oxohepta-1,3,6-triene-1,7-diyl)bis(2-methoxy-4,1-phenylene)) disuccinate (7).

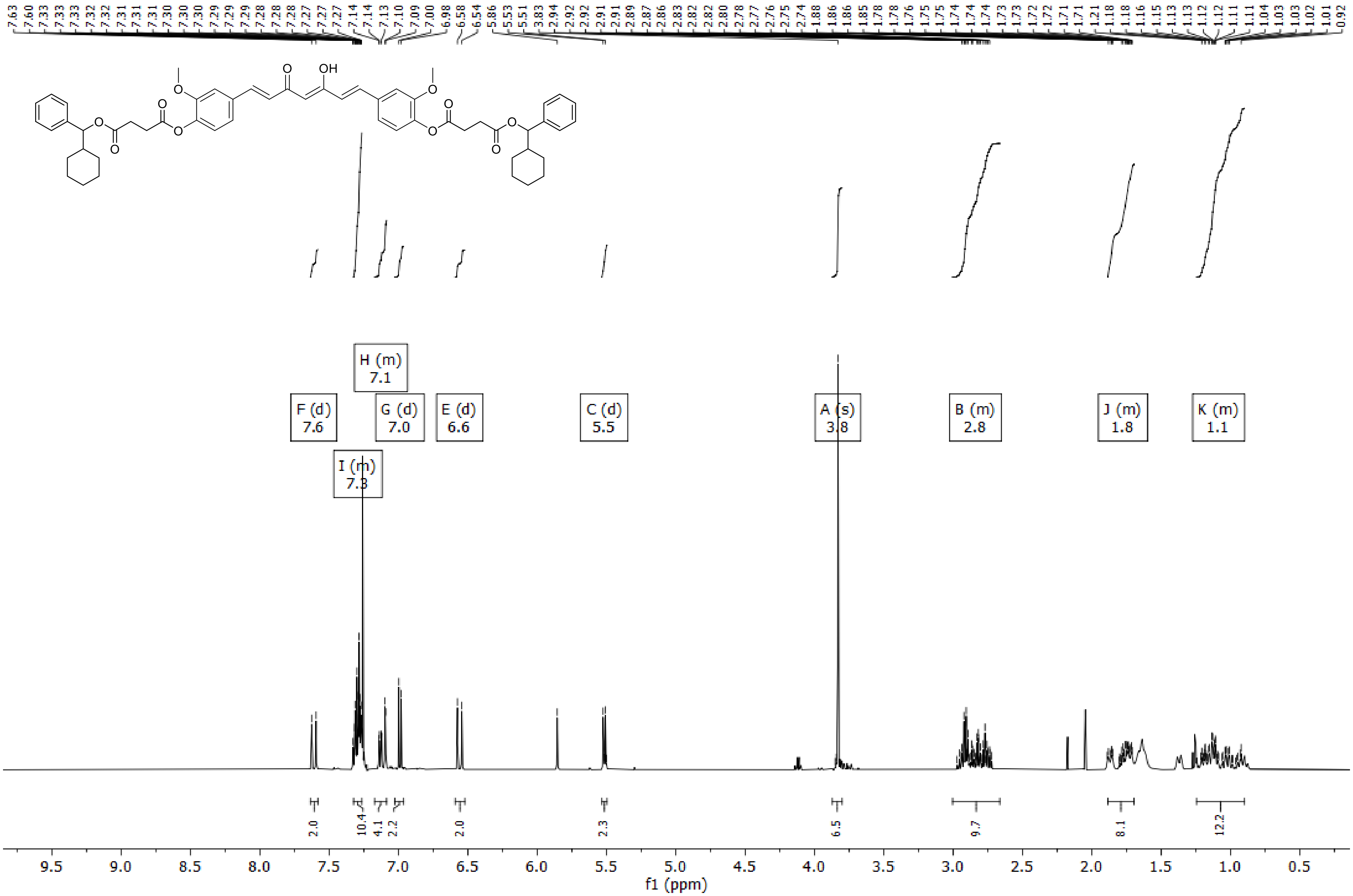

Bis(cyclohexyl(phenyl)methyl) *O,O'*-(((1*E*,3*Z*,6*E*)-3-hydroxy-5-oxohepta-1,3,6-triene-1,7-diyl)bis(2-methoxy-4,1-phenylene)) disuccinate (7).

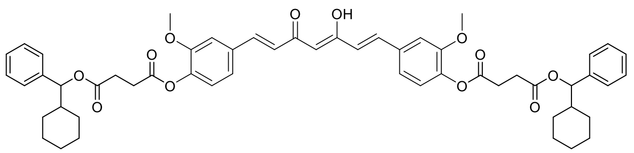

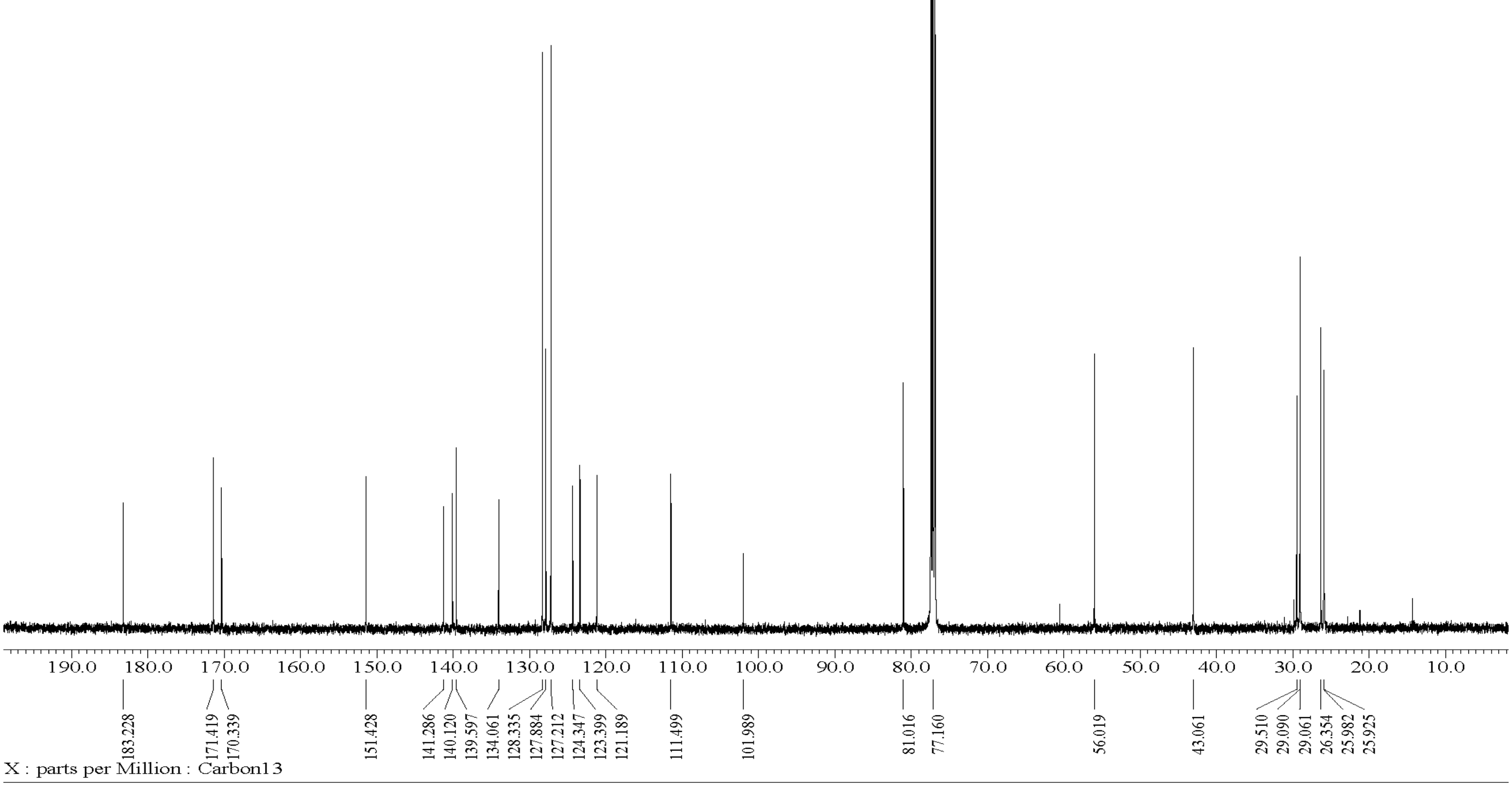

cyclohexyl(phenyl)methyl (4-((1*E*,4*Z*,6*E*)-5-hydroxy-7-(4-hydroxy-3-methoxyphenyl)-3-oxohepta-1,4,6-trien-1-yl)-2-methoxyphenyl) succinate (8).

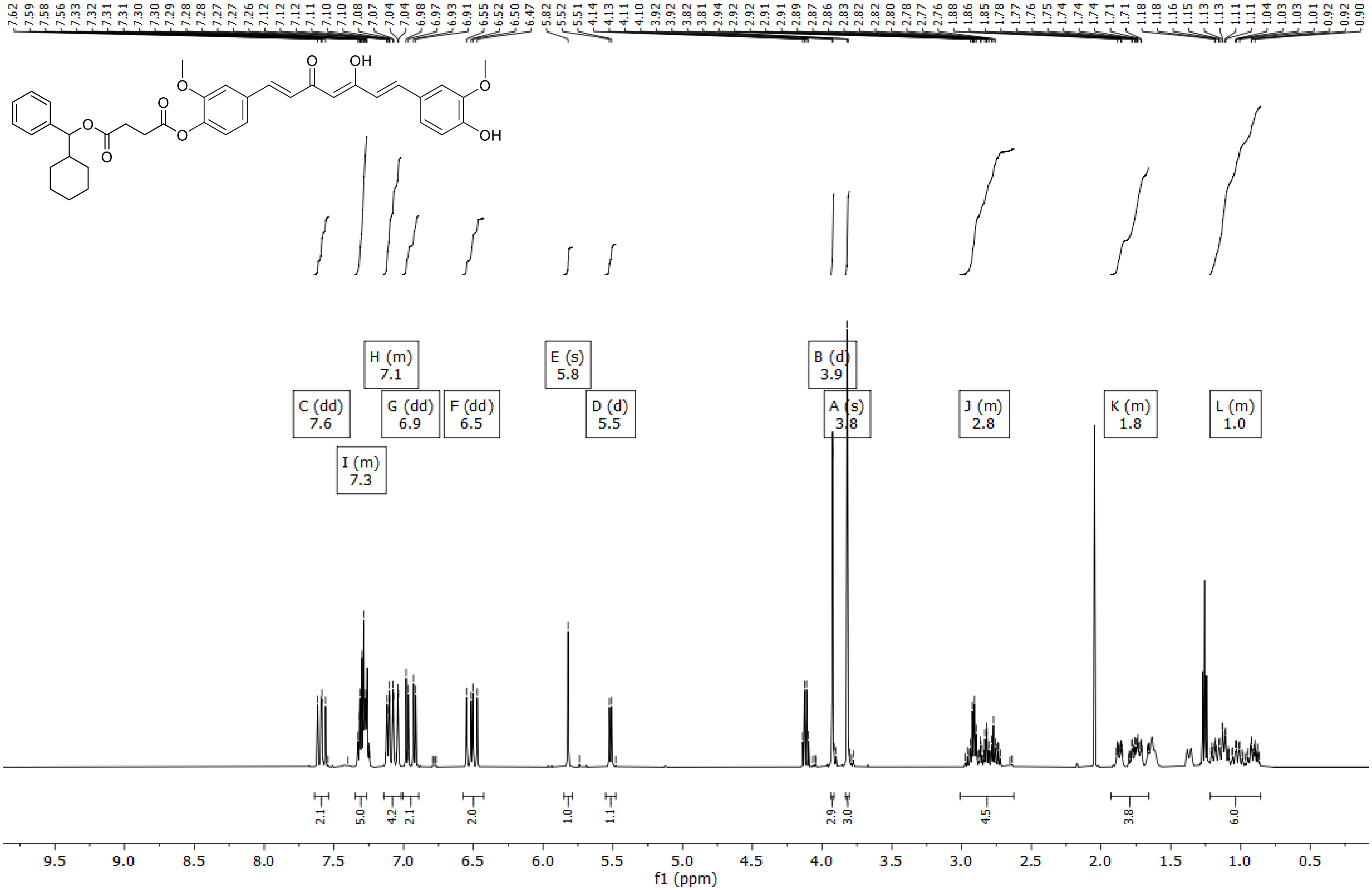

cyclohexyl(phenyl)methyl (4-((1*E*,4*Z*,6*E*)-5-hydroxy-7-(4-hydroxy-3-methoxyphenyl)-3-oxohepta-1,4,6-trien-1-yl)-2-methoxyphenyl) succinate (8).

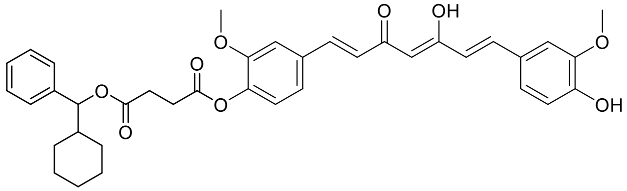

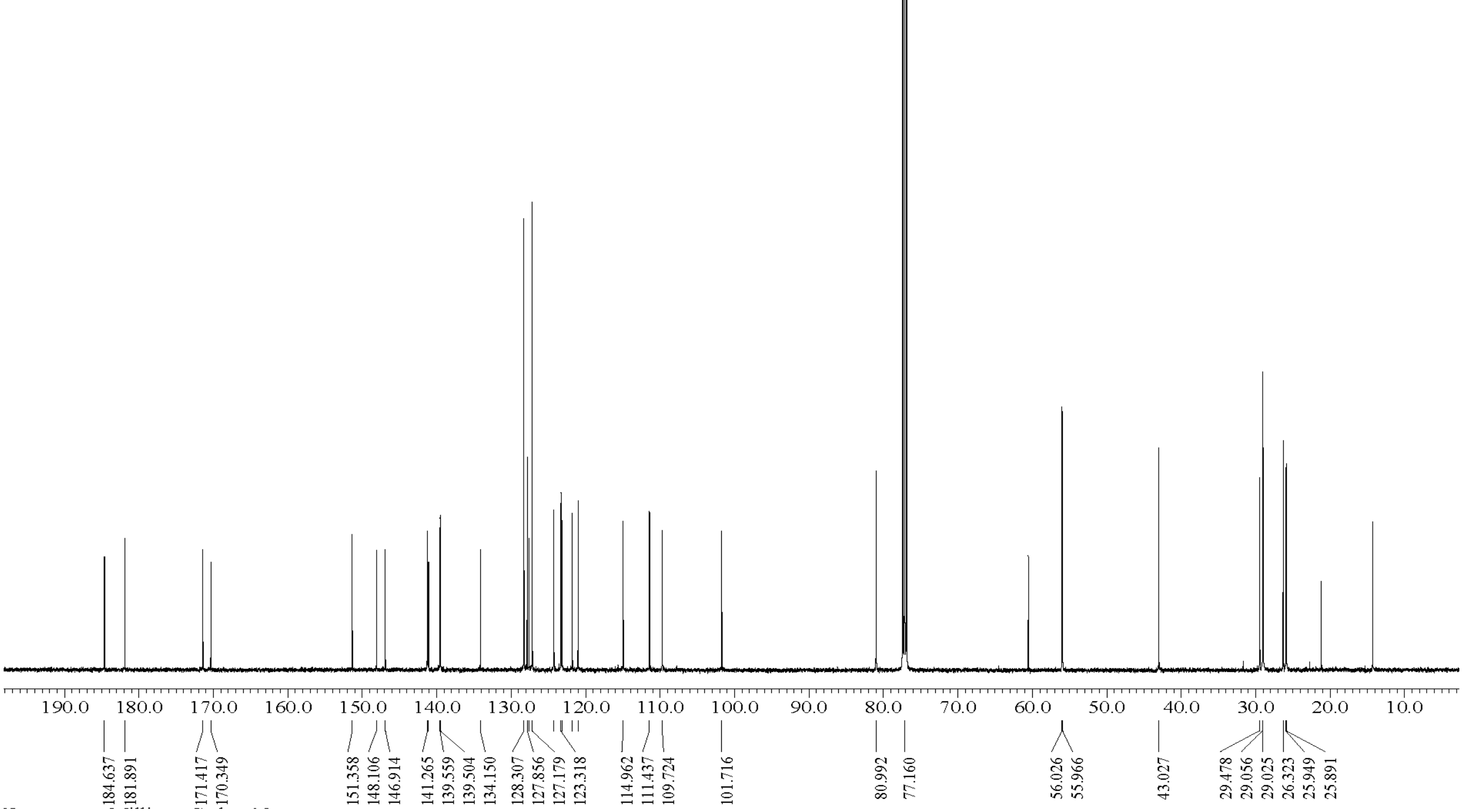

Di(9*H*-fluoren-9-yl) *O*,*O′*-(((1*E*,3*Z*,6*E*)-3-hydroxy-5-oxohepta-1,3,6-triene-1,7-diyl)bis(2-methoxy-4,1-phenylene)) disuccinate

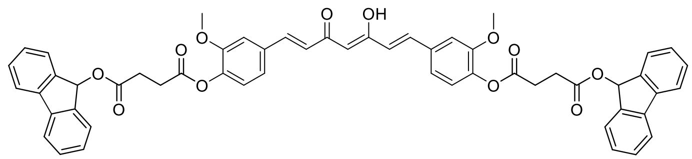

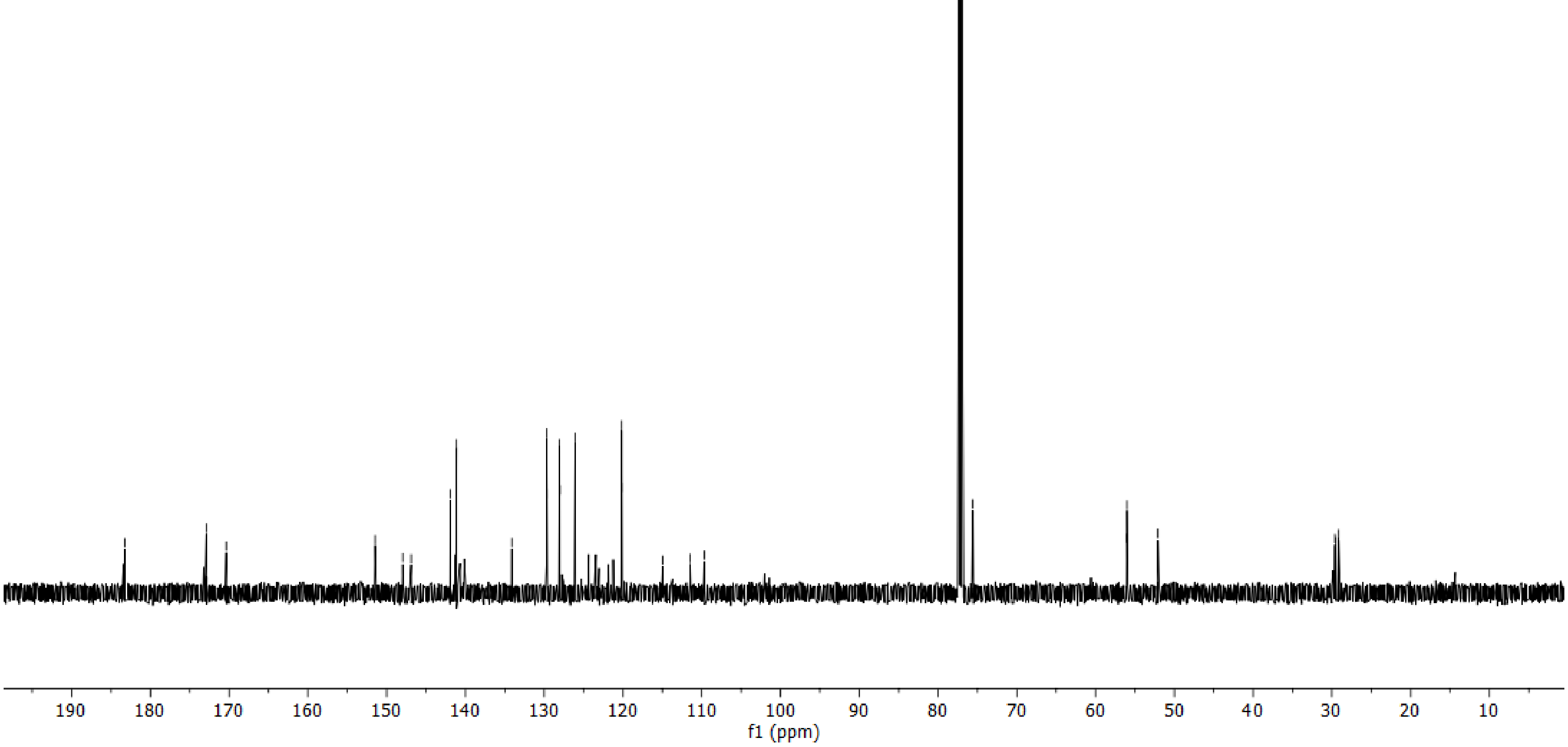

(9).

9*H*-fluoren-9-yl (4-((1*E*,4*Z*,6*E*)-5-hydroxy-7-(4-hydroxy-3-methoxyphenyl)-3-oxohepta-1,4,6-trien-1-yl)-2-methoxyphenyl) succinate (10).

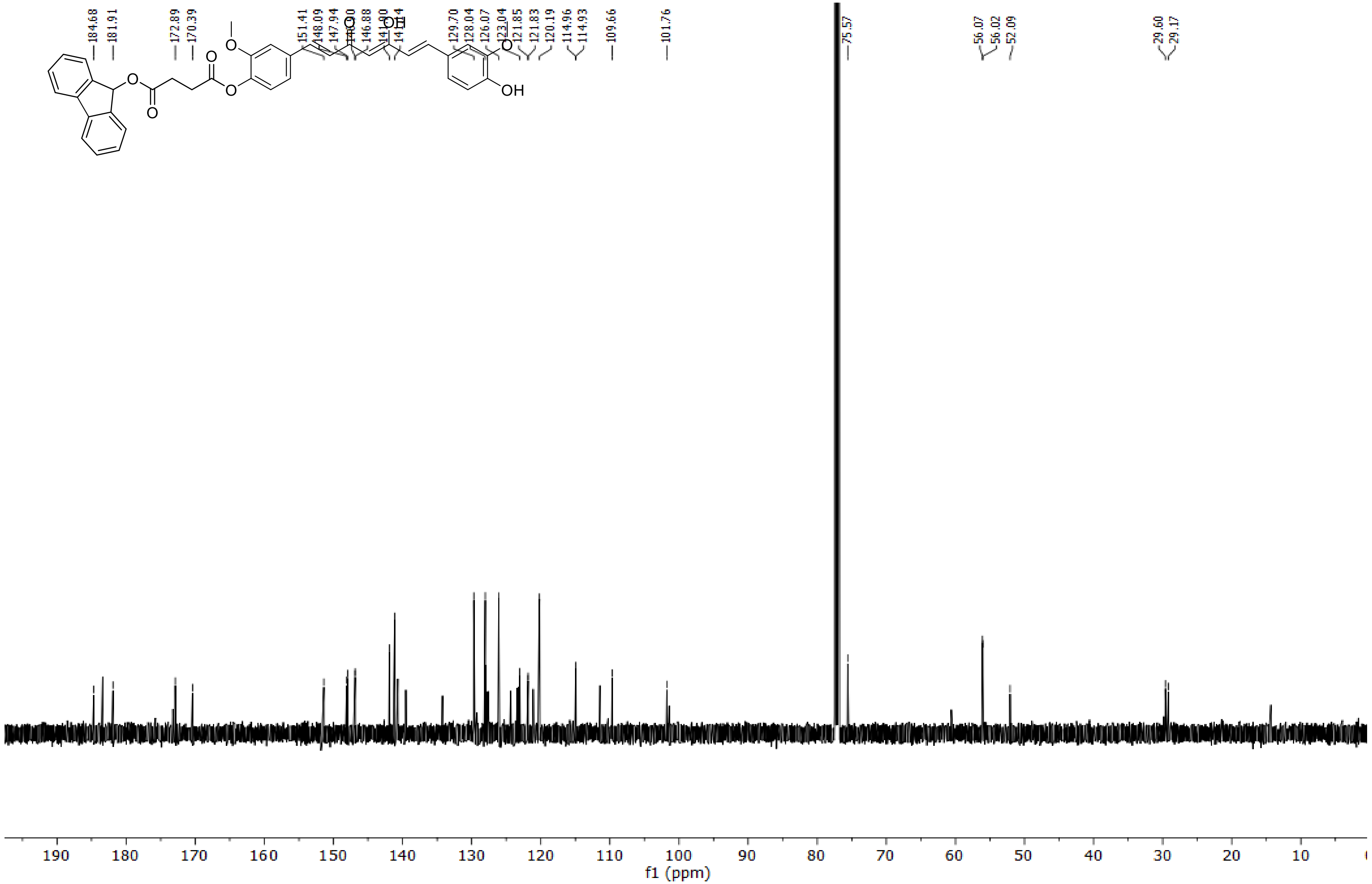

bis(2,3-dihydro-1H-inden-2-yl) *O,O′*-(((1*E*,3*Z*,6*E*)-3-hydroxy-5-oxohepta-1,3,6-triene-1,7-diyl)bis(2-methoxy-4,1-phenylene)) disuccinate (11).

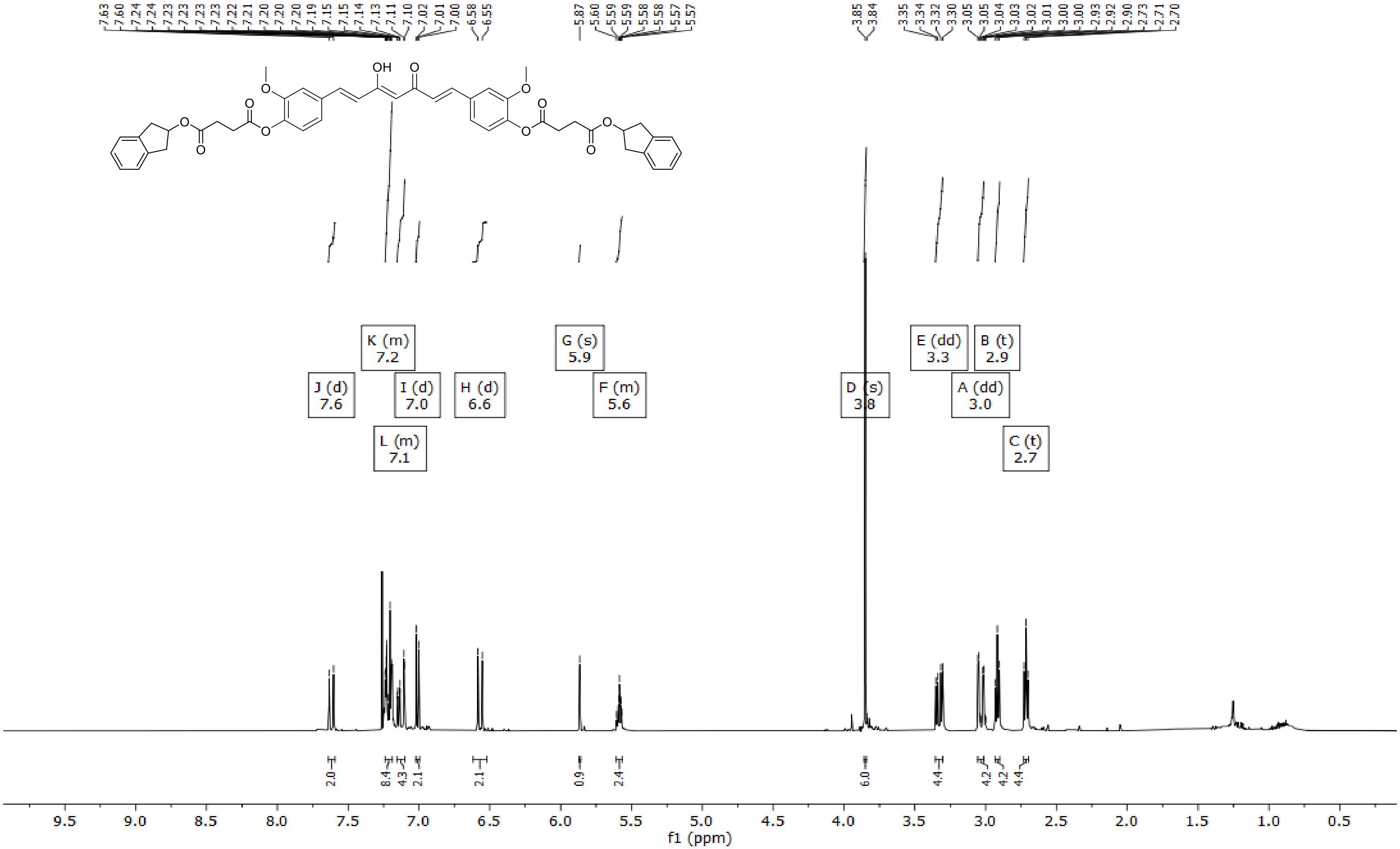

^{1}H and ^{13}C NMR spectra of bis(2,3-dihydro-1H-inden-2-yl) *O,O′*-(((1*E*,3*Z*,6*E*)-3-hydroxy-5-oxohepta-1,3,6-triene-1,7-diyl)bis(2-methoxy-4,1-phenylene)) disuccinate (11)

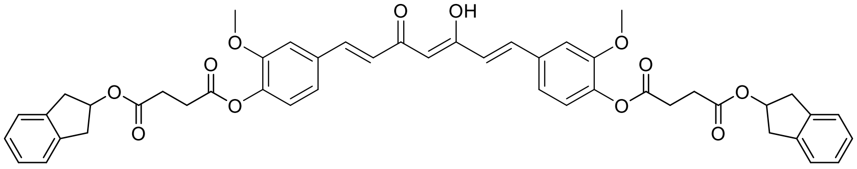

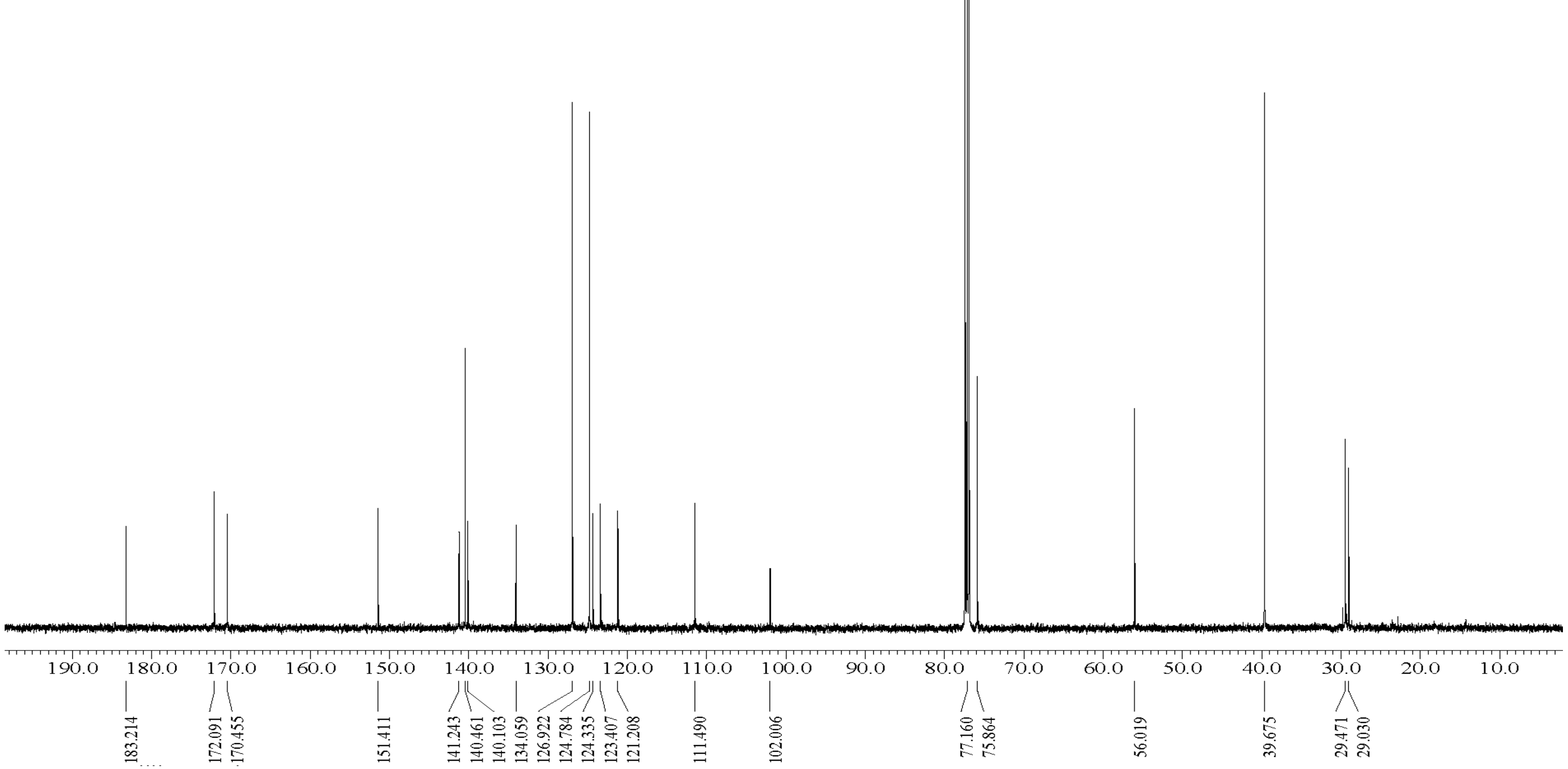

O,O′-(((1E,3Z,6E)-3-hydroxy-5-oxohepta-1,3,6-triene-1,7-diyl)bis(2-methoxy-4,1-phenylene)) bis((1R,2S,5R)-2-isopropyl-5-methylcyclohexyl) disuccinate (12).

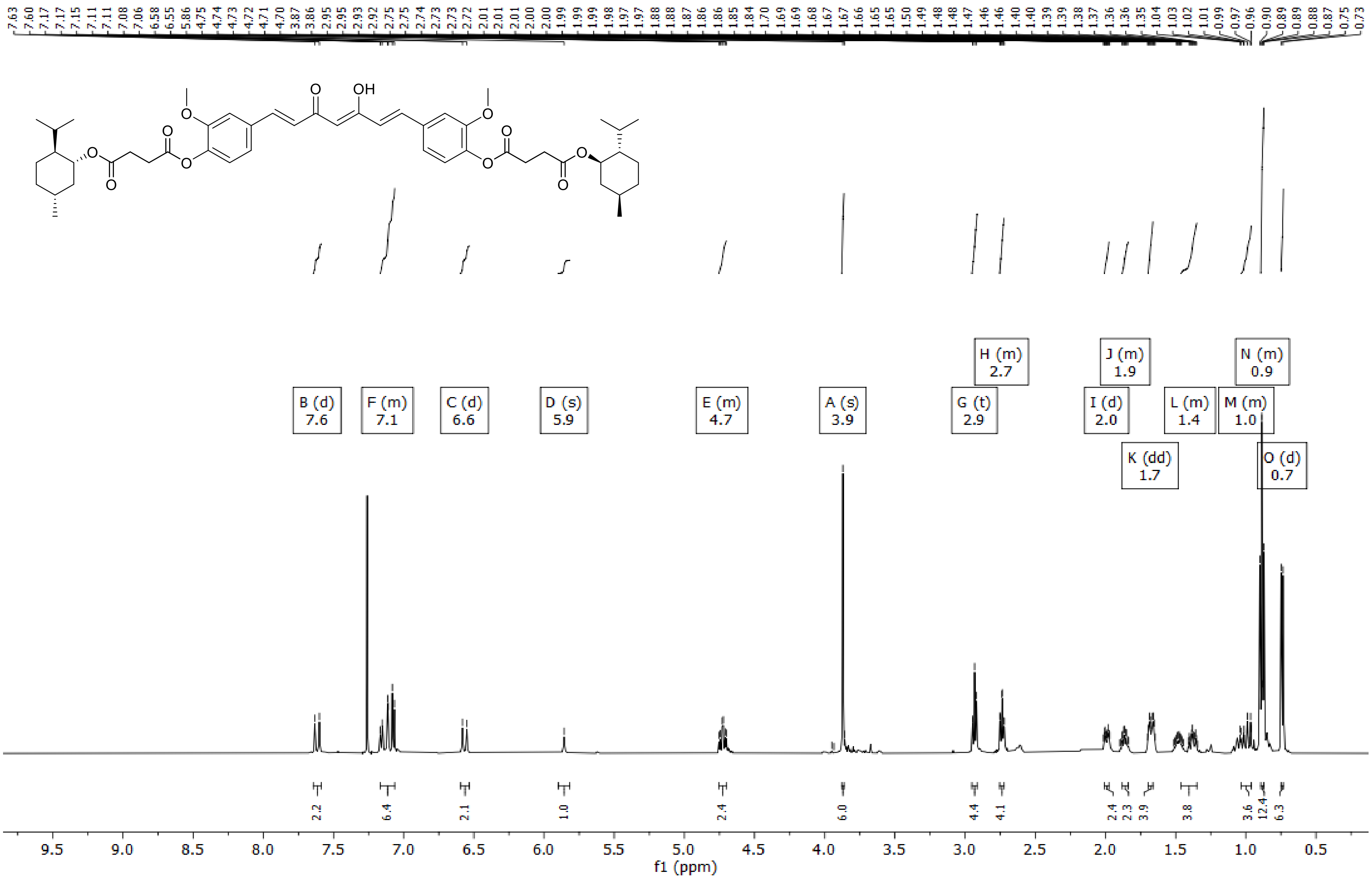

O,O′-(((1E,3Z,6E)-3-hydroxy-5-oxohepta-1,3,6-triene-1,7-diyl)bis(2-methoxy-4,1-phenylene)) bis((1R,2S,5R)-2-isopropyl-5-methylcyclohexyl) disuccinate (12).

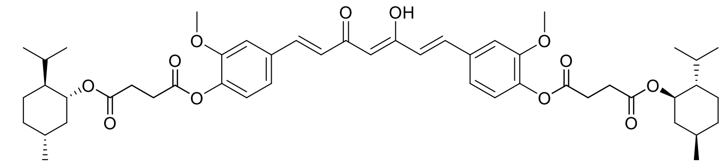

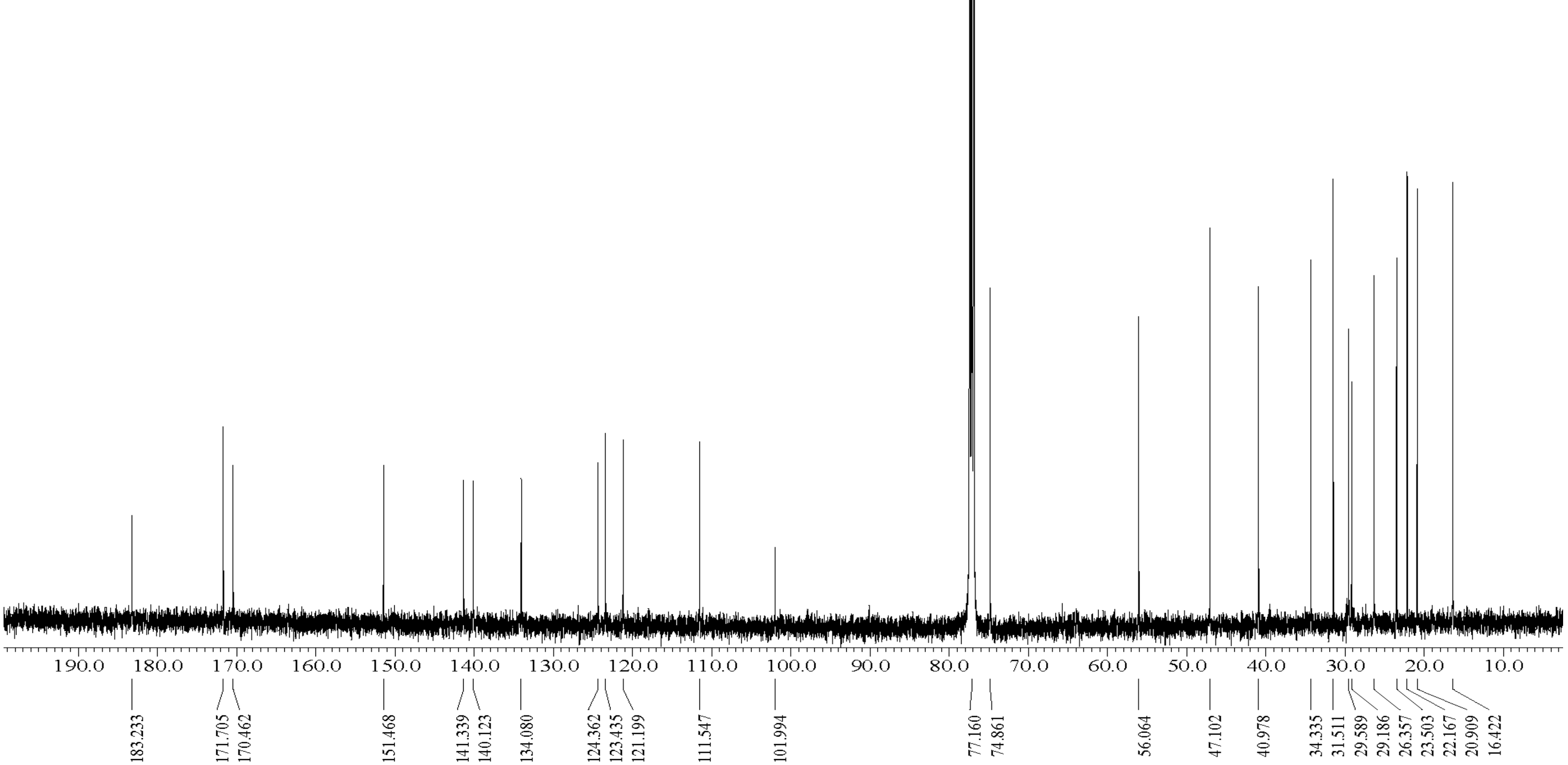

4-((1E,4Z,6E)-5-hydroxy-7-(4-hydroxy-3-methoxyphenyl)-3-oxohepta-1,4,6-trien-1-yl)-2-methoxyphenyl((1R,2S,5R)-2-isopropyl-5-methylcyclohexyl) succinate (13)

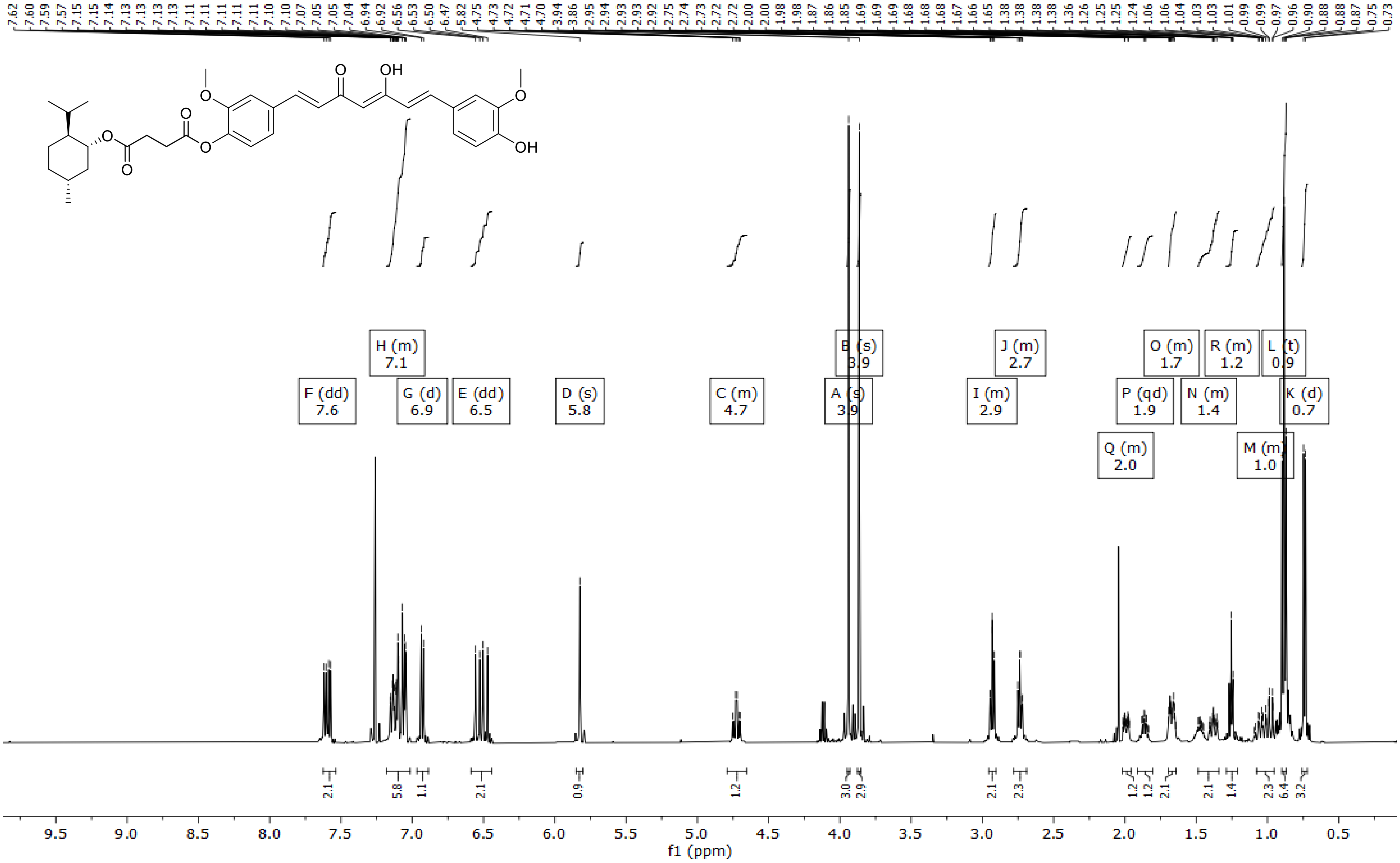

4-((1E,4Z,6E)-5-hydroxy-7-(4-hydroxy-3-methoxyphenyl)-3-oxohepta-1,4,6-trien-1-yl)-2-methoxyphenyl((1R,2S,5R)-2-isopropyl-5-methylcyclohexyl) succinate (13).

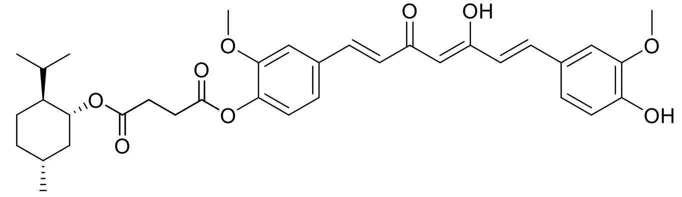

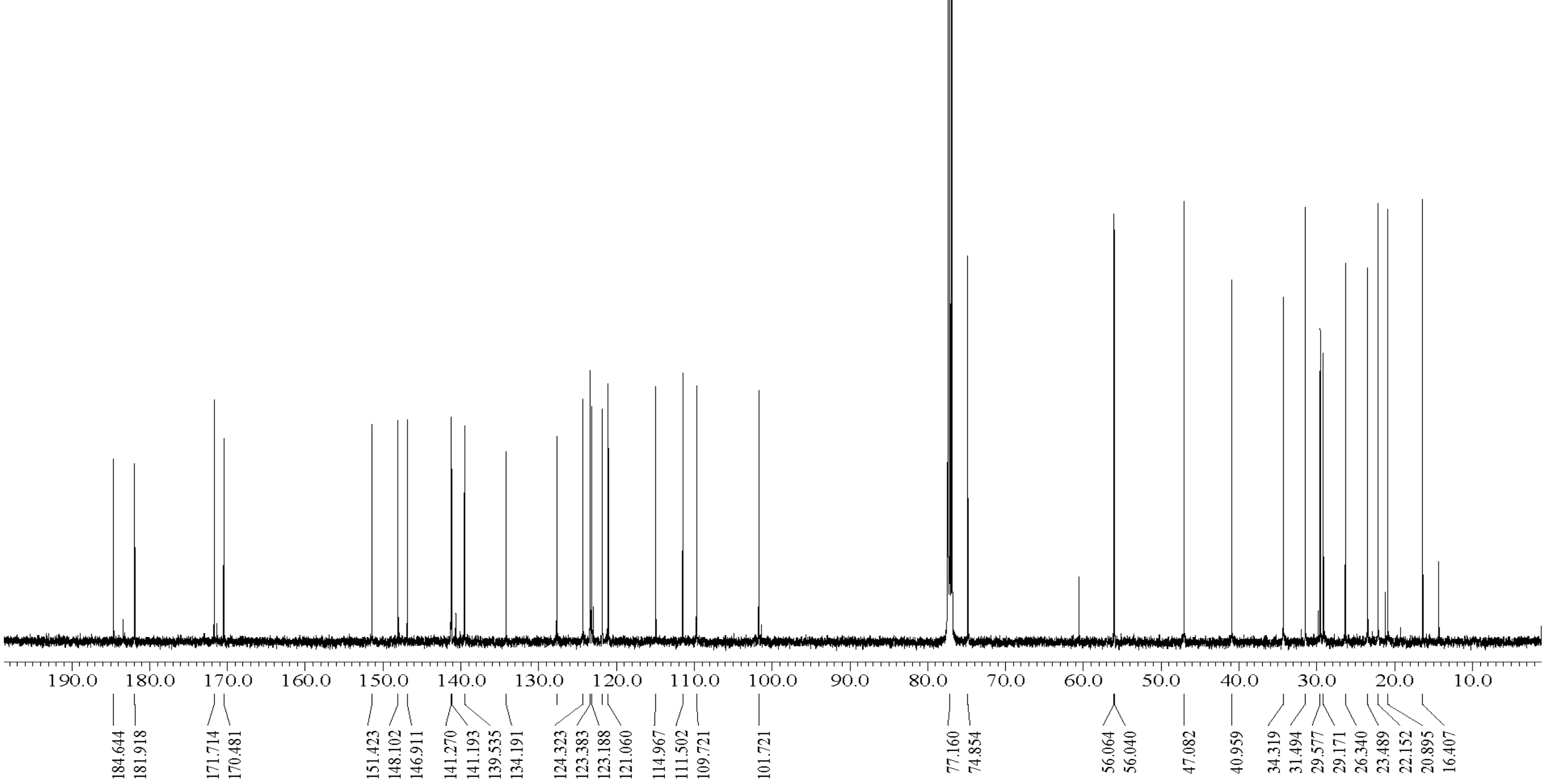

4-((1E,4Z,6E)-5-hydroxy-7-(4-hydroxy-3-methoxyphenyl)-3-oxohepta-1,4,6-trien-1-yl)-2-methoxyphenyl methyl succinate (14).

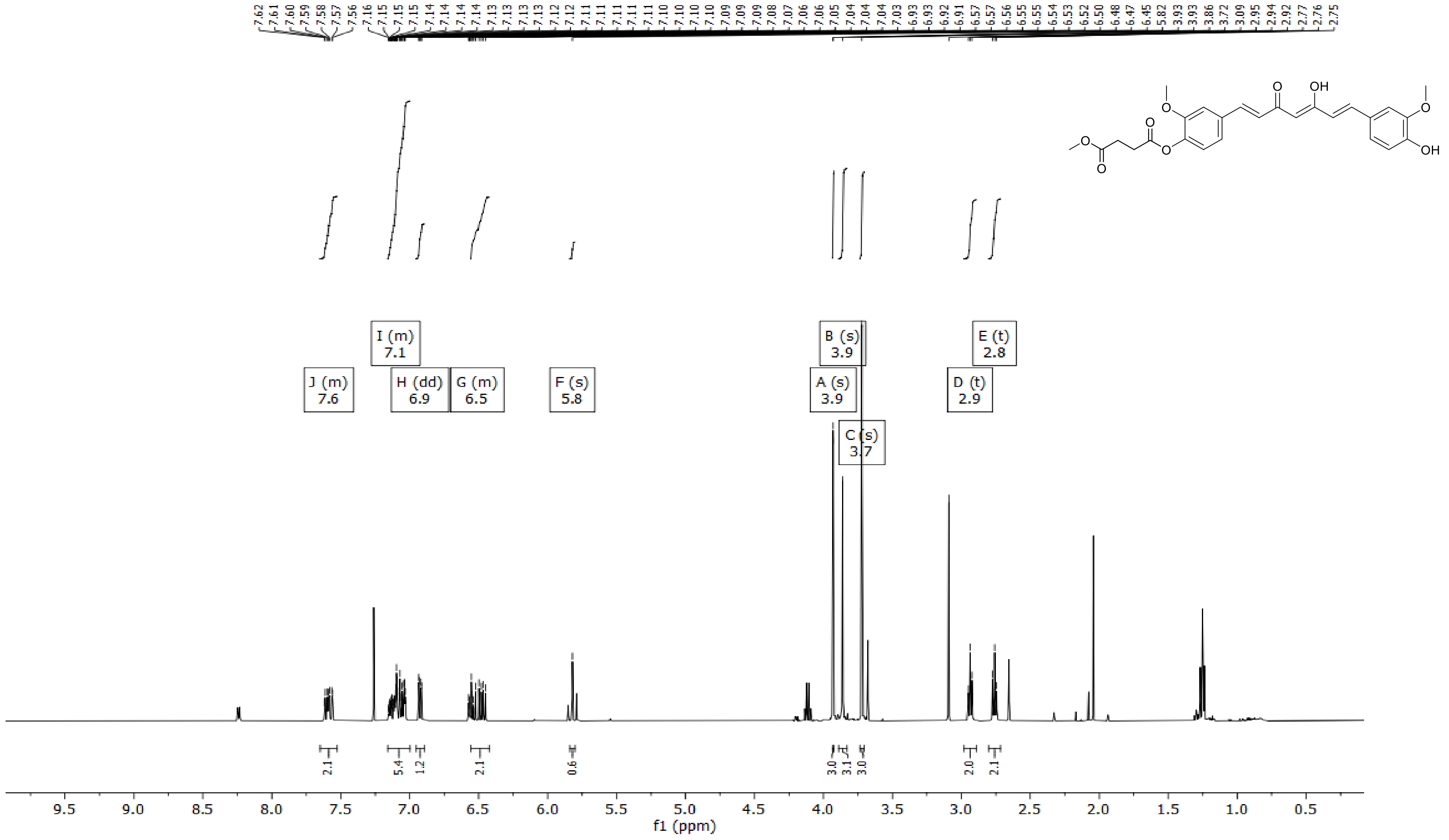

4-((1E,4Z,6E)-5-hydroxy-7-(4-hydroxy-3-methoxyphenyl)-3-oxohepta-1,4,6-trien-1-yl)-2-methoxyphenyl methyl succinate (14).

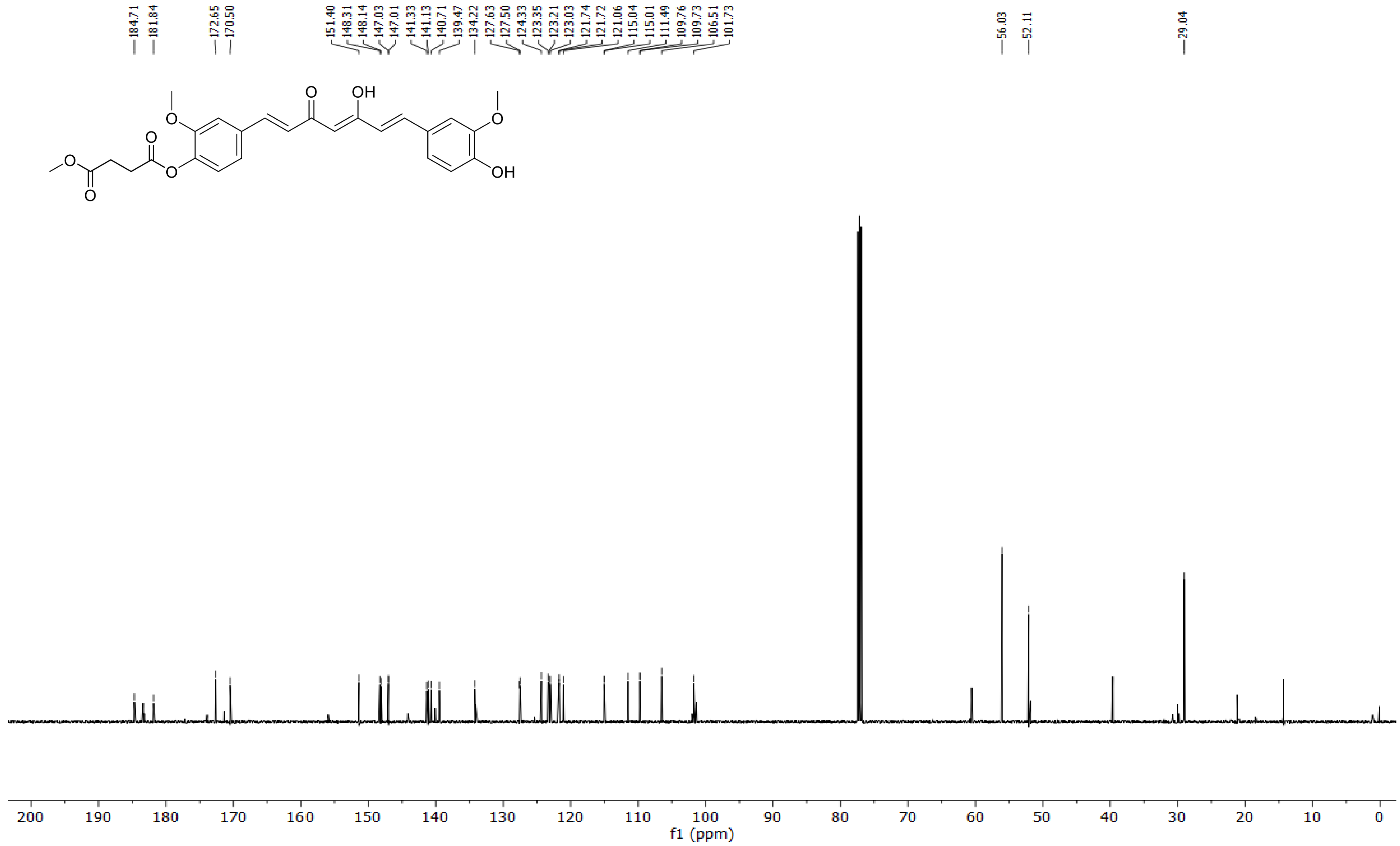

4-((1E,4Z,6E)-5-hydroxy-7-(4-hydroxy-3-methoxyphenyl)-3-oxohepta-1,4,6-trien-1-yl)-2-methoxyphenyl (1,2,3,4-tetrahydronaphthalen-1-yl) succinate (15).

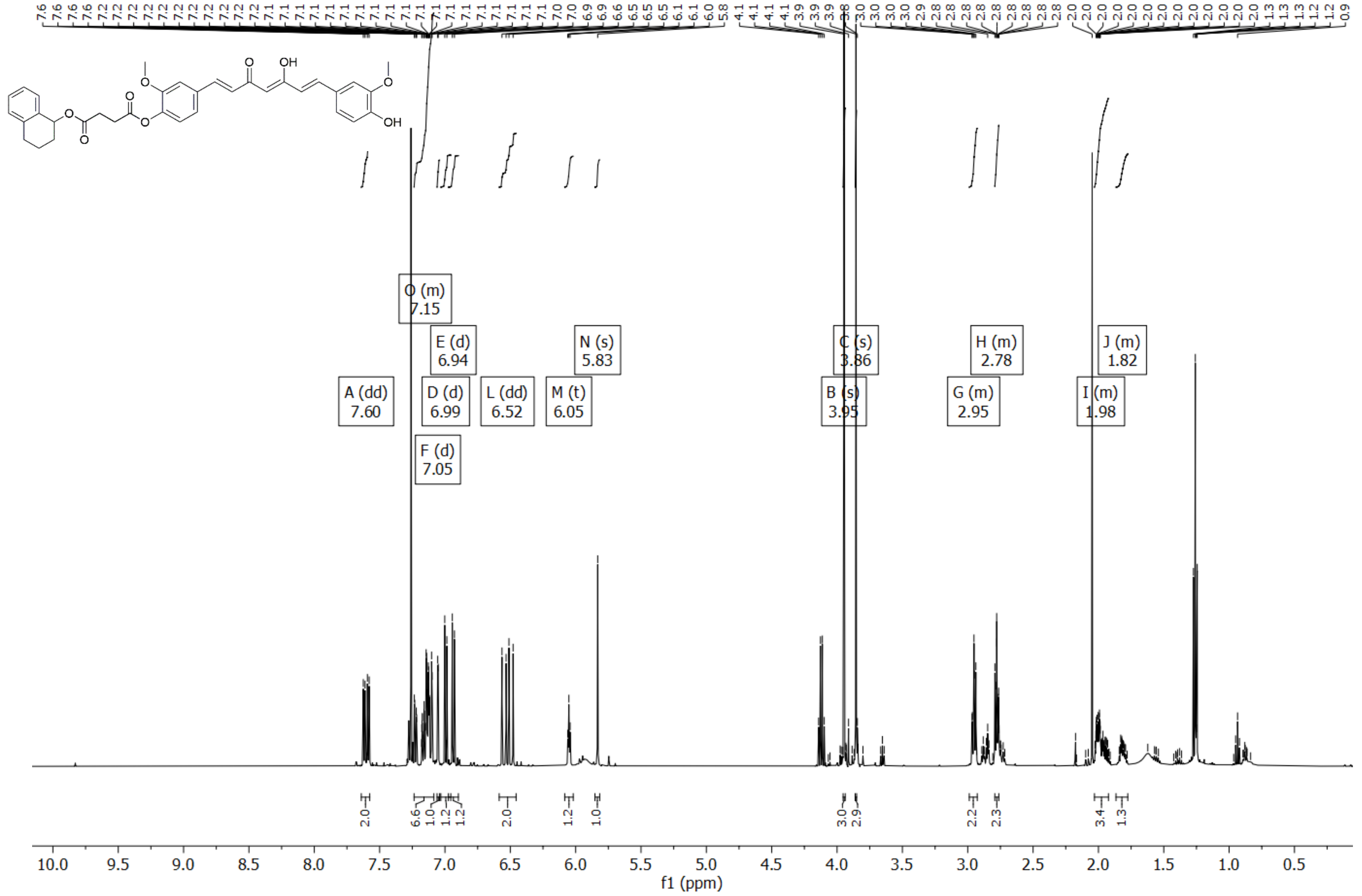

4-((1E,4Z,6E)-5-hydroxy-7-(4-hydroxy-3-methoxyphenyl)-3-oxohepta-1,4,6-trien-1-yl)-2-methoxyphenyl (1,2,3,4-tetrahydronaphthalen-1-yl) succinate (15).

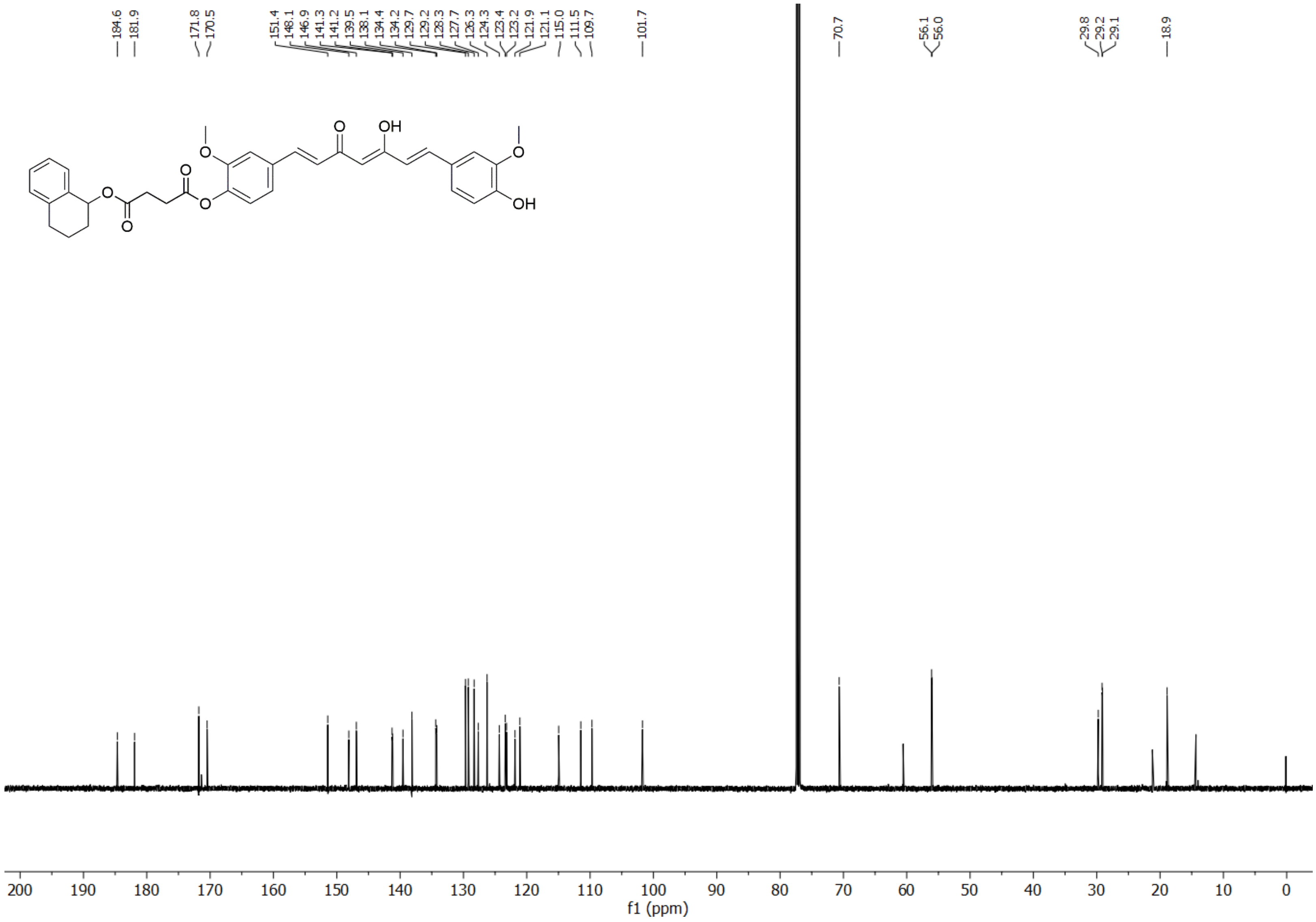

Refernces

1. Lakey-Beitia, J.; Gonzalez, Y.; Doens, D.; Stephens, D.E.; Santamaria, R.; Murillo, E.; Rao, K.S.; Larionov, O. V; Durant-Archibold, A.A. Assessment of Novel Curcumin Derivatives for the Prevention of the Inflammatory Process in Alzheimer's Disease. *J Alzheimer's Dis* **2017**, *2017*.
